# Supplementary material for: Focal seizures induce spatiotemporally organized spiking activity in the human cortex
Source: Nat Commun. 2024 Aug 16;15:7075. doi: 10.1038/s41467-024-51338-1 (PMC11329741; doi:10.1038/s41467-024-51338-1)
Supplement: Supplementary file 1 — Supplementary Information [file 41467_2024_51338_MOESM1_ESM.pdf]

Supplementary Tables

Patient ID	Sex	Age	Typical seizure types	MRI	Procedure	Pathology	Engel class	Time to outcome assessment (months)	Array features
1	M	34	FIAS, FTBTCS	L MTS	L ATL	HS, MDG	2b	11	96e L AT
2	M	24	FIAS, FTBTCS	NL	L ATL	MDG	2b	12	64e x 2 L AT
3	M	21	FIAS, FTBTCS	NL	R ATL	MDG	1a	28	96e R AT
4	M	32	FIAS, FTBTCS	B PVNH	L ATL	MDG	2b	24	96e L AT
5	M	29	FAS, FTBTCS	NL	RP topectomy	MDG	4	14	96e R P

Supplementary Table 1 Participant demographic information.

Abbreviations: e: electrode; FIAS: Focal impaired awareness seizure; F2BTCS: Focal to bilateral tonic-clonic seizures; R: right; L: left; B: bilateral; T: temporal; AT: anterior temporal; ATL: anterior temporal lobectomy; F: frontal; P: parietal; NL: No lesion; MTS: mesial temporal sclerosis; HS: hippocampal sclerosis; PVNH: periventricular nodular heterotopia; MDG: microdysgenesis

Patient ID	Array number	Number of seizures	Number of recruited seizures	Recruited seizure type				Non-recruited seizure type			
				SC	FAS	FIAS	FTB GTC	SC	FAS	FIAS	FTB GTC
1	1	7	6			5	1			1	
2	1	2	2				2				
	2	2	2				2				
3	1	1	1				1				
4	1	2	2			1	1				
5	1	6	2				2		4		

SC: subclinical; FAS: focal aware seizure; FIAS: focal impaired awareness seizure; FTBGTC: focal to bilateral generalized tonic clonic seizure

Supplementary Table 2 Participant seizure types and recruitment information.

For each participant, we note how many seizures were recruited to each MEA (see *Methods*), and the number of each type of seizure. All seizures with MEA recruitment were either focal impaired awareness seizures or focal to bilateral generalized tonic clonic seizures. No focal aware seizures recruited the array in any participant.

Patient ID	Array number	Interictal bursts	Interictal bursts after artifact removal	IEDs	IED-related bursts	Percent of burst with IED	Percent of IED with burst
1	1	271	165	729	44	26.67%	6.04%
2	1	1105	438	99	26	5.94%	26.26%
	2	101	53	170	31	58.49%	18.24%
3	1	1237	391	1089	264	67.52%	24.24%
4	1	1129	892	546	224	25.11%	41.03%
5	1	34	34	329	0	0%	0%

Supplementary Table 3 Spike burst and IED counts during interictal epochs.

We removed artifactual spike bursts (see *Methods*, Supplementary Figure 1) and then linked IEDs to spike bursts if both occurred in the same 150 ms period. Many spike bursts during interictal epochs occurred without an associated IED, and vice versa. We did not record any spike bursts with IEDs in participant 5.

Supplementary Figures

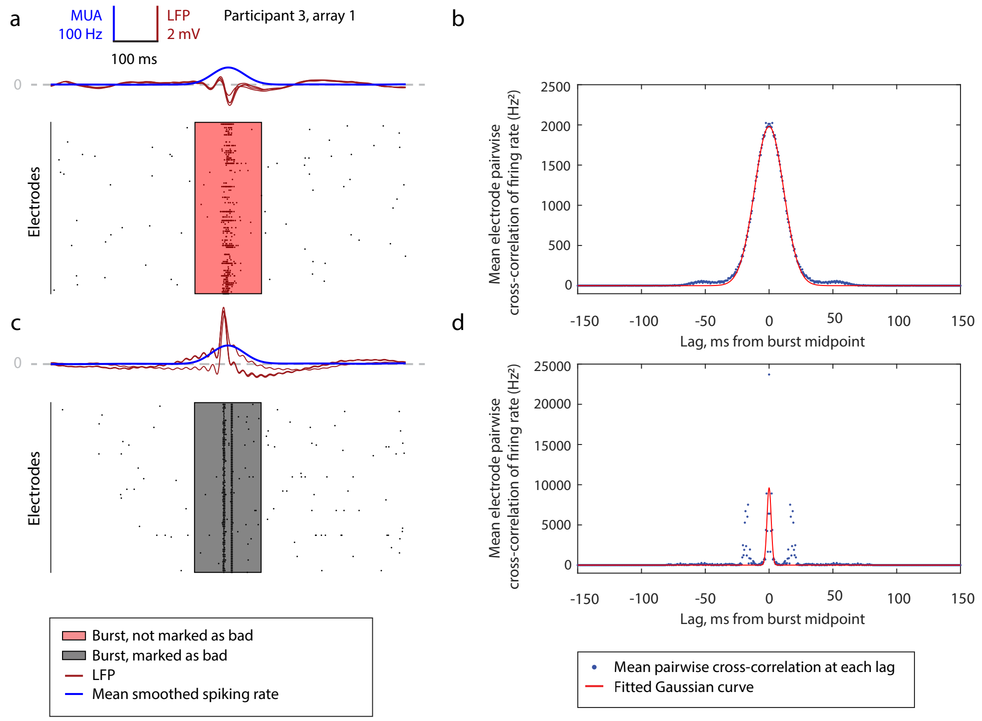

Supplementary Figure 1 Burst artifact rejection procedure.

In order to measure whether bursts were artifactual, we obtained the mean cross-correlation of burst rasters, across all pairs of micro-electrodes, and fitted this mean cross-correlation to a Gaussian (see *Methods*). The rationale was, in physiologic bursts (**a**), there was typically a smooth temporal gradient of action potential times, leading to a smooth mean cross-correlation that resembled a Gaussian (**b**). On the other hand, in many artifactual bursts, action potential times often coincided, and/or were temporally-displaced from each other (**c**), leading to erratically-shaped mean cross-correlations (**d**). We then captured the goodness of fit, or R^2, of the Gaussian (*red curves*) to the mean cross-correlation (*blue dots*). In other words, R^2 can be thought of as the similarity between the red curve and the blue dots. We rejected bursts as artifact if 1) the goodness of fit, R^2, to the model was less than 0.75, 2) the standard deviation of the Gaussian curve was $\leq$ 10 samples (5 ms), or both. **a)** An example physiologic IED-related burst is shown, for participant 3, who had a substantial amount of artifact (Supplementary Table 3). **b)** For this IED burst, $R^2 = 0.9968$ and $\sigma = 31.38$ samples. Criteria for rejection were not met and this burst was retained. **c)** An artifactual burst is shown, also for participant 3. In this burst, MUA (*bottom*) is recorded simultaneously in virtually every electrode. These events often re-occur multiple times within 10 to 20 ms of each other. The LFP (*top*) often shows accompanying artifact. The cause of this artifact is unclear. **d)** For this example burst, $R^2 = 0.42$ and $\sigma = 5.43$ samples. Therefore, both criteria for rejection were met and we discarded this burst.

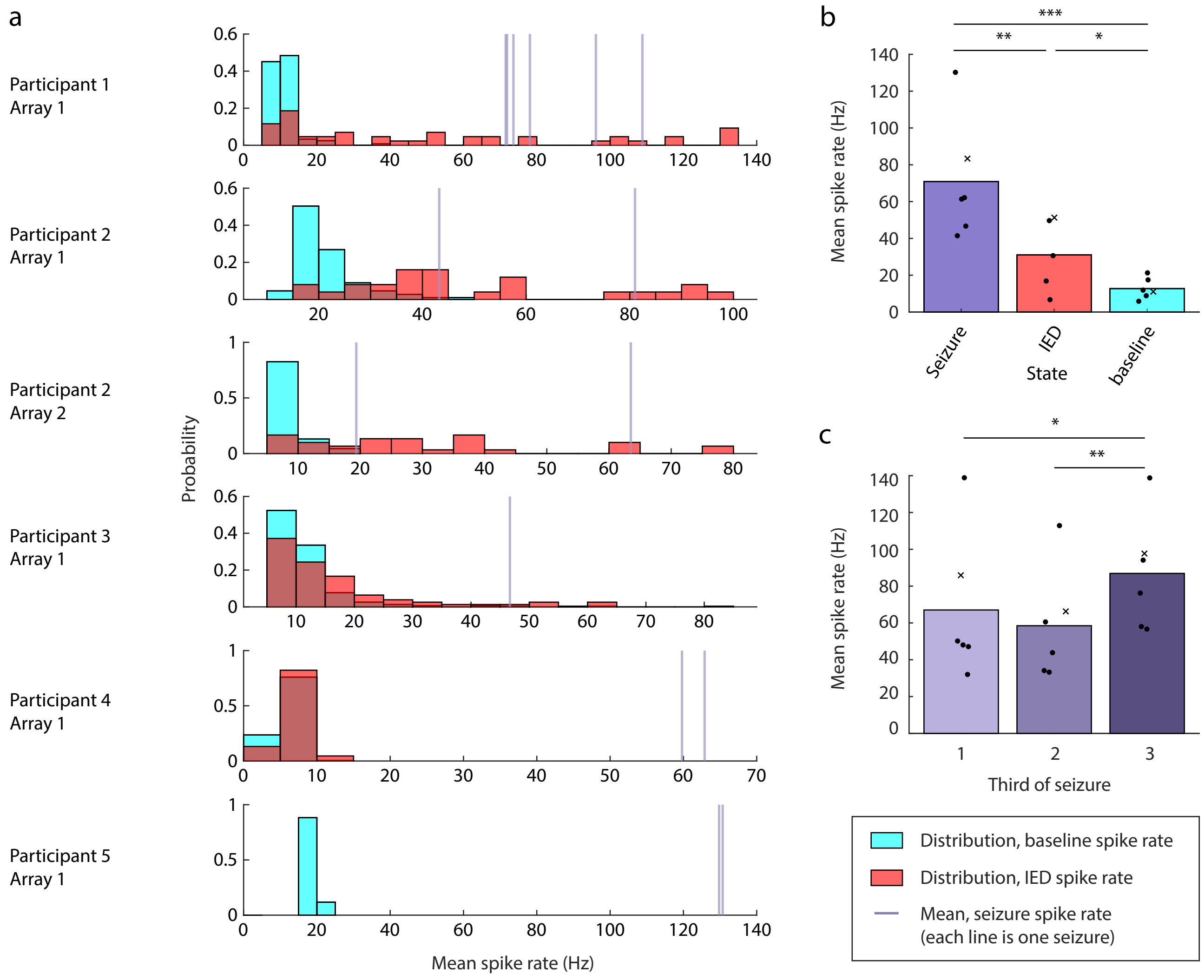

Supplementary Figure 2 Analysis of spike rates across states.

We were interested in comparing spike rates across state (baseline, IED, and seizure). **a)** For each participant and array, we collected all bursts in the baseline state (*cyan* histograms) and IED bursts (*red* histograms). Additionally, we collected all bursts for each particular seizure (*purple* vertical lines). For each particular burst, the spike rate was collected for each electrode, and then averaged across electrodes. Thus, for each burst, a single mean spike rate was produced. For IED and baseline bursts, each mean value was then put into the histogram to create the distributions shown. Bin width is 5 Hz. For seizure bursts, mean burst-level spike rates were then averaged across each burst in the seizure. Seizure spike rate tends to be the greatest, followed by IED spike rate, and baseline spike rate tends to be the lowest. **b)** Mean spike rates were taken, for each state, and for each participant and array. $n = 6$ arrays. Here, each dot represents a participant and array. Mean spike rate was 70.86 ± 32.55 for seizure, 31.02 ± 19.69 for IED, and 12.72 ± 5.67 for baseline. There was a significant difference between at least two groups ($F(2, 8) = 19.625$, partial $\eta^2 = 0.83$, $p < .001$, repeated measures one-way ANOVA). A post-hoc test

revealed a significant difference between the seizure group and the IED group ($p = .012$), the seizure group and the baseline group ($p < .001$), and the IED group and the baseline group ($p = .035$, p-values adjusted using the Bonferroni-Holm correction for multiple comparisons). **c)** Mean spike rates were taken, for each third of each seizure, and for each participant and array. $n = 6$ arrays. Each *black dot* represents a participant and array. Mean spike rate was 67.02 ± 39.46 for the first third, 58.48 ± 29.90 for the second third, and 86.92 ± 30.75 for the final third. There was a significant difference between at least two groups ($F(2, 10) = 11.73$, partial $\eta^2 = 0.70$, $p = .002$, repeated measures one-way ANOVA). A post-hoc test for multiple comparisons revealed a significant difference between the first and last thirds of the seizure ($p = .016$) and between the middle and last thirds of the seizure ($p = .002$). There is a tendency for spike rate to nadir at mid-seizure, and to increase in the final third of the event.

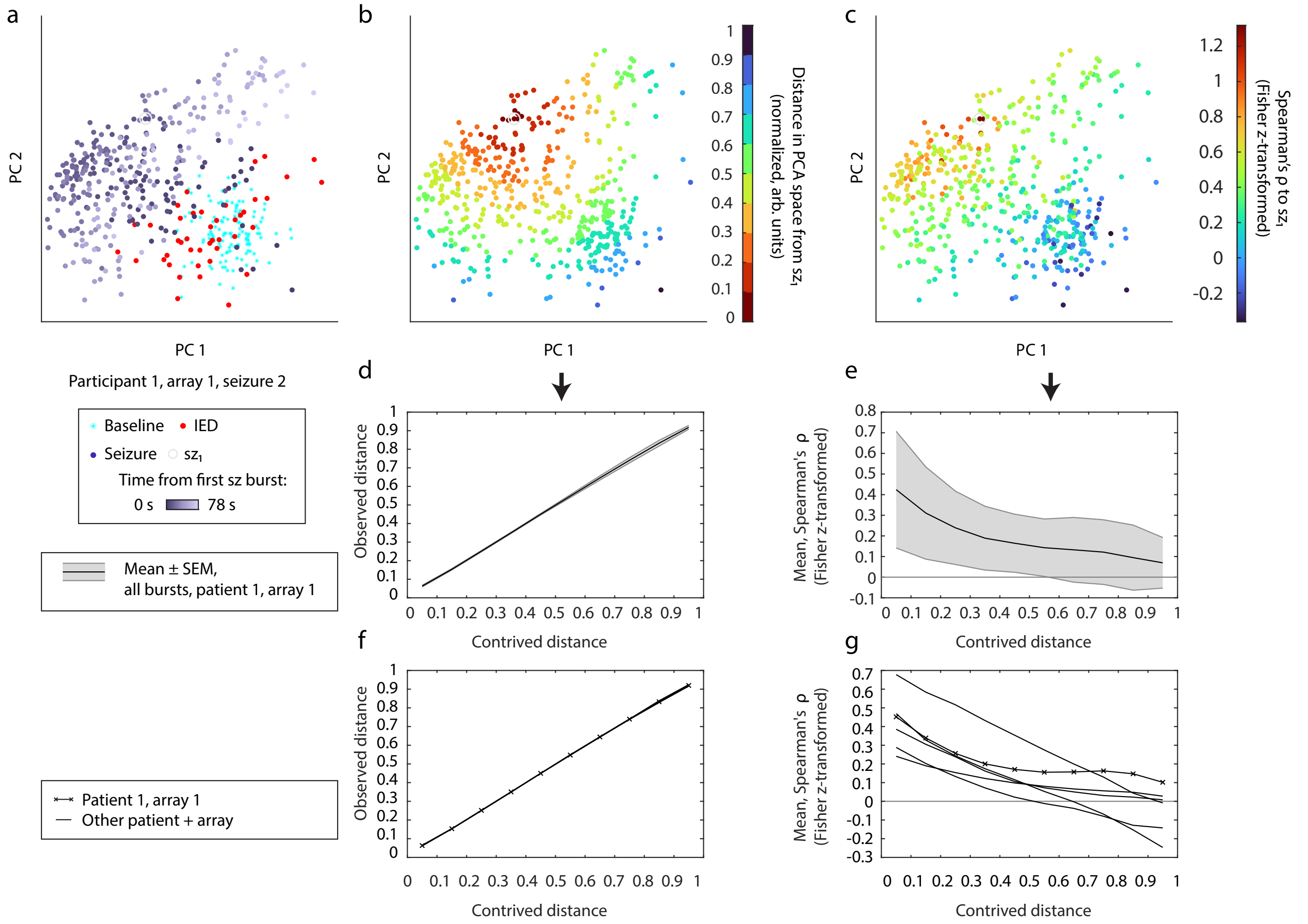

Supplementary Figure 3 Relationship between Spearman's ρ and distance in PCA space.

We were interested in exploring the relationship between Spearman's rank correlation coefficient and distance in principal component analysis (PCA) space. We reasoned that, as bursts became more dissimilar from each other, by Spearman's ρ, they should likewise become farther from each other in PCA space. **a)** Here, we have reproduced the low-dimensional decomposition of our spiking data (Figure 2a, see *Methods*). Each dot represents a single spike sequence during a baseline burst (*cyan*), IED burst (*red*), and seizure burst (colored *dark purple* to *light purple* based on the time following the first detected seizure burst). **b)** All bursts collected for this participant and seizure were again plotted in PCA space. Here, the seizure burst sz_1 was selected for analysis as an exemplary *target burst* (*gray circle*). This burst was also highlighted in Figures Figure 1, Figure 2, Figure 3, Figure 4, and Figure 5. From sz_1, we took the normalized distance to all other bursts in the dimensionality reduction, and placed those distances into 10 equally-sized bins. **c)** The same dimensionality reduction is shown, but bursts are colored according to Spearman's ρ (Fisher z-transformed) between sz_1 and every other burst. We can see, as hypothesized, that bursts near sz_1 in PCA space also often have warmer colors (ρ between those bursts and sz_1 is higher). **d)** The analysis in **b** was repeated for every burst in this PCA decomposition. For each target burst, and for each contrived distance bin (color groupings in **b**), we measured the actual distance between bursts in that

bin and the target burst. The *black line* and *gray shaded error bar* represent mean and standard deviation, respectively, of these distances, across all target bursts. The relationship between contrived distance, on the one hand, and observed distance, on the other, is linearly increasing, by construction. **e)** The analysis in **c** was repeated for every burst in this PCA decomposition. So, $n = 503$ bursts. For each target burst, and for each contrived distance bin (color groupings in **b**), we measured the mean value of ρ between bursts in that bin and the particular target burst. The *black line* and *gray shaded error bar* represent mean and standard deviation, respectively, of these ρ values, across all target bursts. As expected, as contrived distance (x-axis) becomes greater, Spearman's ρ (y-axis) decreases. **f)** The analysis in **d** was repeated, now for all participants and arrays, for $n = 6$ arrays. As expected by virtue of the construction of the analysis, normalized distance increases with increasing distance bins. **g)** The analysis in **e** was repeated, now for all participants and arrays, for $n = 6$ arrays. As hypothesized, ρ tends to decrease with increasing binned distance between bursts.

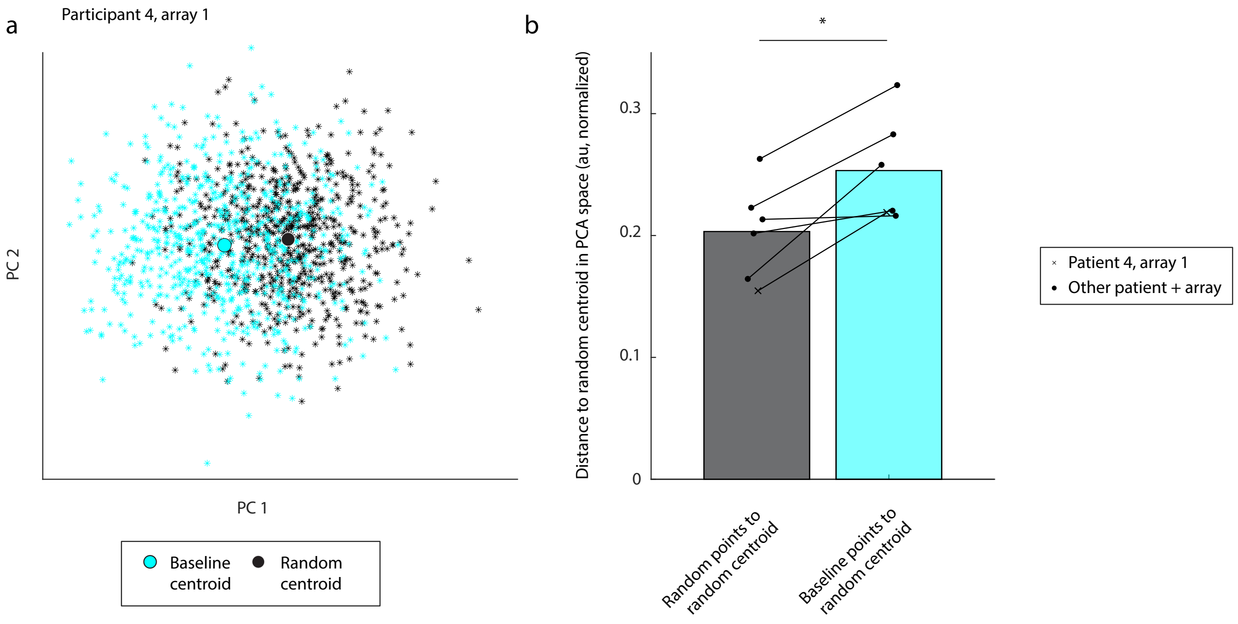

Supplementary Figure 4 Comparison between baseline sequences and random sequences.

In several of our analyses, we measure the distance of seizure bursts from baseline activity in PCA space (see Figure 2). We find evidence that seizure bursts are separate from baseline activity, and use this as evidence that seizures disrupt normal neuronal coding. For this claim to be cogent, it is required that the baselines we detect are, themselves, meaningful, as opposed to reflecting random activity or noise. Therefore, we were interested in comparing our recorded baseline bursts to random activity, to ensure our baseline bursts were distinct. **a)** In an example participant (participant 4), we collected all 695 baseline sequences, and for each sequence, also created a random null counterpart. The random null counterparts were created by shuffling the ordering of active spiking electrodes in each burst (see *Methods*). We then projected all baseline and random sequences into the same PCA space. Baseline burst sequences (*cyan*) are displaced from random burst sequences (*black*). We computed the baseline and random centroids in this space (*cyan* and *black* circles, respectively). Mean distance in PCA space, normalized to maximum distance in this decomposition, was 0.15 ± 0.09 from random burst to the random centroid and 0.22 ± 0.11 from baseline burst to random centroid centroid. **b)** In every participant in every array ($n = 6$ arrays), we computed the mean distance between all random null sequences and the random null centroid, and the mean distance between all baseline burst sequences and the random null centroid. On average, baseline burst sequences were significantly displaced from the random null centroid, compared to the random burst sequences (distance of 0.25 ± 0.04 versus 0.20 ± 0.04; $t(5) = 3.69$; Cohen's $d = 1.01$; $p = .014$, paired t-test).

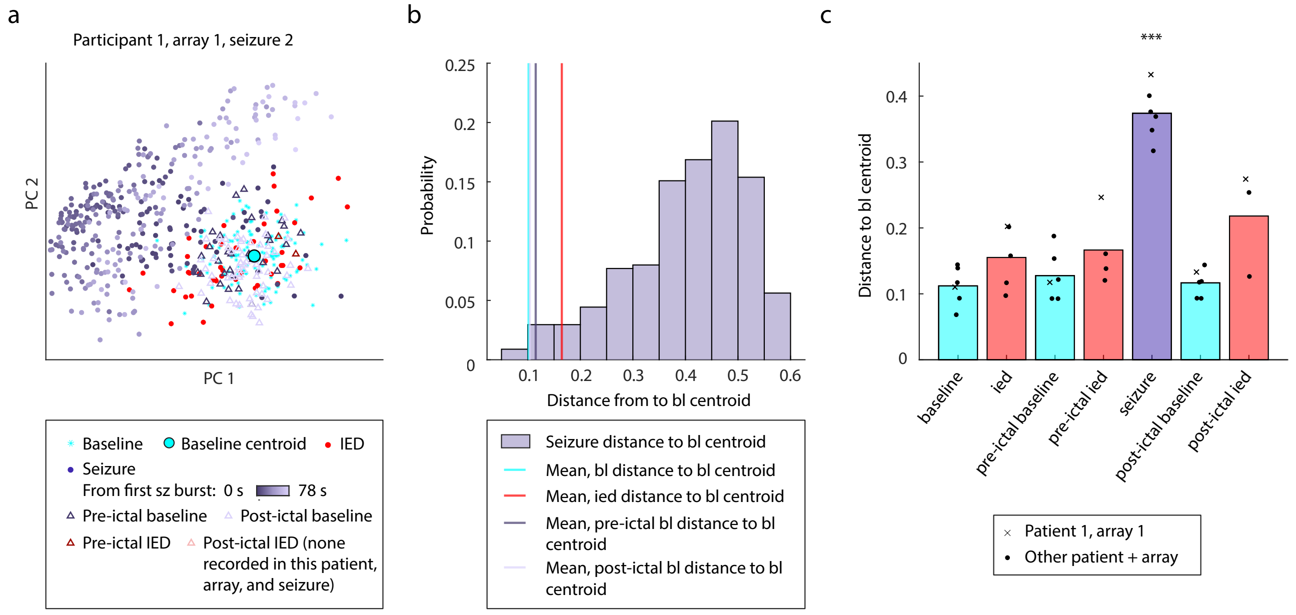

Supplementary Figure 5 Properties of pre- and post-ictal burst sequences.

In the analyses in the main manuscript, interictal epochs (which contain baseline and IED bursts) were separated from ictal epochs by at least 6 hours (see *Methods*). This, however, could mask pathological entrainment occurring immediately before or after seizure events. Therefore, we performed an additional analysis, where, alongside the usual method for collecting baseline and IED bursts, we collected an additional set of bursts: those found in ictal *epochs* (which are 2-3 hours long) but which do *not* actually transpire during the seizure event. Therefore, those bursts which occurred in the ictal epoch, but prior to the seizure, were called pre-ictal bursts. Those, furthermore, could be classified as pre-ictal baseline bursts or pre-ictal IED bursts, depending on whether or not there was an associated interictal discharge. Likewise, we collected post-ictal baseline and post-ictal IED bursts, which occurred during ictal epochs but after the seizure event. We were curious as to whether there may be either a ramp-up or ramp-down phenomenon, wherein pathological entrainment of baseline activity may begin before seizure onset, or may persist after seizure termination. **a)** We repeated our dimensionality reduction analysis (see *Methods*) on participant 1, array 1, seizure 2 (the same participant and seizure considered in the figures in the main manuscript.) This time, however, we included pre-ictal and post-ictal baseline and IED bursts in the dimensionality reduction. These bursts are indicated by *triangles* (*dark purple* triangle: pre-ictal baseline; *light purple* triangle: post-ictal baseline; *dark red* triangle: pre-ictal IED; *light red* triangle: post-ictal IED, none found for this particular participant, array and seizure). Overall, dimensionality reduction results are similar to those in the main analysis. The pre- and post-ictal bursts tend to congregate near the baseline bursts in the low-dimensional subspace. **b)** Here, we have shown the distribution of distances of seizure activity from the baseline centroid (*purple* histogram). Additionally, in the same distribution, we have shown the mean distance of non-seizure bursts to the baseline centroid (*light blue* line: baseline bursts; *red* line: IED

bursts; *dark purple* line: pre-ictal baseline bursts; *light purple* line: post-ictal baseline bursts). Pre- and post-ictal IED bursts are not included in **b** because few or none of these bursts were recorded in this ictal epoch. All four types of non-seizure burst reside relatively close to each other, in terms of distance to the baseline centroid. All four are substantially closer to the baseline centroid than the seizure bursts. **c)** Summary data for pre- and post-ictal burst distance to baseline centroid. The analyses in **a** and **b** were repeated for all participants, arrays and seizures. Results were averaged across seizures, so that each dot represents a participant and array ($n = 6$ arrays). For baselines, mean distance to the baseline centroid was 0.11 ± 0.03. For IEDs, mean distance to the baseline centroid was 0.16 ± 0.05. Distance to baseline centroid was 0.13 ± 0.04 and 0.17 ± 0.06 for pre-ictal baselines and pre-ictal IEDs, respectively. For seizure bursts, mean distance to the baseline centroid was 0.37 ± 0.04. Distance to baseline centroid was 0.12 ± 0.02 and 0.22 ± 0.08 for post-ictal baselines and post-ictal IEDs, respectively. There was a significant difference between at least two groups ($F(6, 12) = 16.34$, partial $\eta^2 = .89$, $p =< .001$, repeated measures one-way ANOVA). A post-hoc test revealed a significant difference between the seizure group and all other groups ($p < .001$, Bonferroni-Holm correction for multiple comparisons). There was no significant difference for any other comparison. Though it does not reach statistical significance, there is a trend towards increased displacement among IEDs, and in particular, post-ictal IEDs. This could suggest residual pathological entrainment of these IED sequences following seizure, but further research in a larger cohort is required to support this finding.

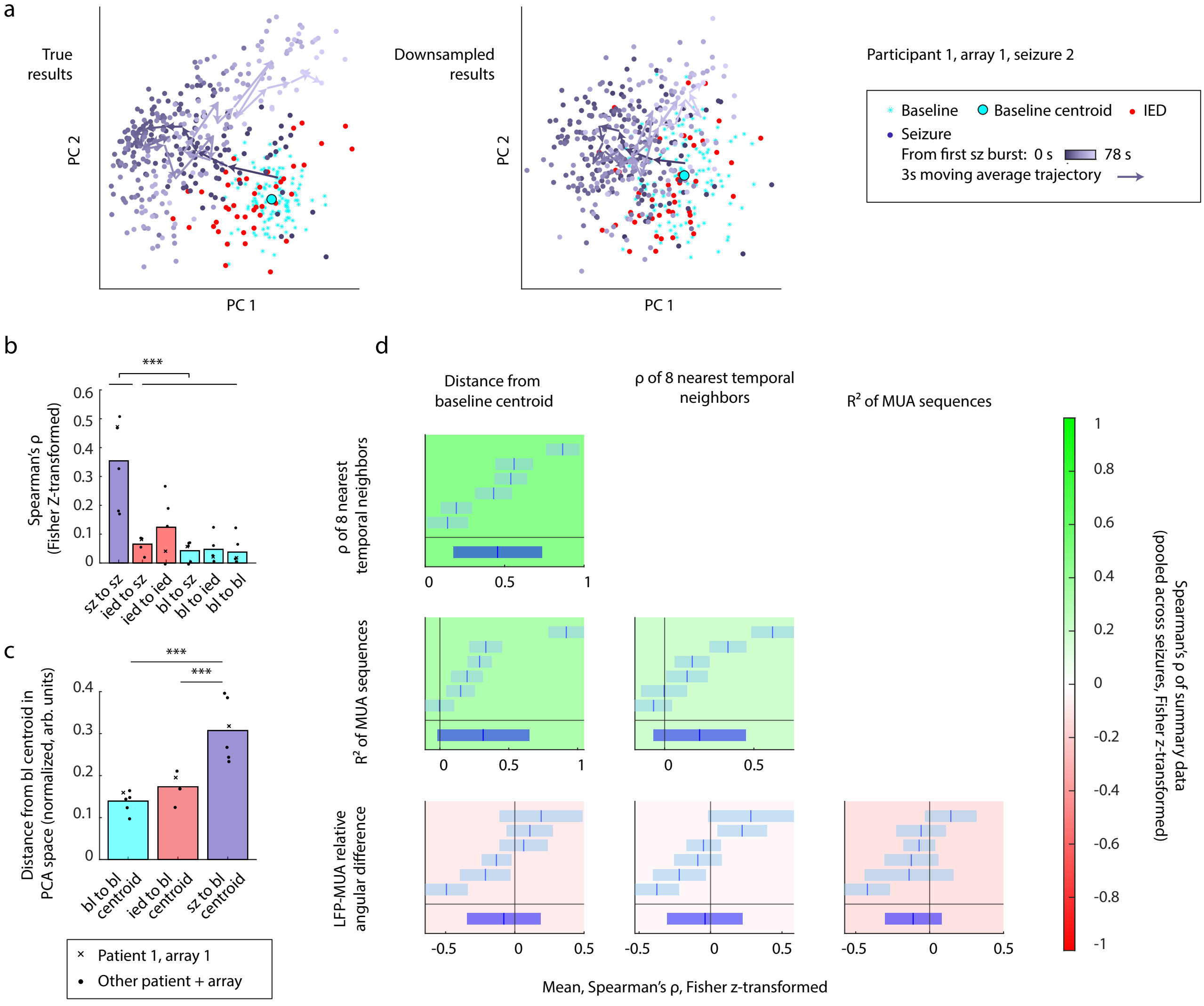

Supplementary Figure 6 Results are similar after downsampling to account for differences in spike rate

There was considerable variability of spike rate with burst state (seizure, IED, and baseline states; Supplementary Figure 2). We wanted to ensure that differences in spike rate were not confounding our results, which are based fundamentally on differences in spike *sequences* across states. To this end, for each participant, we downsampled spiking rates at the level of the burst, for IED and seizure bursts. Baseline bursts, which had the lowest spiking rates (Supplementary Figure 2a, c) were left unchanged.

Our method for downsampling was as follows. For each particular participant and array, the set of baseline bursts was collected. For each burst, the number of electrodes exhibiting MUA was counted. This was repeated for all k baseline bursts. The set of k electrode counts were then put into a distribution; this distribution tended to be roughly normally-distributed. Then, a Gaussian curve was fit to this distribution. Next, seizure bursts were downsampled, for the same participant and array. To do this, for each seizure

burst, we drew an electrode count at random from the Gaussian distribution built from the baseline counts. For the particular seizure burst in question, spiking electrodes were removed at random (with MUA timestamps replaced by NaN) until the number of remaining spiking electrodes was equal to that randomly-drawn electrode count. This process was repeated for all seizure bursts. The same process was then carried out for IED bursts. Finally, the entire process was repeated for each participant and array. In this way, we ensured that, among seizure, IED, and baseline bursts, the number of electrodes exhibiting MUA was roughly equal, by construction. Further analyses could then be performed on those downsampled bursts, to ensure that results did not arise from inequalities in spike rate.

a) *Left:* We again performed dimensionality reduction in a single example participant and seizure (participant 1, seizure 2). This panel shows the same data as shown in Figure 2a. *Right:* Here, the same analysis was performed, but after downsampling seizure and IED bursts, as described above. The trend in which seizure bursts are displaced from the baseline centroid is attenuated, but it is still evident.

b) We then repeated several of our core analyses, now with the downsampled structures. First, we considered whether internal consistency, measured by Spearman's ρ, was greatest among seizure bursts (the same analysis as in Figure 1f). Each dot represents an array ($n = 6$ arrays). Similarity (Spearman's ρ) was 0.35 ± 0.15 for seizure to seizure, 0.07 ± 0.03 for IED to seizure, 0.12 ± 0.11 for IED to IED, 0.04 ± 0.03 for baseline to seizure, 0.05 ± 0.05 for baseline to IED, and 0.04 ± 0.05 for baseline to baseline. These values are very similar to those seen in the main analysis. Here, spike sequence similarity among seizure bursts was significantly greater than similarity in any other comparison, including comparisons between and across groups ($F(5, 20) = 21.52$, partial $\eta^2 = 0.84$, $p < .001$, repeated measures one-way ANOVA; $p < .001$, Holm-Bonferroni correction for multiple comparisons). **c)** We then asked whether seizure bursts, after dimensionality reduction, were significantly displaced from baseline bursts (the same analysis as in Figure 2b). Each dot represents an array ($n = 6$ arrays). Here, baseline distance from baseline centroid was 0.14 ± 0.03, IED distance from baseline centroid, 0.17 ± 0.03, and seizure distance from baseline centroid, 0.31 ± 0.07. There was a significant effect of burst state (seizure, IED, or baseline) on distance from the baseline centroid ($F(5, 20) = 24.98$, partial $\eta^2 = .86$, $p < .001$, repeated measures one-way ANOVA). There was a significant difference between seizure bursts and IED bursts ($p = .001$) and between seizure bursts and baseline bursts ($p < .001$, Holm-Bonferroni correction for multiple comparisons).

d) Next, we compared the following measures to each other: distance of seizure bursts to the baseline centroid; Spearman's ρ between seizure bursts and 8 nearest temporal neighbors (a time-evolving measure of consistency); R^2 of seizure bursts (a measure of goodness of fit to spatial linear regression, indicating the directional nature of sequences); and the angular difference between MUA and LFP discharge directions. This is the same analysis done in Supplementary Figure 9. The results were as follows. Correlation between distance to baseline centroid and ρ of 8 nearest neighbors: $0.46 \pm 0.27; t(5) = 4.2, p = .008$, one-sample t-test. Correlation between distance to baseline centroid and R^2: $0.31 \pm 0.32; t(5) = 2.42, p = .06$, one-sample t-test. Correlation between distance to baseline centroid and angular difference between MUA and LFP discharge directions: $-0.08 \pm 0.25; t(5) = 0.767, p = .48$, one-sample t-test. Correlation between ρ of 8 nearest neighbors and R^2: $0.20 \pm 0.25; t(5) = 1.94, p = .11$ one-sample t-test. Correlation between ρ of 8

nearest neighbors and angular difference between MUA and LFP discharge directions: $-0.04 \pm 0.25; t(5) = 0.372, p = .73$, one-sample t-test. Correlation between R^2 and angular difference between MUA and LFP discharge directions: $-0.11 \pm 0.18; t(5) = 1.5, p = .19$, one-sample t-test. These results are very similar to those in the main manuscript. The only material difference is in the comparison between distance to baseline centroid and R^2 (the same analysis as that presented in Figure 4f). In the downsampled analysis, $p = .06$, compared to $p = .021$ in the true analysis. Otherwise, results are fundamentally similar, and there does not appear to be any meaningful confound introduced by differences in spike rate.

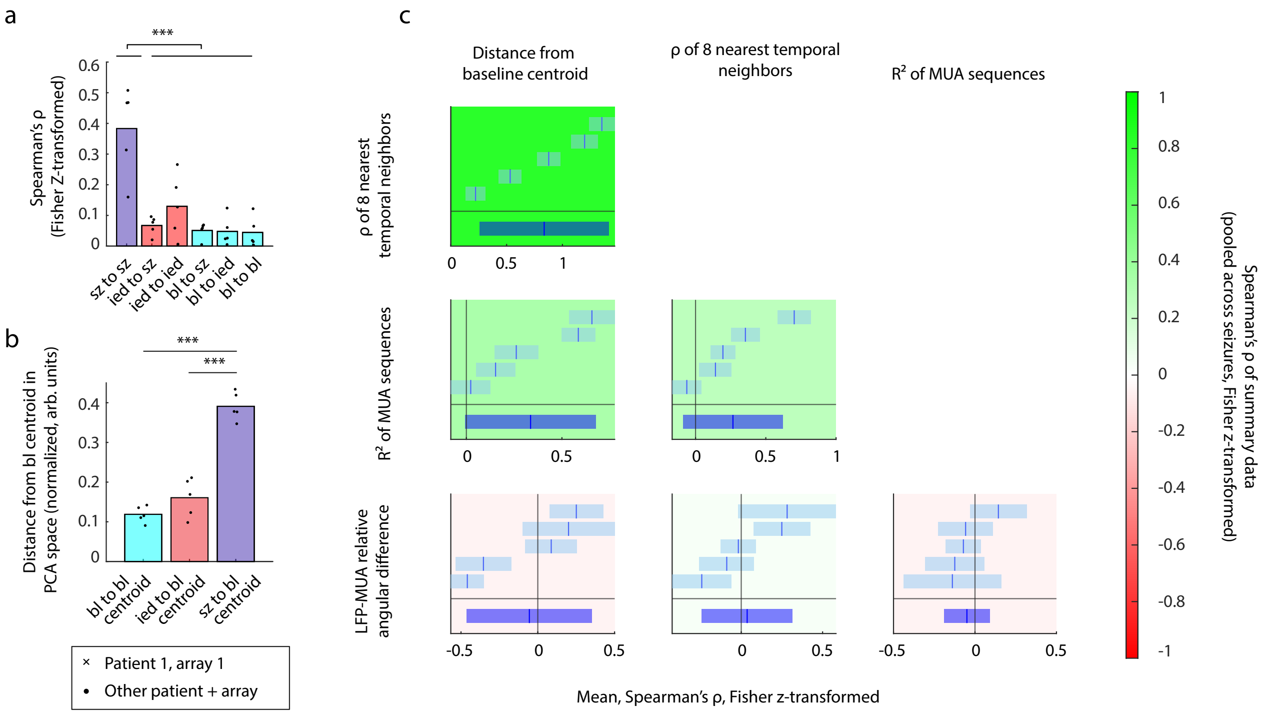

Supplementary Figure 7 Results are similar after eliminating the patient with the parietal array

Participants 1-4 underwent placement of temporal lobe micro-electrode arrays (see *Methods*). Participant 5, on the other hand, had a right parietal array placed (Supplementary Table 1). This patient was unusual in several respects. First, it was the only to have a parietal array and resection. Second, it was the only patient to have an Engel 4 outcome. Third, no burst-related IEDs were detected in this patient, while they were detected in all other patients. For these reasons, we wanted to ensure that our findings did not depend on this patient. So, we repeated several of our core analyses, this time with patient 5 excluded.
a) We considered whether internal consistency, measured by Spearman's ρ, was greatest among seizure bursts (the same analysis as in Figure 1f). Each dot represents an array ($n = 6$ arrays). Similarity (Spearman's ρ) was 0.38 ± 0.15 for seizure to seizure, 0.07 ± 0.03 for IED to seizure, 0.13 ± 0.10 for IED to IED, 0.05 ± 0.03 for baseline to seizure, 0.05 ± 0.05 for baseline to IED, and 0.04 ± 0.05 for baseline to baseline. These values are very similar to those seen in the main analysis. Here, spike sequence similarity among seizure bursts was significantly greater than similarity in any other comparison, including comparisons between and across groups ($F(5, 20) = 20.28$, partial $\eta^2 = 0.84$, $p < .001$, repeated measures one-way ANOVA; $p < .001$, Holm-Bonferroni correction for multiple comparisons). **b)** We then asked whether seizure bursts, after dimensionality reduction, were significantly displaced from baseline bursts (the same analysis as in Figure 2b). Each dot represents an array ($n = 6$ arrays). Here, baseline distance from baseline centroid was 0.12 ± 0.02, IED distance from baseline centroid, 0.16 ± 0.05, and seizure distance from baseline centroid, 0.39 ± 0.04. There was a significant effect of burst state (seizure, IED, or baseline) on distance

from the baseline centroid ($F(5, 20) = 119.7$, partial $\eta^2 = 0.97$, $p < .001$, repeated measures one-way ANOVA). There was a significant difference between seizure bursts and IED bursts ($p = .001$) and between seizure bursts and baseline bursts ($p < .001$, Holm-Bonferroni correction for multiple comparisons).

c) We compared the following measures to each other: distance of seizure bursts to the baseline centroid; Spearman's ρ between seizure bursts and 8 nearest temporal neighbors (a time-evolving measure of consistency); R^2 of seizure bursts (a measure of goodness of fit to spatial linear regression, indicating the directional nature of sequences); and the angular difference between MUA and LFP discharge directions. This is the same analysis done in Supplementary Figure 9. The results were as follows. Correlation between distance to baseline centroid and ρ of 8 nearest neighbors: $0.84 \pm 0.47; t(4) = 4.01, p = .016$, one-sample t-test. Correlation between distance to baseline centroid and R^2: $0.34 \pm 0.28; t(4) = 2.73, p = .052$, one-sample t-test. Correlation between distance to baseline centroid and angular difference between MUA and LFP discharge directions: $-0.05 \pm 0.33; t(4) = 0.374, p = .73$, one-sample t-test. Correlation between ρ of 8 nearest neighbors and R^2: $0.27 \pm 0.29; t(4) = 2.08, p = .11$ one-sample t-test. Correlation between ρ of 8 nearest neighbors and angular difference between MUA and LFP discharge directions: $0.04 \pm 0.22; t(4) = 0.362, p = .74$, one-sample t-test. Correlation between R^2 and angular difference between MUA and LFP discharge directions: $-0.05 \pm 0.11; t(4) = 0.979, p = .38$, one-sample t-test. These results are very similar to those in the main manuscript. The only material difference is in the comparison between distance to baseline centroid and R^2 (the same analysis as that presented in Figure 4f). In the downsampled analysis, $p = .052$, compared to $p = .021$ in the true analysis. Otherwise, results are fundamentally similar. Therefore, our results do not depend in a meaningful way on the participant with the parietal array.

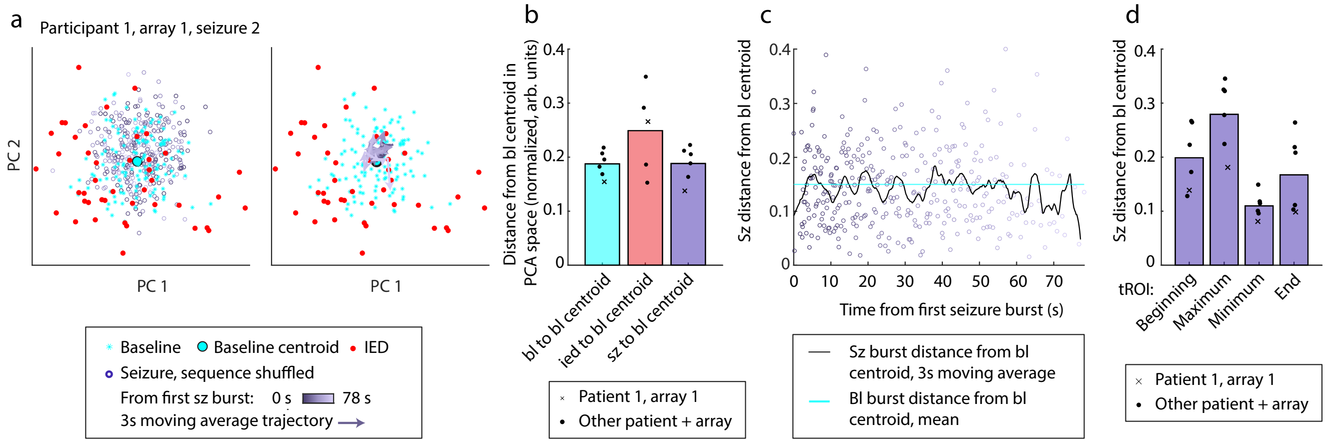

Supplementary Figure 8 Projection of random null versions of seizure spike sequences onto two-dimensional PCA space.

In Figure 2, we analyzed the relationship between seizure bursts and baseline activity in a low-dimensional subspace. Here, we repeated this analysis, but instead of using seizure bursts, we used a random null version of seizure bursts, where the MUA timings among active spiking electrodes were shuffled. Baseline and IED bursts were left as is, and null versions were not created for these bursts. **a)** *Left*: We used PCA to decompose high-dimensional sequence data into a low-dimensional subspace. Each dot represents a single spike sequence during a baseline burst (*cyan*), IED burst (*red*), and seizure burst in which we randomly shuffled the spiking sequence (colored *dark purple* to *light purple* based on the time following the first detected seizure burst). We identified the centroid of the baseline bursts in PCA space (*cyan circle*). *Right*: The results in the *left* panel were reproduced, except, instead of indicating seizure bursts with *purple dots*, we instead used *purple arrows* to indicate the trajectory of the seizure over time. Here, the seizure was broken up into three-second bins. In each bin, the mean location in the 2-d subspace was computed. Each arrow reflects this mean location, in two consecutive time bins, with the head of the arrow indicating the later time bin. In contrast to the results in Figure 2a, the shuffled seizure bursts do not follow a clear trajectory. **b)** Average distance between baseline bursts and the baseline centroid (0.19 ± 0.02, *cyan* bar), between IED bursts and the baseline centroid (0.25 ± 0.08, *red* bar), and between shuffled seizure bursts and the baseline centroid (0.19 ± 0.03, *purple* bar) in every participant and array. Each *black dot* represents a single participant and array ($n = 6$ arrays). There is no effect of state on distance to the baseline centroid ($p = .11$, repeated measures one-way ANOVA). **c)** Normalized Euclidean distance in PCA space between shuffled seizure bursts and the baseline centroid over the course of this example seizure (three second moving average in *black*). Each *black dot* represents a single participant and array ($n = 6$ arrays). **d)** Initial, maximum, minimum, and final distances between shuffled seizure bursts and baseline centroids in each participant and array (initial distance 0.20 ± 0.06; maximum distance 0.28 ± 0.06; minimum distance 0.11 ± 0.02; final distance 0.17 ± 0.07; arbitrary units normalized to maximum pairwise distance in PCA space).

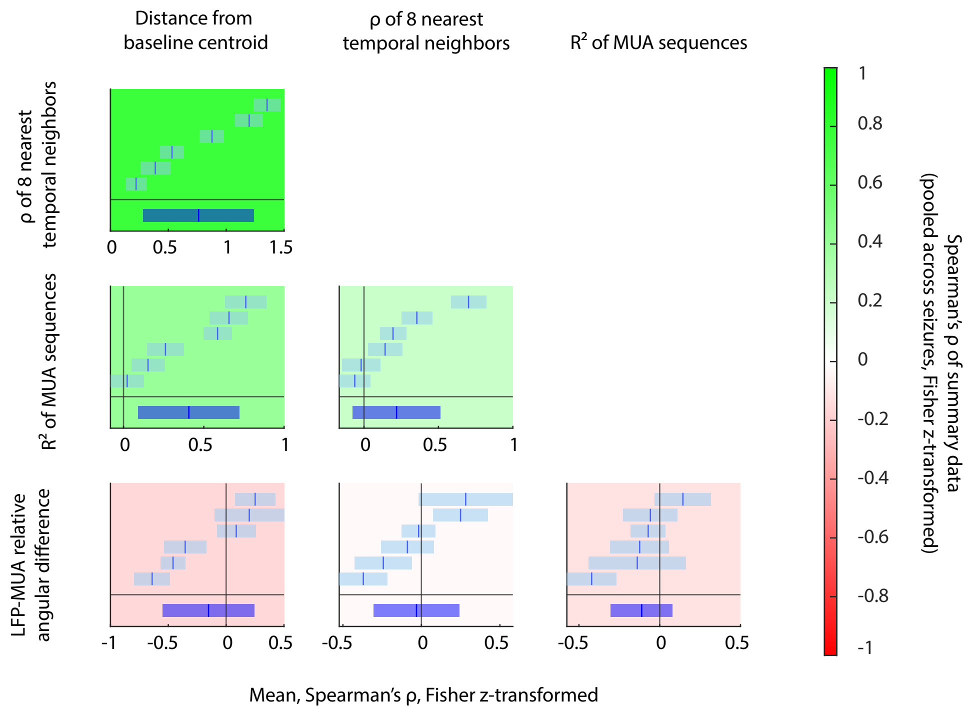

Supplementary Figure 9 Correlation of all measures to one another.

We compared the following measures to each other: distance of seizure bursts to the baseline centroid; Spearman's ρ between seizure bursts and 8 nearest temporal neighbors (a time-evolving measure of consistency); R^2 of seizure bursts (a measure of goodness of fit to spatial linear regression, indicating the directional nature of sequences); and the angular difference between MUA and LFP discharge directions. We pooled mean and standard deviation correlation values (Fisher z-transformed Spearman's ρ) across seizures, so that each data point represents a single array (*dark blue* vertical lines; *light blue* error bar indicates pooled 95% confidence interval; see *Methods*). Summary data across arrays is indicated by the *dark blue* vertical line and error bar (95% confidence interval) at the bottom of each comparison. The color of each panel is determined by the pooled value of ρ (*dark blue* vertical line below horizontal axis; see *colorbar*). Correlation between distance to baseline centroid and ρ of 8 nearest neighbors: $0.76 \pm 0.46; t(5) = 4.09, p = .009$, one-sample t-test. Correlation between distance to baseline centroid and R^2: $0.41 \pm 0.30; t(5) = 3.31, p = .021$, one-sample t-test. Correlation between distance to baseline centroid and angular difference between MUA and LFP discharge directions: $-0.15 \pm 0.38; t(5) = 0.986, p = .37$, one-sample t-test. Correlation between ρ of 8 nearest neighbors and R^2: $0.22 \pm 0.28; t(5) = 1.91, p = .11$ one-sample t-test. Correlation between ρ of 8 nearest neighbors and angular difference between MUA and LFP discharge directions: $-0.03 \pm 0.26; t(5) = 0.294, p = .78$, one-sample t-test. Correlation between R^2 and angular difference between MUA and LFP discharge directions: $-0.11 \pm 0.18; t(5) = 1.5, p = .19$, one-sample t-test.

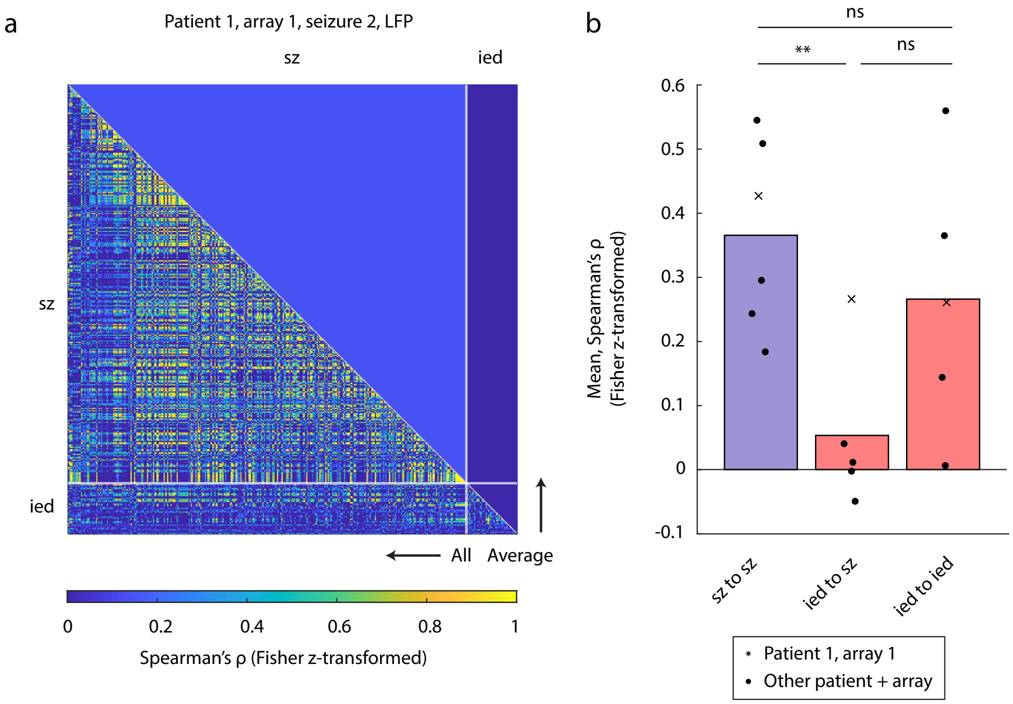

Supplementary Figure 10 Consistency of sequences for LFP discharges.

In Figure 5b and c, we noted that R^2 values for LFP were significantly greater than those for MUA. One potential reason for this would simply be that MUA signals are noisier than LFP signals. If this were true, we would expect LFP to have higher R^2 and also higher internal consistency according to Spearman's ρ. On the other hand, if internal consistency in LFP were similar to that in MUA, but R^2 was uniquely was higher in LFP, this would suggest that the high R^2 values in LFP sequences did not simply stem from reduced noise in these sequences. By the same token, it would suggest that MUA sequences were consistent but relatively *non*-directional. **a)** We identified the negative peak of the LFP discharge in every micro-electrode during every seizure burst, and extracted the temporal order, or sequence, of those discharges across electrodes. LFP discharge sequences were compared for all detected seizure and IED bursts in the same example participant and seizure illustrated in the main manuscript (*lower* triangle; average of all comparisons in *upper* triangle). Note that there are no baseline bursts shown here, because by definition, LFP discharges are not detected in the baseline bursts. For this seizure, there is weak similarity between seizure LFP sequences to one another ($\rho = 0.15 \pm 0.13$) and no similarity between seizure and IED LFP sequences ($\rho = 0.00 \pm 0.05$) or among IED LFP sequences ($\rho = 0.01 \pm 0.07$). **b)** Average similarity within and between seizure and IED LFP discharge sequences in all participants, averaged in each participant over all seizures (seizure to seizure $\rho = 0.37 \pm 0.15$; seizure to IED $\rho = 0.05 \pm 0.12$; IED to IED $\rho = 0.27 \pm 0.21$). Each point represents one participant and array ($n = 6$ arrays). There was a significant difference between at least two groups ($F(2, 8) = 9.061$, partial $\eta^2 = 0.70$, $p = .009$, repeated measures one-way ANOVA). A post-hoc test revealed a significant difference between the seizure to seizure comparison

and the seizure to IED comparison ($p = .009$, Bonferroni-Holm correction for multiple comparisons), but no significant differences otherwise. Interestingly, the values of ρ resulting from comparison of the LFP discharge sequences are similar to those seen in the MUA (Figure 1). These findings suggests that the reduced R^2 in MUA compared to LFP (Figure 5c) is not merely attributable to noise or inconsistency in MUA. Rather, sequence consistency is preserved in the MUA with respect to the LFP, but the directional nature of the MUA is decreased. This may suggest that LFP is the fundamental *source* of entrainment, as its sequences are strongly directional. The MUA, on the other hand, is only partially entrained. Some features of the native (non-directional) neural network may be preserved in seizure MUA sequences, even in the setting of emergence of (partial) directional properties in these sequences.

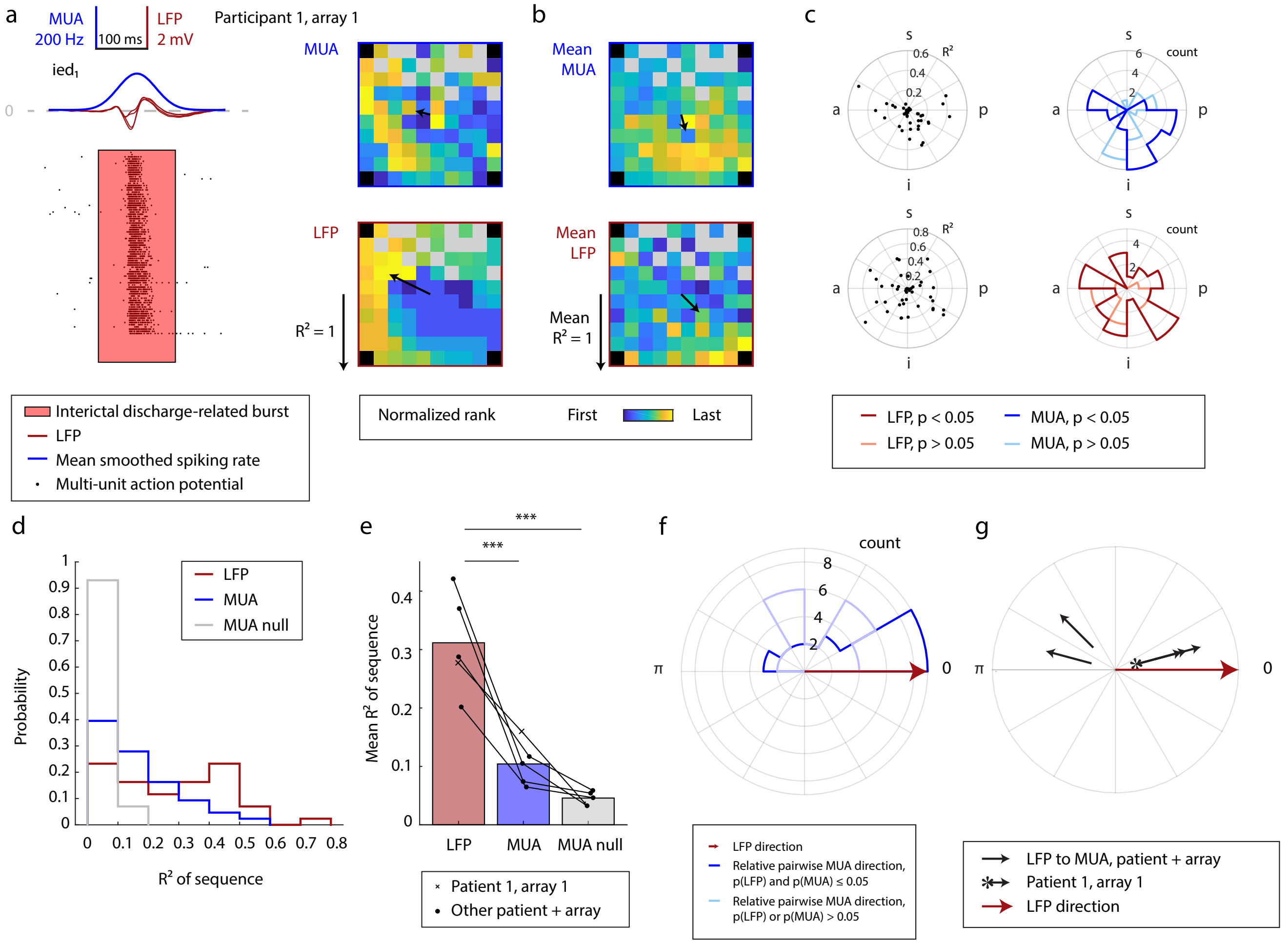

Supplementary Figure 11 Directional properties of IED sequences.

a) *Left*: a single representative IED burst from Figure 1 is reproduced here. *Dark blue* trace indicates mean smoothed MUA across electrodes, and *maroon* traces indicate LFP from four randomly-chosen electrodes. Robust multi-unit activity (*black dots*) coincides with a sharp deflection in the LFP. *Right*: map of the timing of peak multi-unit spiking activity (MUA; *top*) and LFP discharges (*bottom*) on the spatial layout of the MEA during the same example IED burst (*blue*: early MUA; *yellow*: late MUA). We fit a plane to the spatial organization of each spiking sequence and sequence of LFP discharges, generating a measure of the goodness of fit, R^2, and a direction (indicated by the *size* and *direction* of the *black arrow*; $R^2 = 0.13$ for the MUA; $R^2 = 0.56$ for the LFP). **b)** Average temporal sequence of MUA (*top*) and LFP IED discharges (*bottom*) mapped to the spatial layout of MEA. Average R^2 and most common direction (mode of polar histograms with significant spatial fit in **c**, *right* panel) are indicated by length and direction, respectively, of *black arrow*. Average sequences appear disorganized, because of inconsistency in the direction of individual sequences. **c)** *Left:* Direction and R^2 for best fit of spatial linear regression of MUA sequences (*top*) and LFP discharges (*bottom*) for every IED burst in this example participant. *Right:* Distribution of directions generated from spatial fits of MUA sequences (*top*) and LFP discharges (*bottom*) during every IED burst in

this example participant. Distribution of directions for bursts with significant fits ($p \leq .05$) are indicated by darker colors, while bursts that did not have significant fit are indicated by lighter colors. For both MUA and LFP, burst directions are variable, with no clear central tendency of the distributions. **d)** Distribution of R^2 values for all MUA spiking sequences during IED bursts and sequences of LFP discharges in this example participant, compared to null bursts in which the sequences of MUA activity are shuffled (see *Methods*). For LFP, $R^2 = 0.28 \pm 0.19$; for MUA, $R^2 = 0.16 \pm 0.14$; and for null, $R^2 = 0.04 \pm 0.04$. **e)** Average R^2 over all IED bursts for LFP discharges, spiking sequences, and shuffled bursts (null) in every participant and array. There are only five data points because one participant had no IED-related bursts (Supplementary Table 3). Here, for LFP, $R^2 = 0.31 \pm 0.09$; for MUA, $R^2 = 0.10 \pm 0.04$; and for null, $R^2 = 0.05 \pm 0.01$. There was a significant influence of burst feature (LFP vs MUA vs null) on R^2 ($F(2, 8) = 31.95$, partial $\eta^2 = 0.89$, $p < .001$, repeated measures one-way ANOVA). A post-hoc test revealed a significant difference between IED LFP R^2 and IED MUA R^2 ($p < .001$) and between IED LFP R^2 and IED MUA R^2 ($p < .001$) but not between IED MUA and null ($p = .13$, all p-values adjusted using the Bonferroni-Holm correction for multiple comparisons). Therefore, a similar trend to that in the seizure bursts is seen, where directionality is greatest in LFP and least in null. However, here, the difference between MUA and null is not statistically-significant. This may reflect decreased entrainment of MUA spiking sequences in IEDs compared to seizures (see Figure 4c). **f)** Distribution of angular differences between spatial fit of spiking sequences and LFP discharges across all IED bursts in this example participant. The distribution of differences for bursts with significant spatial fits (spatial linear regression gives $p \leq .05$) is shown in *dark blue*. Angular differences are absolute values with respect to the direction of the LFP discharges, indicated by the *maroon arrow* fixed at $\theta = 0$. For most IED bursts with significant spatial fit in this participant, MUA and LFP sequences have similar direction. This is based on the fact that the mode of the *dark blue* histogram resides between 0 and $\pi/6$. **g)** The mode of the distribution of angular differences between the direction of MUA and LFP discharge sequences that had a significant spatial fit, taken over all IED bursts separately in each array. *Black arrows* indicate the mode for each participant and array, and the distance from the origin (along the radial axis) is jittered to facilitate visualization. The angular difference between LFP discharges and MUA is variable, suggesting that during IEDs, LFP discharges do not necessarily entrain the spatial organization of the MUA sequences.

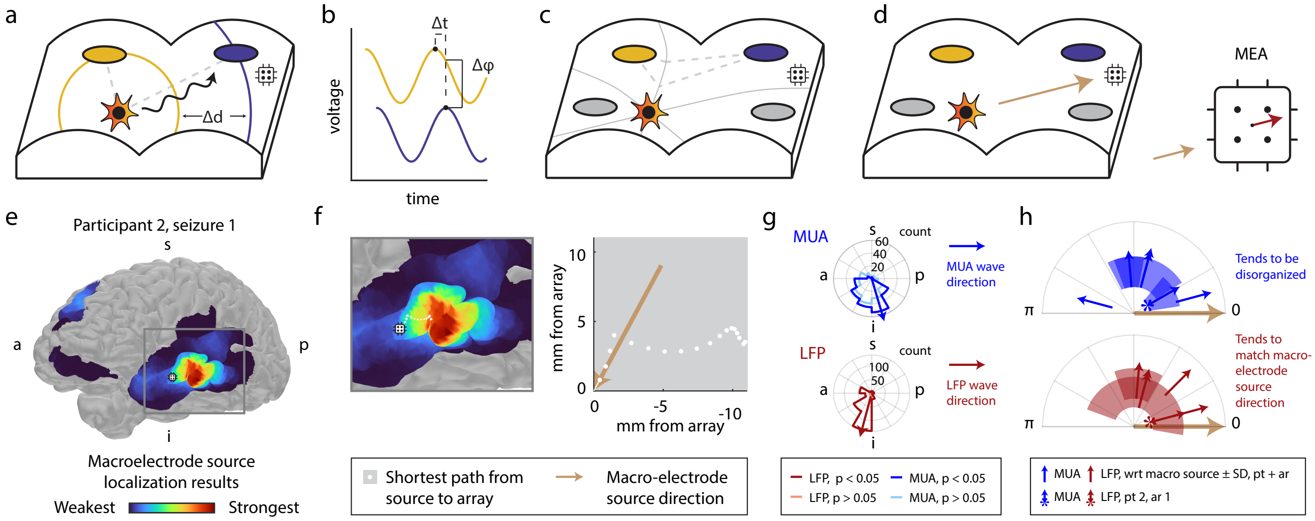

Supplementary Figure 12 LFP directionality matches the direction of incoming traveling waves from the putative seizure source

Our analyses in the main manuscript suggest that traveling waves in the LFP influence directionality of the MUA. We hypothesized that these LFP traveling waves arose directly from the seizure source, in light of previous work, which has suggested that the seizure source releases traveling waves which travel over macroscopic distances in the LFP[3–8]. We wished to validate our hypothesis by confirming that the direction of the LFP recorded on the micro-electrode array did in fact match the direction of incoming traveling waves from the seizure source.

a) We estimated seizure source location from macro-electrode recordings using a previously-described approach[5,7]. This approach assumes that there is a focal source of seizure activity (*star*) that emits traveling waves (*black*), which spread outward over the cortical surface in a radial fashion (*yellow* and *blue* curves represent wave crests). **b)** When viewed as a time series, we can see that the yellow trace crests sooner, as it is closer to the source. We use the difference in phase of the waves recorded at adjacent electrodes as a surrogate for the difference in time, in order to compute the relative difference in distance between the source and each electrode. **c)** The relative difference in distance (*grey* dashed lines) between the source and two electrodes, computed using the difference in phases of the recorded oscillatory signal, constrains the location of the source of activity to a hyperbola (*grey* solid line). With multiple pairs of electrodes, we produce multiple hyperbolae at each time point of seizure activity. We estimate the location of the source (*star*) as the point that lies closest to the intersection of the hyperbolae. **d)** We then can take the shortest path (*tan arrow*) between the putative source (*star*) and the micro-electrode array. *Inset:* Based on previous work showing that waves travel over the gray matter in the local field potential[3–8], we hypothesized that the LFP (*red arrow*), but not necessarily the MUA, would arrive from the same direction as the putative seizure source, as determined by macro-electrode recordings. **e)** Source localization results for participant 2, array 1, seizure 1 (left lateral view). The source lies in the middle temporal gyrus, about 15 mm posterior to the MEA. **f)** We took the shortest path (*white dots*) from the seizure source to the

micro-electrode array (*left* panel). We then took the line between the point on the shortest path closest to the array and the array itself. This line was projected onto the sagittal plane, and its angle was taken (*right* panel, *tan arrow*). The wire bundle of the array was taken to point superiorly, based on the fact that the neurosurgeon at our center (K.A.Z.) places the wire bundle superiorly in all patients. This was confirmed from reviewing intra-operative photos. **g)** Polar histograms of MUA (*top*) and LFP (*bottom*) sequence directionality for participant 2, array 1, seizure 1. Distribution of directions for sequences with significant fit to the plane ($p \leq .05$) are shown in darker colors, while sequences that did not have significant fit are shown in lighter colors. The mode LFP direction, mostly inferior and slightly anterior (*red arrow*) is virtually identical to the macro-electrode source direction (*tan arrow* in **f**). The MUA source direction, on the other hand, is not as similar (*dark blue arrow*). **h)** Absolute value of direction of MUA (*top*) and LFP (*bottom*) was taken for all participants and arrays, normalized to the direction of incoming waves from the putative macro-electrode array source (*tan arrow*, fixed at $\theta = 0$). Here, each *maroon* or *dark blue* arrow reflects the mean, across all seizures, for a single array. *Shaded error bars* reflect standard deviation of each array. For each array, distance from the origin (along the radial axis) is jittered to facilitate visualization. The relative direction of the LFP (*maroon arrows*) tends to reside between 0 and $\pi/2$, suggesting that the direction is similar to that of the incoming waves from the putative macro-electrode source. This trend is not seen in the MUA (*dark blue arrows.*) Supplementary Figure 12 panels **a-e** were adapted from Diamond, J. M. *et al.* Travelling waves reveal a dynamic seizure source in human focal epilepsy. *Brain* **144**, 1751–1763 (2021).

Here, the analyses shown in the figures in the main manuscript and supplement are repeated for each patient and seizure. Raw output figures are provided, as described in the table of contents below.

Table of Contents

Patient 1, array 1 **1**

Baseline activity 1

IED activity 3

Seizure activity, seizure 1 5

Seizure activity, seizure 2 11

Seizure activity, seizure 3 17

Seizure activity, seizure 4 23

Seizure activity, seizure 5 29

Seizure activity, seizure 6 35

Patient 2, array 1 **41**

Baseline activity 41

IED activity 43

Seizure activity, seizure 1 45

Seizure activity, seizure 2 51

Patient 2, array 2 **57**

Baseline activity 57

IED activity 59

Seizure activity, seizure 1 61

Seizure activity, seizure 2 67

Patient 3, array 1 **73**

Baseline activity 73

IED activity 75

Seizure activity, seizure 1 77

Patient 4, array 1 **83**

Baseline activity 83

IED activity 85

Seizure activity, seizure 1 87

Seizure activity, seizure 2 93

Patient 5, array 1 **99**

Baseline activity 99

IED activity 101

Seizure activity, seizure 5 101

Seizure activity, seizure 6 107

Patient 1, array 1

Baseline activity

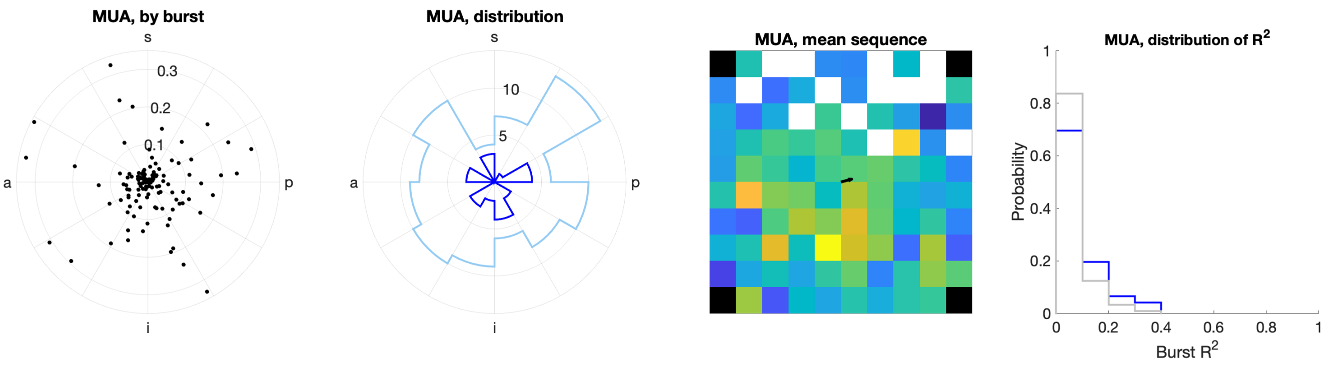

Supplementary Figure 13 Patient 1, array 1, baseline wavelike properties. First panel from left: each dot represents a burst. θ reflects direction, and ρ is R^2, a measure of goodness of fit of the data to a plane. In other words, it is a measure of the 'wavelike-ness' of the burst. Second panel from left: distribution direction of all recorded bursts. *Dark* colors indicate bursts with spatial linear regression giving $p \leq 0.05$, while *light* colors indicate bursts with $p > 0.05$. Third panel from left: the average (*backbone*) sequence is shown over all bursts. This is obtained by taking the mean normalized rank of all sequences. The length of the *black arrow* indicates the mean R^2 over all bursts of this type, while the angle of the arrow indicates the mode of the histogram in the *second from left* panel, for bursts with $p \leq 0.05$. Fourth panel from left: R^2 for all bursts is shown. *Blue* histogram indicates the true sequences. *Gray* histogram, on the other hand, indicates R^2 for a set of null sequences, created by shuffling the ranks of the true sequences.

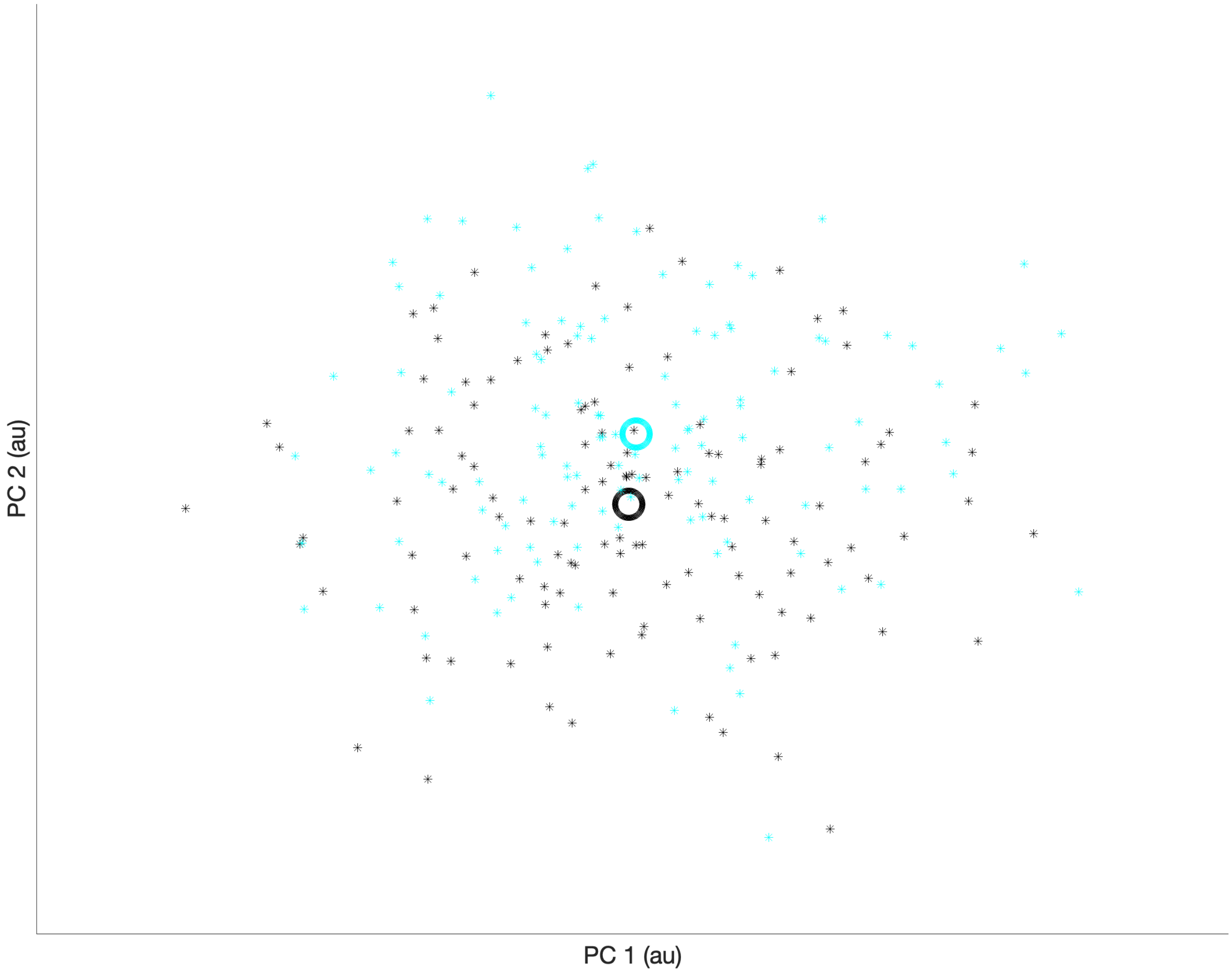

Supplementary Figure 14 Patient 1, array 1, baseline bursts versus random bursts. We were interested in determining that our baseline bursts were non-random. Therefore, for each baseline burst, we created a null counterpart, in which the MUA timings were shuffled only among spiking electrodes. We then applied both the true baselines and the random counterparts to the same dimensionality reduction procedure. The baseline centroid is indicated by the *blue circle*. The random centroid indicated by the *black* circle. For most patients and arrays, baseline bursts are displaced from the random bursts. Distance from baseline bursts to the random centroid tends to be greater than distance from random bursts to the random centroid (Supplementary Figure 4).

IED activity

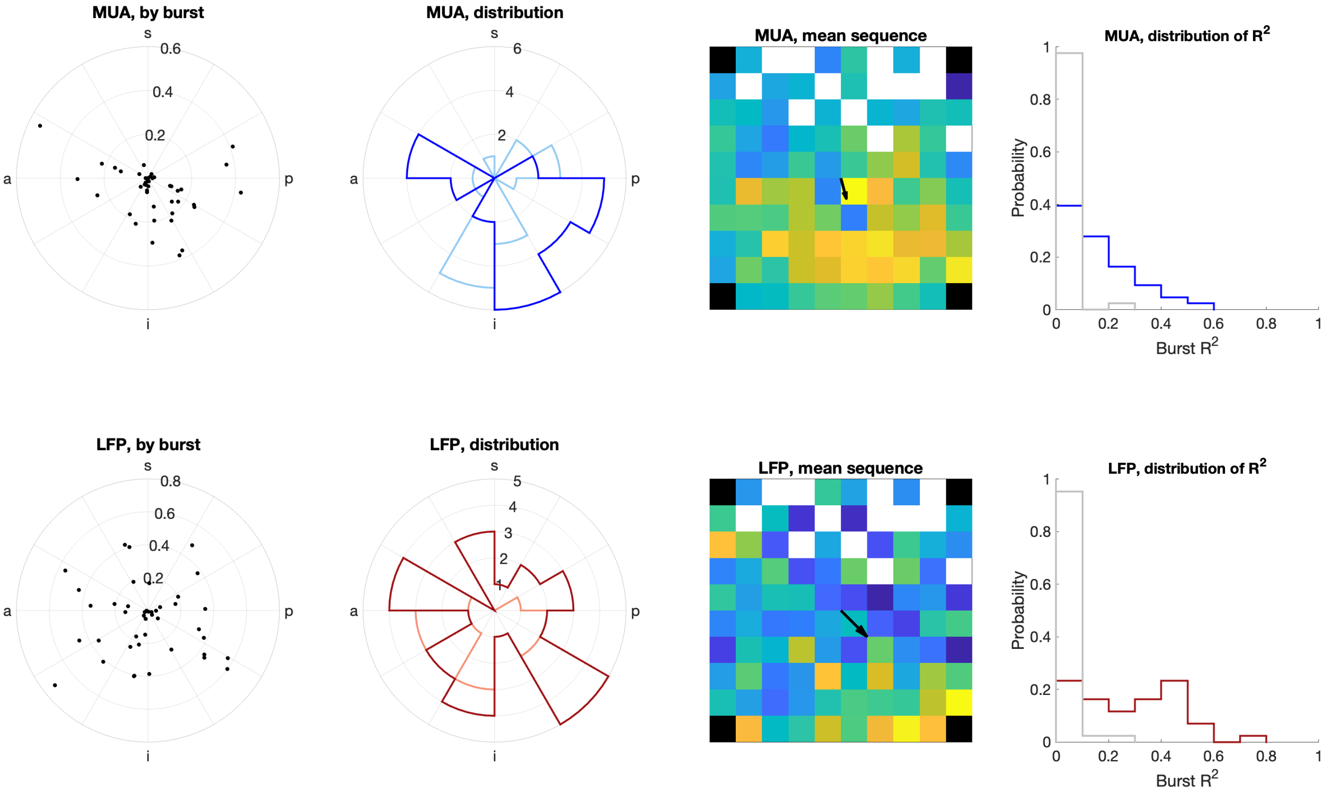

Supplementary Figure 15 Patient 1, array 1, IED directionality. Top row: LFP. Bottom row: MUA. *Dark blue* histograms are used to designated MUA, while *maroon* histograms are used to designate LFP. First column from left: each dot represents a burst. θ reflects direction, and ρ is R^2, a measure of goodness of fit of the data to a plane. In other words, it is a measure of the strength of directionality of the burst. Second column from left: distribution direction of all recorded bursts. *Dark colors* indicate bursts with spatial linear regression giving $p \leq 0.05$, while *light* colors indicate bursts with $p > 0.05$. Third column from left: the average (*backbone*) sequence is shown over all bursts for this seizure. This is obtained by taking the mean normalized rank of all sequences. The length of the *black arrow* indicates the mean R^2 over all bursts of this type, while the angle of the arrow indicates the mode of the histogram in the *second from left* column, for bursts with $p \leq 0.05$. Fourth column from left: R^2 for all bursts is shown. *Colored* histogram indicates the true sequences. *Gray* histogram, on the other hand, indicates R^2 for a set of null sequences, created by shuffling the ranks of spiking electrodes in the true sequences.

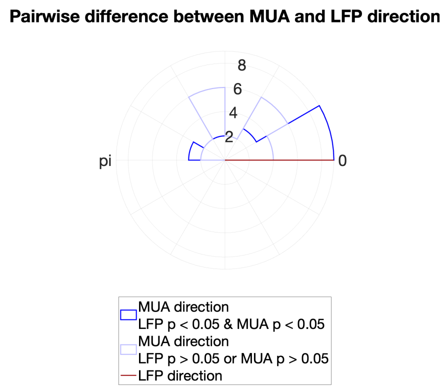

Supplementary Figure 16 Patient 1, array 1, IED relationship between MUA and LFP direction. Distribution of pairwise LFP-to-MUA direction. For each burst, LFP and MUA direction are compared, and the absolute value of the angular difference is put into the histogram. Bursts are divided into those with LFP and MUA p-value both less than 0.05, or those with either MUA or LFP with p-value greater than 0.05.

Seizure activity, seizure 1

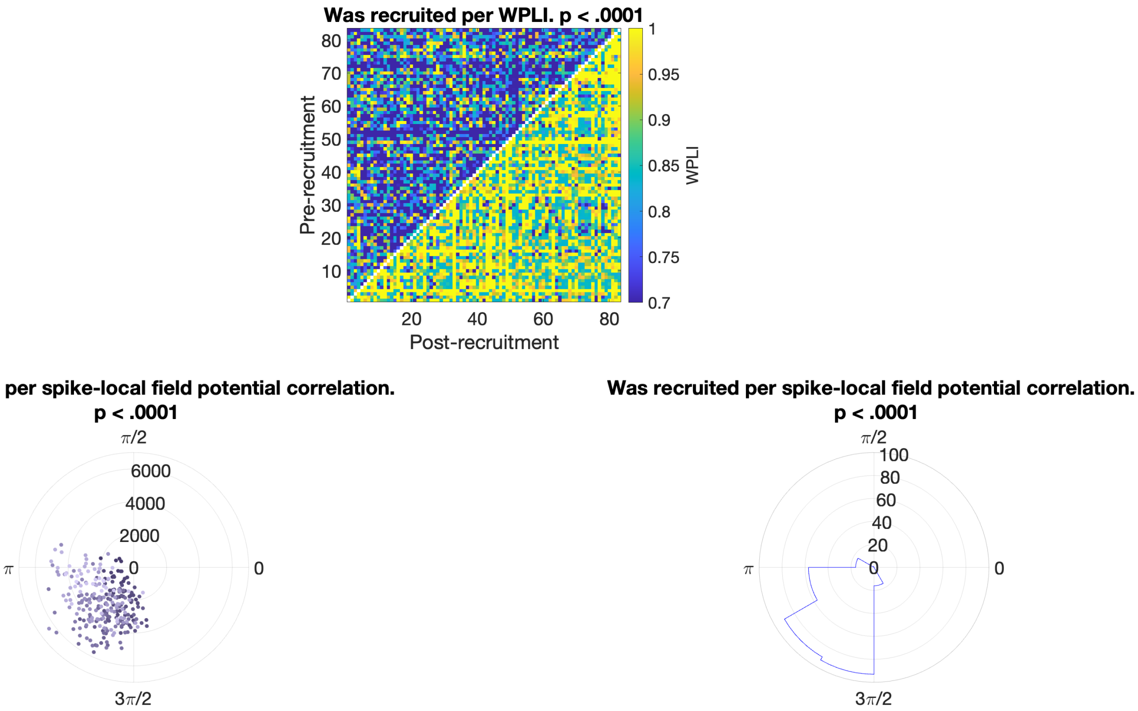

Supplementary Figure 17 Measures of recruitment for patient 1, array 1, seizure 1. Top: weighted phase lag index (WPLI), 10 seconds prior to seizure onset (top left), and 10 seconds after recruitment (bottom right, see *Methods*). Bottom left: relationship between LFP phase (θ) and LFP phase power (ρ), over the course of the seizure. *Dark purple* dots: early seizure; *light purple* dots: late seizure. Bottom right: distribution of spike-local field correlation values.

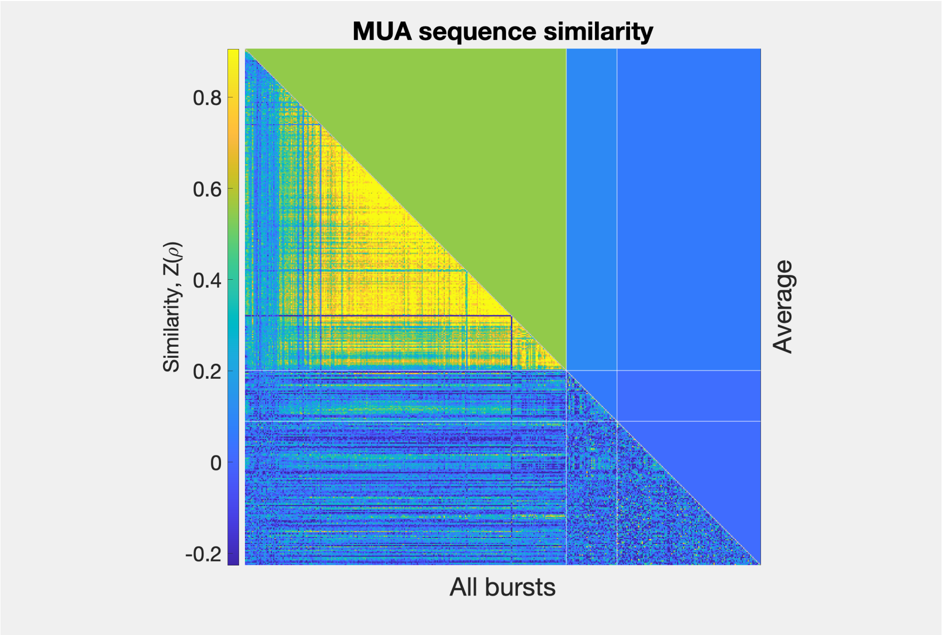

Supplementary Figure 18 Patient 1, array 1, seizure 1, burst similarity. For this patient, array, and seizure, every sequence was compared to every other sequence. Each cell indicates the similarity of the row sequence to the column sequence (Spearman's ρ). Sequences are divided by *white* lines into seizure sequences (*left, top*), IED sequences (*middle*), and baseline sequences (*right, bottom*). Actual values are provided in the lower triangle. In the upper triangle, average values are provided for each group, with values represented by the same color axis.

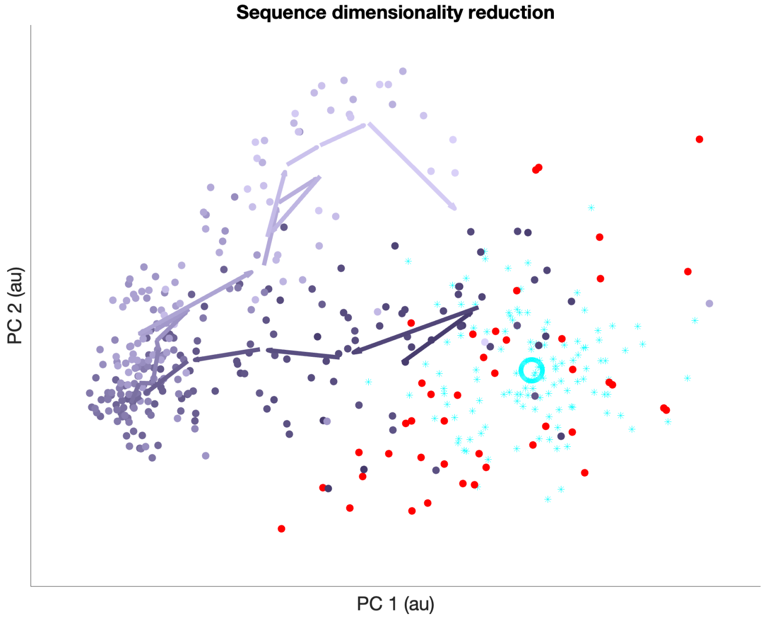

Supplementary Figure 19 Patient 1, array 1, seizure 1, dimensionality reduction using principal component analysis (PCA) was performed. We used PCA to decompose high-dimensional data into two dimensions in an unsupervised fashion (see *Methods*). Data provided include baseline bursts (*cyan stars*), IED bursts (*red dots*), and seizure bursts (*colored dots*; *dark purple*, early seizure, *light purple*, late seizure). The baseline centroid is indicated by the *cyan circle*.

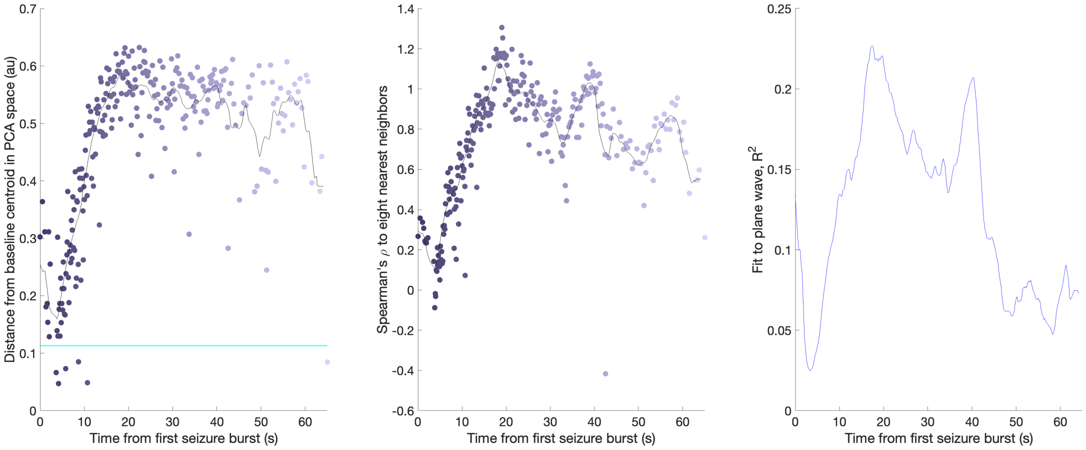

Supplementary Figure 20 Patient 1, array 1, seizure 1, measures of wave entrainment over time. *Left*: We used dimensionality reduction to collapse high-dimensional sequence information into two dimensions (see Figure 2a). We then took the location of the baseline centroid, and captured the distance, over time, between seizure bursts and the baseline centroid, in the low-dimensional manifold (see Figure 2c). *Dark purple* dots: early seizure; *light purple* dots: late seizure. A three-second moving average is superimposed (*black line*). Mean distance of baseline bursts to the baseline centroid is indicated by the *cyan line*. *Middle*: we captured Spearman's ρ between each seizure burst and its eight nearest temporal neighbors (see Figure 3a, b). This can be thought of as a measure of consistency of seizure bursts to each other in time. A three-second moving average of the ρ values is shown (*black line*). *Right*: for each burst, the R^2 value was determined from spatial linear regression (see *Methods*, section **Directionality of spike bursts and LFP discharges**). R^2 is a measure of the extent to which a burst is directional. A three-second moving average of the R^2 values is shown (*blue line*).

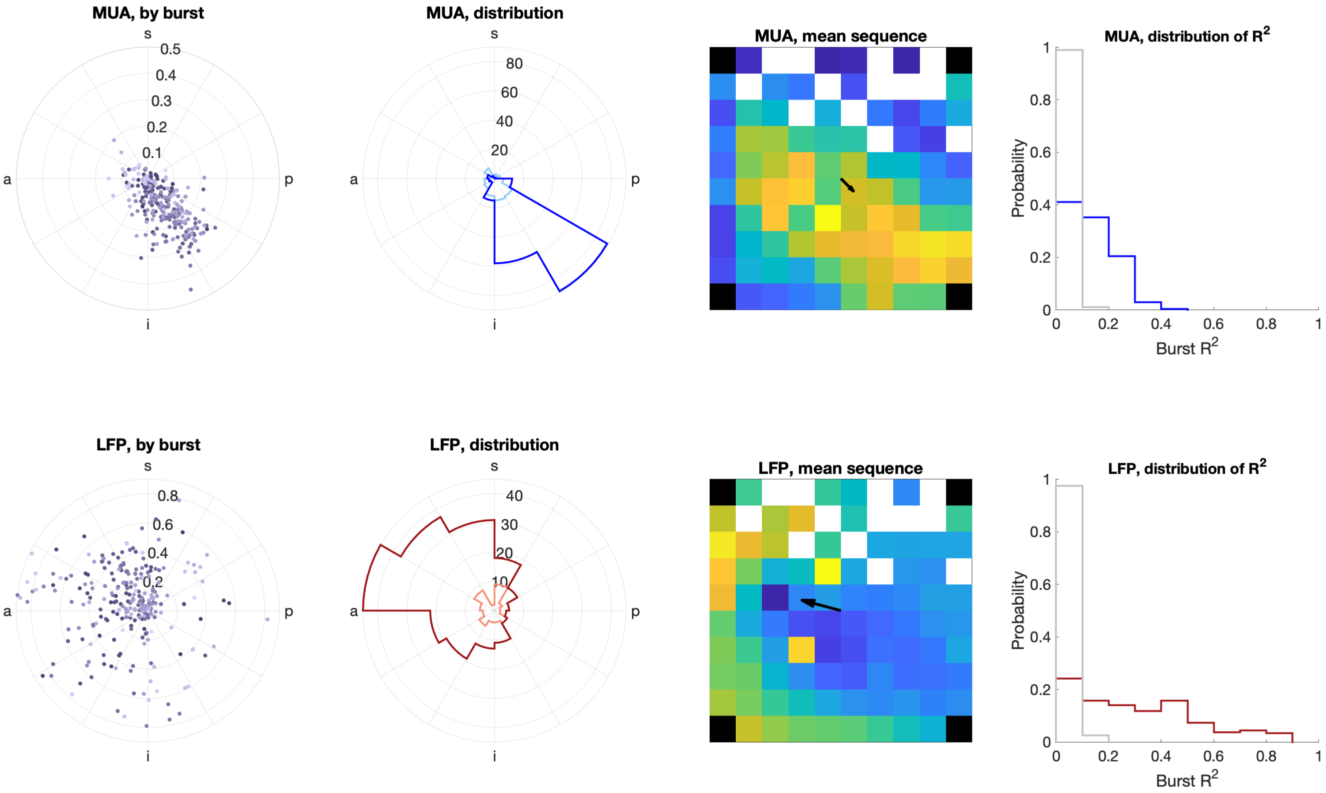

Supplementary Figure 21 Patient 1, array 1, seizure 1, seizure directionality. Top row: LFP. Bottom row: MUA. *Blue* histograms are used to designated MUA, while *maroon* histograms are used to designate LFP. First column from left: each dot represents a burst. θ reflects direction, and ρ is R^2, a measure of goodness of fit of the data to a plane. In other words, it is a measure of directionality of the burst. Second column from left: distribution direction of all recorded bursts. *Dark colors* indicate bursts with spatial linear regression giving $p \leq 0.05$, while *light* colors indicate bursts with $p > 0.05$. Third column from left: the average (*backbone*) sequence is shown over all bursts. This is obtained by taking the mean normalized rank of all sequences. The length of the *black arrow* indicates the mean R^2 over all bursts of this type, while the angle of the arrow indicates the mode of the histogram in the *second from left* column, for bursts with $p \leq 0.05$. Fourth column from left: R^2 for all bursts is shown. *Colored* histogram indicates the true sequences. *Gray* histogram, on the other hand, indicates R^2 for a set of null sequences, created by shuffling the ranks of the true sequences.

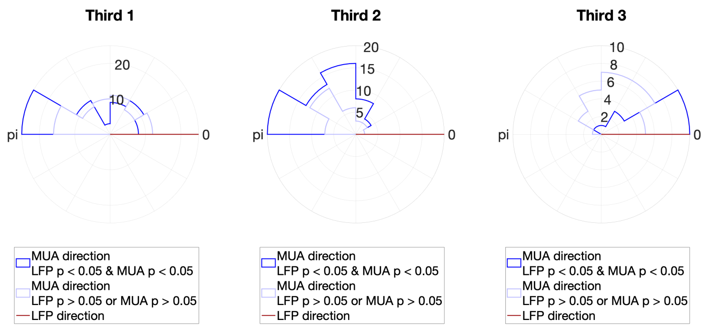

Supplementary Figure 22 Patient 1, array 1, seizure 1, relationship between MUA and LFP direction. Seizure bursts were divided into thirds (*left*: first third; *middle*: middle third, *right*: last third). For each third, we provided the distribution of burst-wise LFP-to-MUA directions. For each burst, LFP and MUA direction are compared, and the absolute value of the angular difference is put into the histogram. Bursts are divided into those with LFP and MUA p-value both less than 0.05 (*dark blue* histogram), and those with either MUA or LFP with p-value greater than 0.05. (*light blue* histogram).

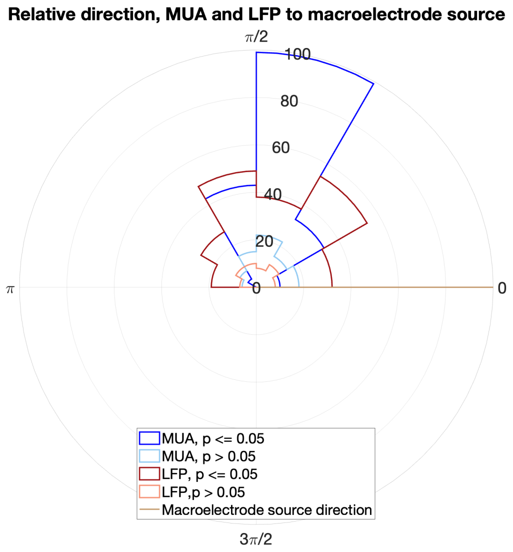

Supplementary Figure 23 Patient 1, array 1, seizure 1, relationship between discharge source, as determined by macroelectrode recordings, and direction of burst MUA and LFP. We used the time differences of the signal recorded in macroelectrodes to determine the location of the discharge source (Diamond, et al 2021, Diamond, et al 2023, see Supplementary Figure 12). The shortest path from the source to the array was then obtained, and we measured the angle of approach of the shortest path to the array (*tan* line). We then determined the relative direction of MUA and LFP signal, for each burst, to the discharge source as measured by macroelectrodes. Histograms are then provided, for the absolute value of the relative direction between source and MUA sequences (*blue*) and source and LFP sequences (*red*), for significant sequences (*dark* colors) and non-significant sequences (*light* colors).

Seizure activity, seizure 2

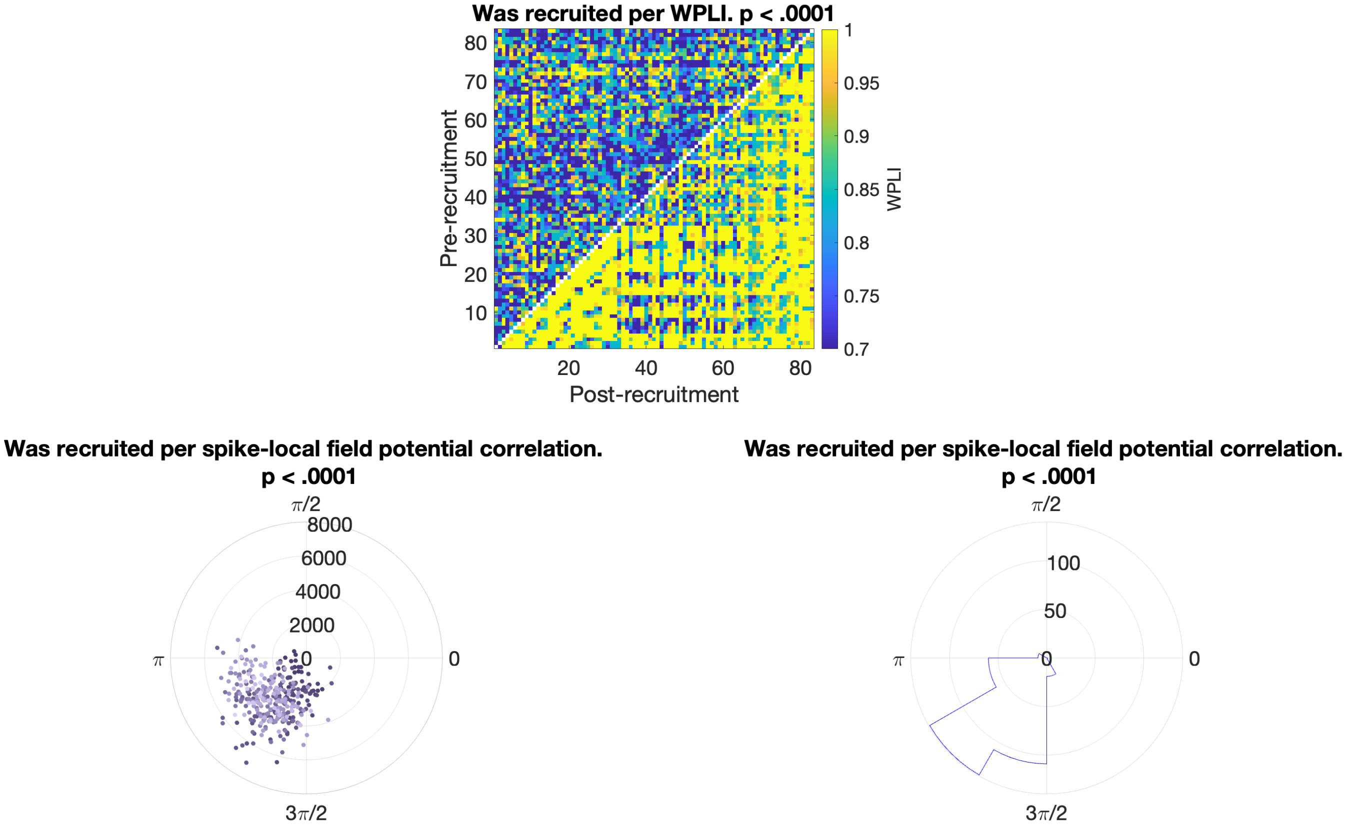

Supplementary Figure 24 Measures of recruitment for patient 1, array 1, seizure 2. Top: weighted phase lag index (WPLI), 10 seconds prior to seizure onset (top left), and 10 seconds after recruitment (bottom right, see *Methods*). Bottom left: relationship between LFP phase (θ) and LFP phase power (ρ), over the course of the seizure. *Dark purple* dots: early seizure; *light purple* dots: late seizure. Bottom right: distribution of spike-local field correlation values.

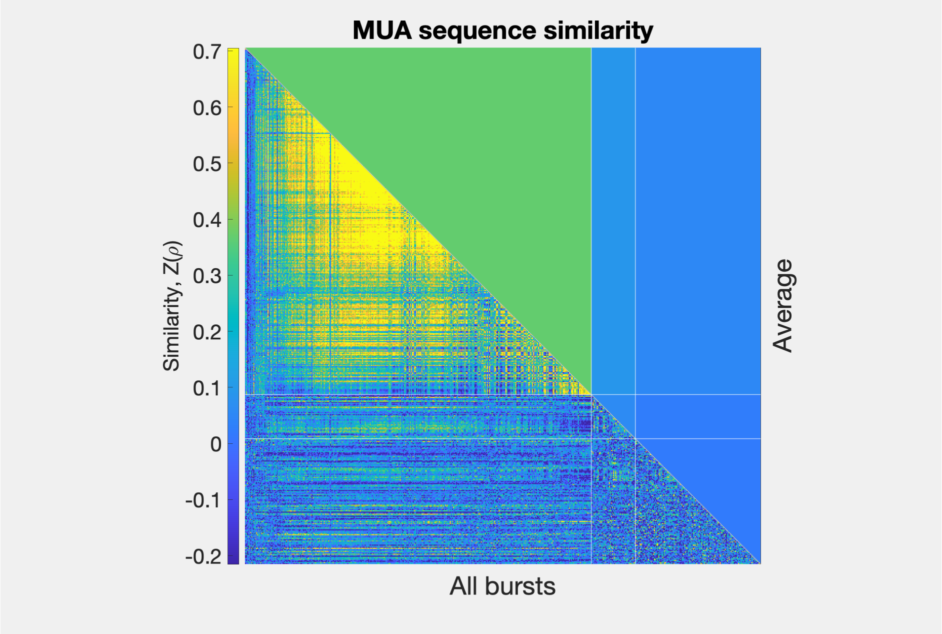

Supplementary Figure 25 Patient 1, array 1, seizure 2, burst similarity. For this patient, array, and seizure, every sequence was compared to every other sequence. Each cell indicates the similarity of the row sequence to the column sequence (Spearman's ρ). Sequences are divided by *white* lines into seizure sequences (*left, top*), IED sequences (*middle*), and baseline sequences (*right, bottom*). Actual values are provided in the lower triangle. In the upper triangle, average values are provided for each group, with values represented by the same color axis.

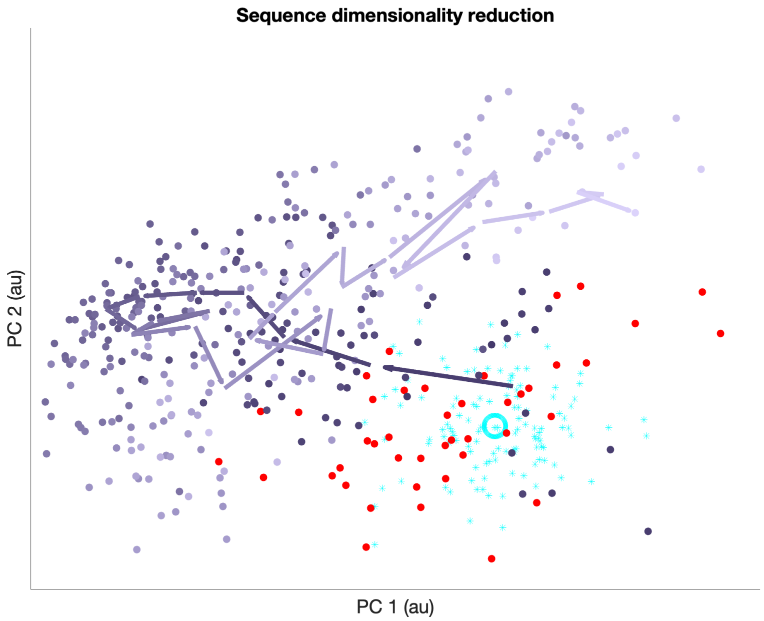

Supplementary Figure 26 Patient 1, array 1, seizure 2, dimensionality reduction using principal component analysis (PCA) was performed. We used PCA to decompose high-dimensional data into two dimensions in an unsupervised fashion (see *Methods*). Data provided include baseline bursts (*cyan stars*), IED bursts (*red dots*), and seizure bursts (*colored dots*; *dark purple*, early seizure, *light purple*, late seizure). The baseline centroid is indicated by the *cyan circle*.

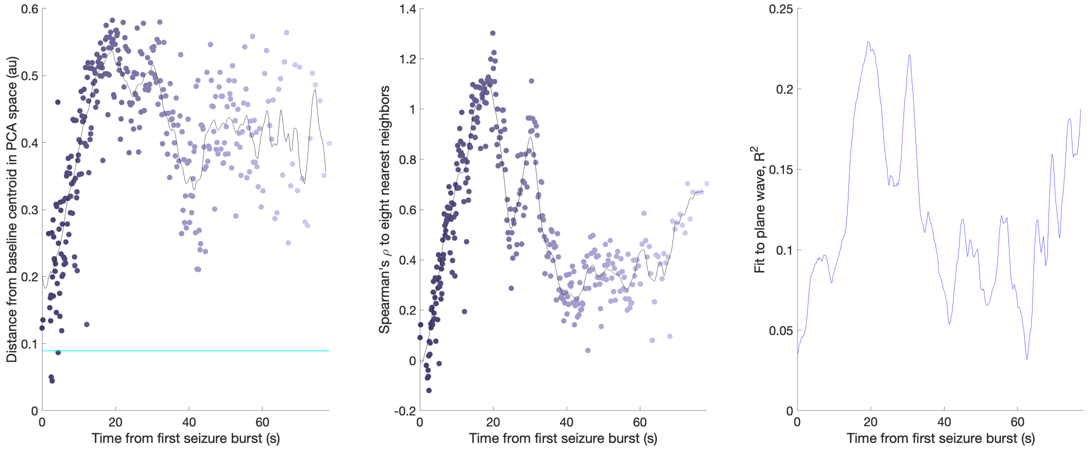

Supplementary Figure 27 Patient 1, array 1, seizure 2, measures of wave entrainment over time. *Left*: We used dimensionality reduction to collapse high-dimensional sequence information into two dimensions (see Figure 2a). We then took the location of the baseline centroid, and captured the distance, over time, between seizure bursts and the baseline centroid, in the low-dimensional manifold (see Figure 2c). *Dark purple* dots: early seizure; *light purple* dots: late seizure. A three-second moving average is superimposed (*black line*). Mean distance of baseline bursts to the baseline centroid is indicated by the *cyan line*. *Middle*: we captured Spearman's ρ between each seizure burst and its eight nearest temporal neighbors (see Figure 3a, b). This can be thought of as a measure of consistency of seizure bursts to each other in time. A three-second moving average of the ρ values is shown (*black line*). *Right*: for each burst, the R^2 value was determined from spatial linear regression (see *Methods*, section **Directionality of spike bursts and LFP discharges**). R^2 is a measure of the extent to which a burst is directional. A three-second moving average of the R^2 values is shown (*blue line*).

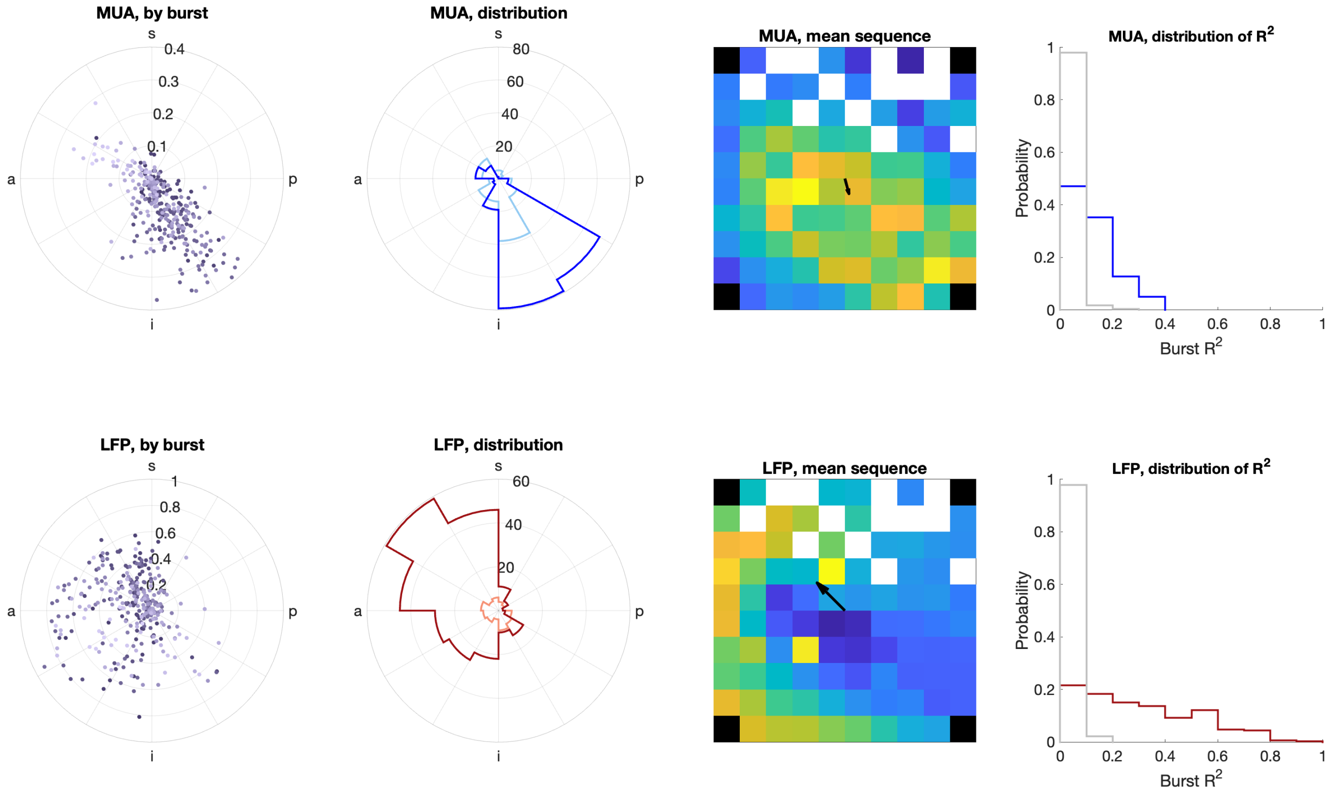

Supplementary Figure 28 Patient 1, array 1, seizure 2, seizure directionality. Top row: LFP. Bottom row: MUA. *Blue* histograms are used to designated MUA, while *maroon* histograms are used to designate LFP. First column from left: each dot represents a burst. θ reflects direction, and ρ is R^2, a measure of goodness of fit of the data to a plane. In other words, it is a measure of directionality of the burst. Second column from left: distribution direction of all recorded bursts. *Dark colors* indicate bursts with spatial linear regression giving $p \leq 0.05$, while *light* colors indicate bursts with $p > 0.05$. Third column from left: the average (*backbone*) sequence is shown over all bursts. This is obtained by taking the mean normalized rank of all sequences. The length of the *black arrow* indicates the mean R^2 over all bursts of this type, while the angle of the arrow indicates the mode of the histogram in the *second from left* column, for bursts with $p \leq 0.05$. Fourth column from left: R^2 for all bursts is shown. *Colored* histogram indicates the true sequences. *Gray* histogram, on the other hand, indicates R^2 for a set of null sequences, created by shuffling the ranks of the true sequences.

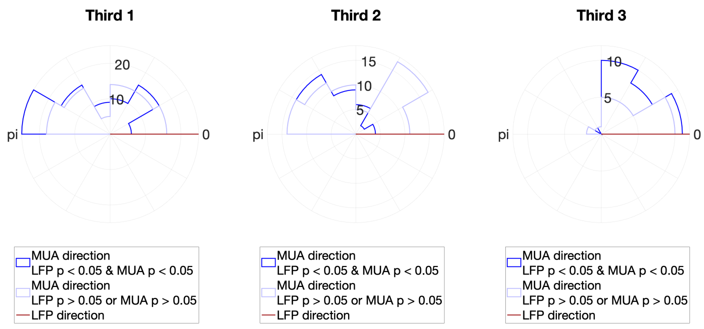

Supplementary Figure 29 Patient 1, array 1, seizure 2, relationship between MUA and LFP direction. Seizure bursts were divided into thirds (*left*: first third; *middle*: middle third, *right*: last third). For each third, we provided the distribution of burst-wise LFP-to-MUA directions. For each burst, LFP and MUA direction are compared, and the absolute value of the angular difference is put into the histogram. Bursts are divided into those with LFP and MUA p-value both less than 0.05 (*dark blue* histogram), and those with either MUA or LFP with p-value greater than 0.05. (*light blue* histogram).

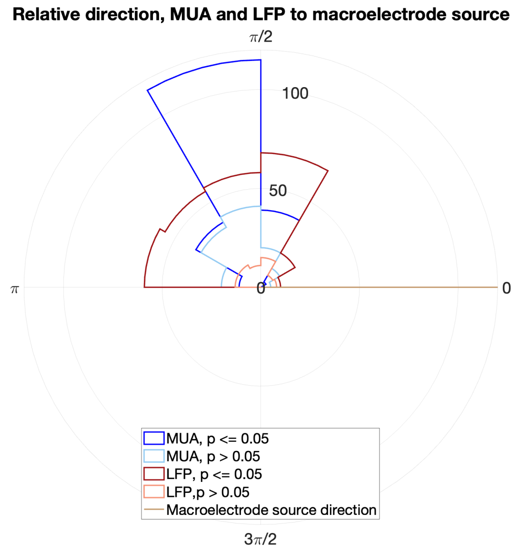

Supplementary Figure 30 Patient 1, array 1, seizure 2, relationship between discharge source, as determined by macroelectrode recordings, and direction of burst MUA and LFP. We used the time differences of the signal recorded in macroelectrodes to determine the location of the discharge source (Diamond, et al 2021, Diamond, et al 2023, see Supplementary Figure 12). The shortest path from the source to the array was then obtained, and we measured the angle of approach of the shortest path to the array (*tan* line). We then determined the relative direction of MUA and LFP signal, for each burst, to the discharge source as measured by macroelectrodes. Histograms are then provided, for the absolute value of the relative direction between source and MUA sequences (*blue*) and source and LFP sequences (*red*), for significant sequences (*dark* colors) and non-significant sequences (*light* colors).

Seizure activity, seizure 3

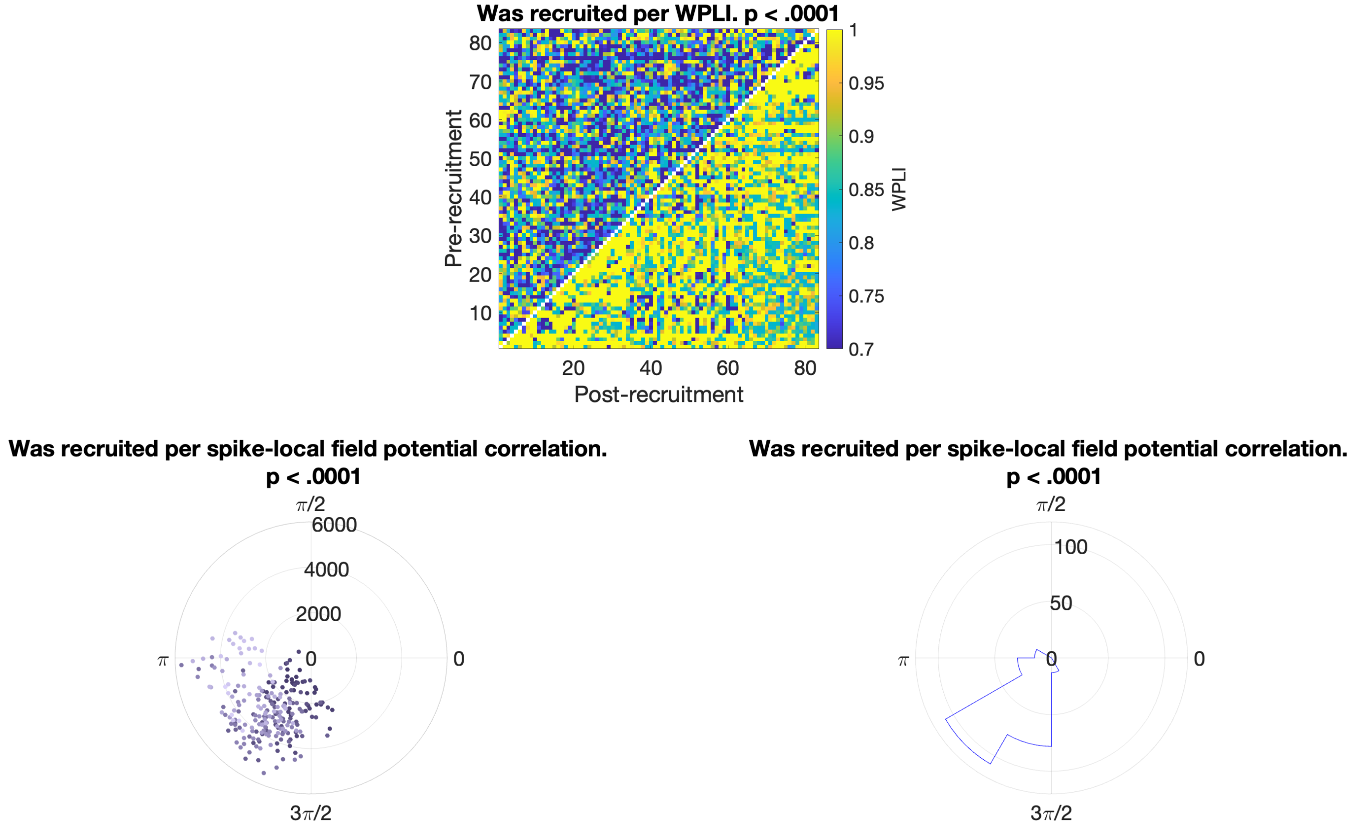

Supplementary Figure 31 Measures of recruitment for patient 1, array 1, seizure 3. Top: weighted phase lag index (WPLI), 10 seconds prior to seizure onset (top left), and 10 seconds after recruitment (bottom right, see *Methods*). Bottom left: relationship between LFP phase (θ) and LFP phase power (ρ), over the course of the seizure. *Dark purple* dots: early seizure; *light purple* dots: late seizure. Bottom right: distribution of spike-local field correlation values.

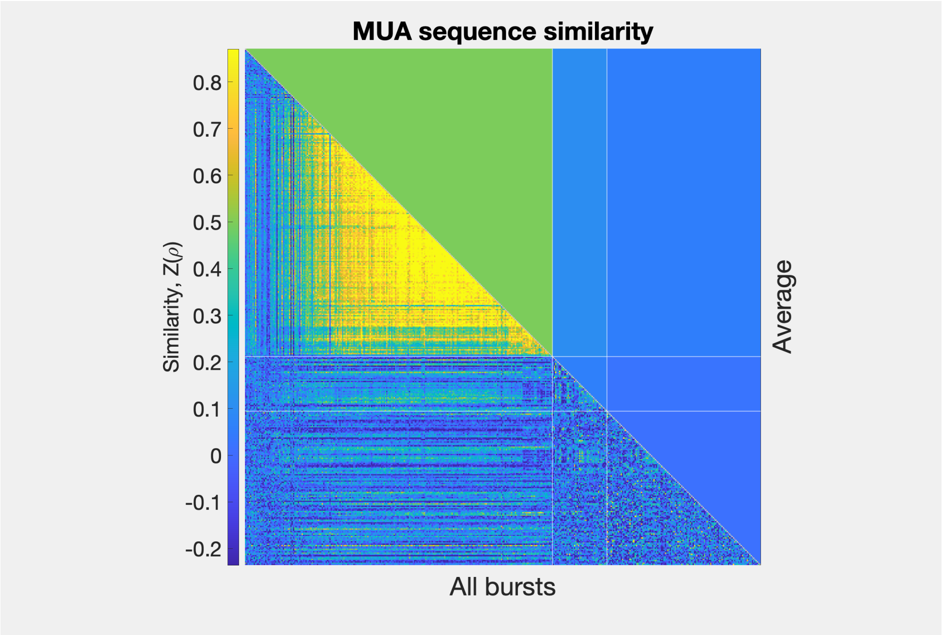

Supplementary Figure 32 Patient 1, array 1, seizure 3, burst similarity. For this patient, array, and seizure, every sequence was compared to every other sequence. Each cell indicates the similarity of the row sequence to the column sequence (Spearman's ρ). Sequences are divided by *white* lines into seizure sequences (*left, top*), IED sequences (*middle*), and baseline sequences (*right, bottom*). Actual values are provided in the lower triangle. In the upper triangle, average values are provided for each group, with values represented by the same color axis.

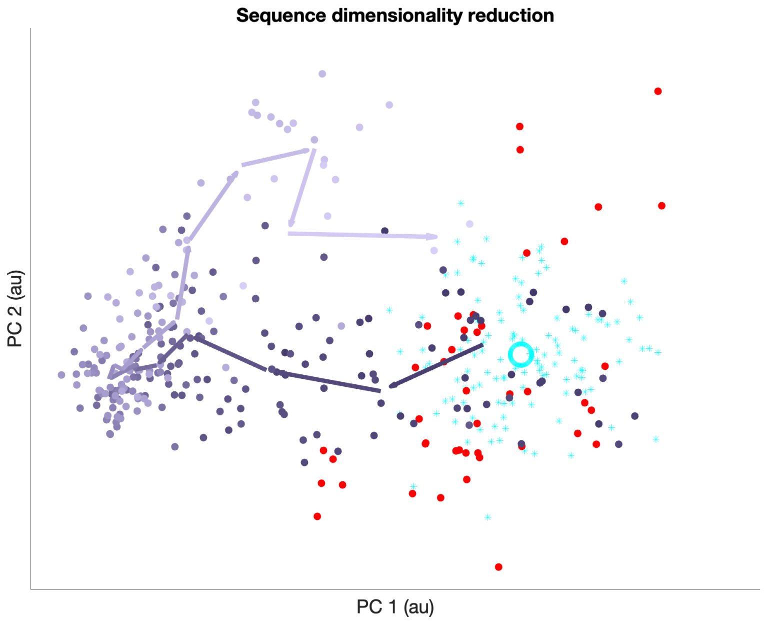

Supplementary Figure 33 Patient 1, array 1, seizure 3, dimensionality reduction using principal component analysis (PCA) was performed. We used PCA to decompose high-dimensional data into two dimensions in an unsupervised fashion (see *Methods*). Data provided include baseline bursts (*cyan stars*), IED bursts (*red dots*), and seizure bursts (*colored dots*; *dark purple*, early seizure, *light purple*, late seizure). The baseline centroid is indicated by the *cyan circle*.

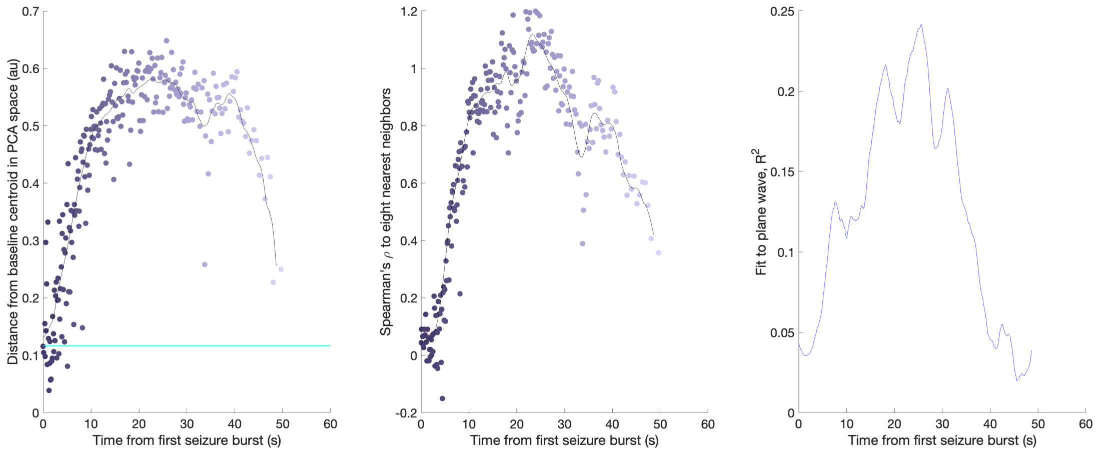

Supplementary Figure 34 Patient 1, array 1, seizure 3, measures of wave entrainment over time. *Left*: We used dimensionality reduction to collapse high-dimensional sequence information into two dimensions (see Figure 2a). We then took the location of the baseline centroid, and captured the distance, over time, between seizure bursts and the baseline centroid, in the low-dimensional manifold (see Figure 2c). *Dark purple* dots: early seizure; *light purple* dots: late seizure. A three-second moving average is superimposed (*black line*). Mean distance of baseline bursts to the baseline centroid is indicated by the *cyan line*. *Middle*: we captured Spearman's ρ between each seizure burst and its eight nearest temporal neighbors (see Figure 3a, b). This can be thought of as a measure of consistency of seizure bursts to each other in time. A three-second moving average of the ρ values is shown (*black line*). *Right*: for each burst, the R^2 value was determined from spatial linear regression (see *Methods*, section **Directionality of spike bursts and LFP discharges**). R^2 is a measure of the extent to which a burst is directional. A three-second moving average of the R^2 values is shown (*blue line*).

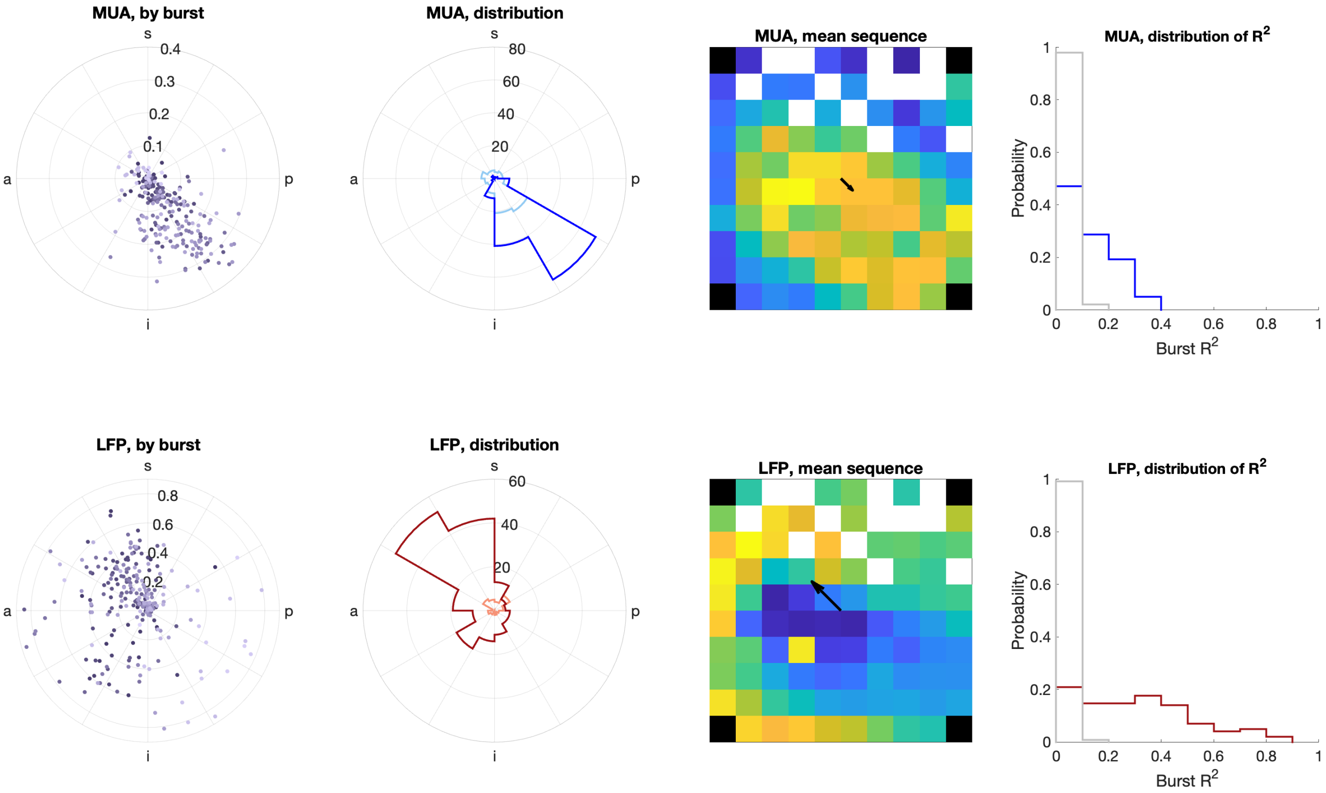

Supplementary Figure 35 Patient 1, array 1, seizure 3, seizure directionality. Top row: LFP. Bottom row: MUA. *Blue* histograms are used to designated MUA, while *maroon* histograms are used to designate LFP. First column from left: each dot represents a burst. θ reflects direction, and ρ is R^2, a measure of goodness of fit of the data to a plane. In other words, it is a measure of directionality of the burst. Second column from left: distribution direction of all recorded bursts. *Dark colors* indicate bursts with spatial linear regression giving $p \leq 0.05$, while *light* colors indicate bursts with $p > 0.05$. Third column from left: the average (*backbone*) sequence is shown over all bursts. This is obtained by taking the mean normalized rank of all sequences. The length of the *black arrow* indicates the mean R^2 over all bursts of this type, while the angle of the arrow indicates the mode of the histogram in the *second from left* column, for bursts with $p \leq 0.05$. Fourth column from left: R^2 for all bursts is shown. *Colored* histogram indicates the true sequences. *Gray* histogram, on the other hand, indicates R^2 for a set of null sequences, created by shuffling the ranks of the true sequences.

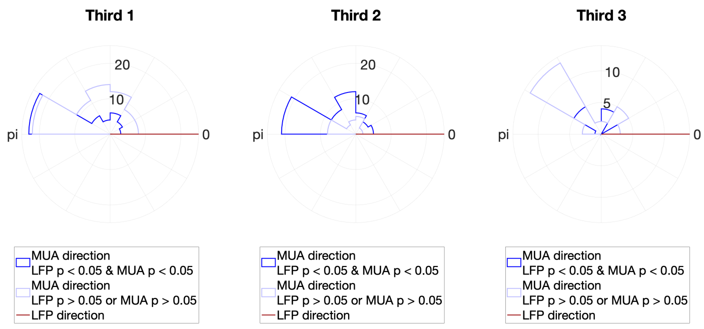

Supplementary Figure 36 Patient 1, array 1, seizure 3, relationship between MUA and LFP direction. Seizure bursts were divided into thirds (*left*: first third; *middle*: middle third, *right*: last third). For each third, we provided the distribution of burst-wise LFP-to-MUA directions. For each burst, LFP and MUA direction are compared, and the absolute value of the angular difference is put into the histogram. Bursts are divided into those with LFP and MUA p-value both less than 0.05 (*dark blue* histogram), and those with either MUA or LFP with p-value greater than 0.05. (*light blue* histogram).

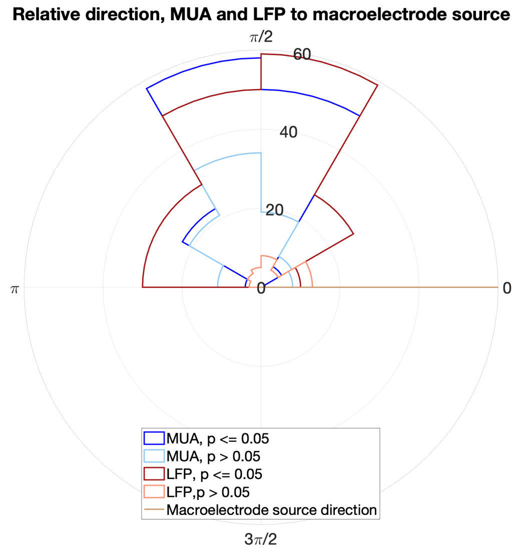

Supplementary Figure 37 Patient 1, array 1, seizure 3, relationship between discharge source, as determined by macroelectrode recordings, and direction of burst MUA and LFP. We used the time differences of the signal recorded in macroelectrodes to determine the location of the discharge source (Diamond, et al 2021, Diamond, et al 2023, see Supplementary Figure 12). The shortest path from the source to the array was then obtained, and we measured the angle of approach of the shortest path to the array (*tan* line). We then determined the relative direction of MUA and LFP signal, for each burst, to the discharge source as measured by macroelectrodes. Histograms are then provided, for the absolute value of the relative direction between source and MUA sequences (*blue*) and source and LFP sequences (*red*), for significant sequences (*dark* colors) and non-significant sequences (*light* colors).

Seizure activity, seizure 4

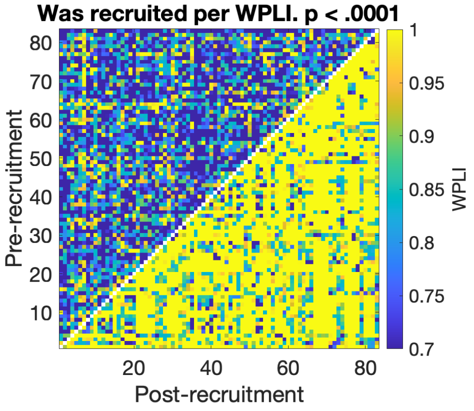

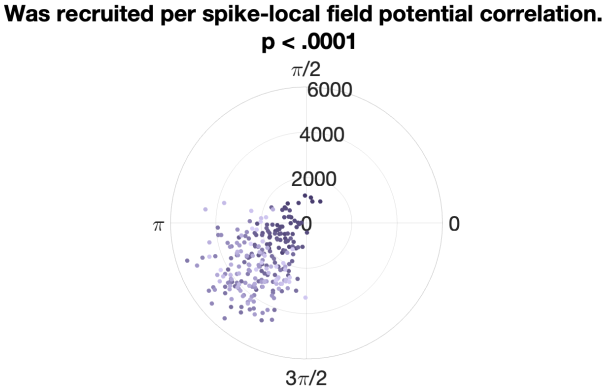

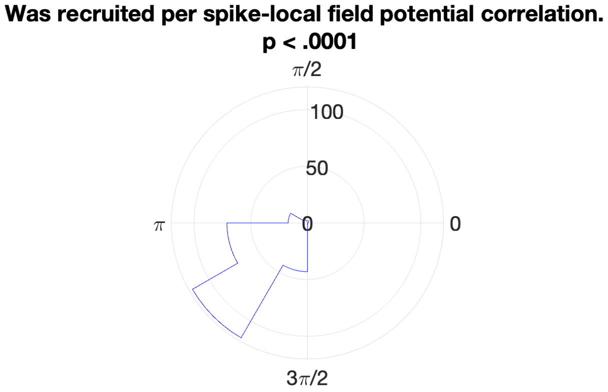

Supplementary Figure 38 Measures of recruitment for patient 1, array 1, seizure 4. Top: weighted phase lag index (WPLI), 10 seconds prior to seizure onset (top left), and 10 seconds after recruitment (bottom right, see *Methods*). Bottom left: relationship between LFP phase (θ) and LFP phase power (ρ), over the course of the seizure. *Dark purple* dots: early seizure; *light purple* dots: late seizure. Bottom right: distribution of spike-local field correlation values.

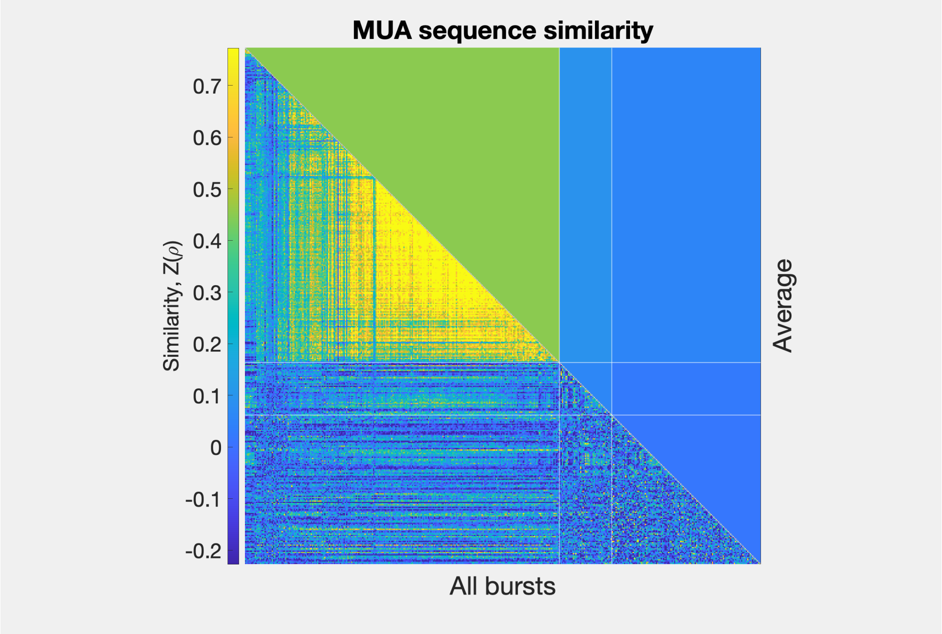

Supplementary Figure 39 Patient 1, array 1, seizure 4, burst similarity. For this patient, array, and seizure, every sequence was compared to every other sequence. Each cell indicates the similarity of the row sequence to the column sequence (Spearman's ρ). Sequences are divided by *white* lines into seizure sequences (*left, top*), IED sequences (*middle*), and baseline sequences (*right, bottom*). Actual values are provided in the lower triangle. In the upper triangle, average values are provided for each group, with values represented by the same color axis.

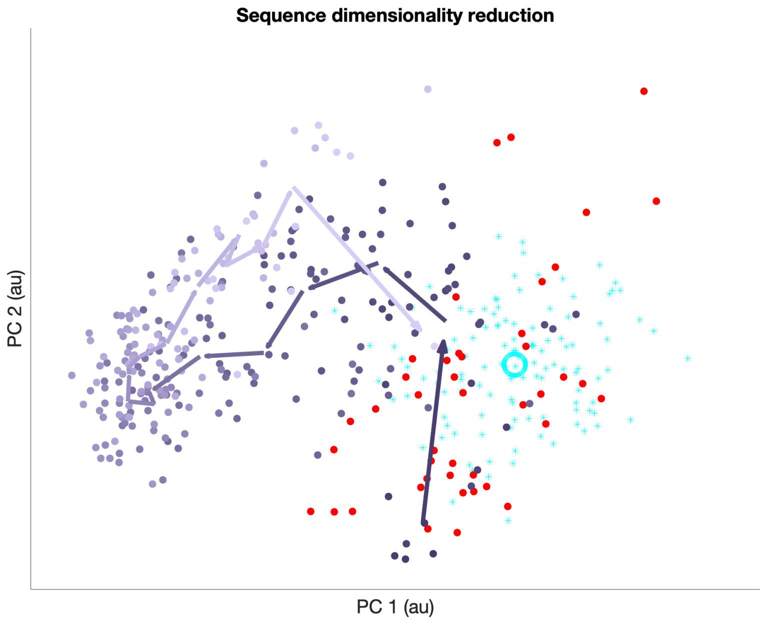

Supplementary Figure 40 Patient 1, array 1, seizure 4, dimensionality reduction using principal component analysis (PCA) was performed. We used PCA to decompose high-dimensional data into two dimensions in an unsupervised fashion (see *Methods*). Data provided include baseline bursts (*cyan stars*), IED bursts (*red dots*), and seizure bursts (*colored dots*; *dark purple*, early seizure, *light purple*, late seizure). The baseline centroid is indicated by the *cyan circle*.

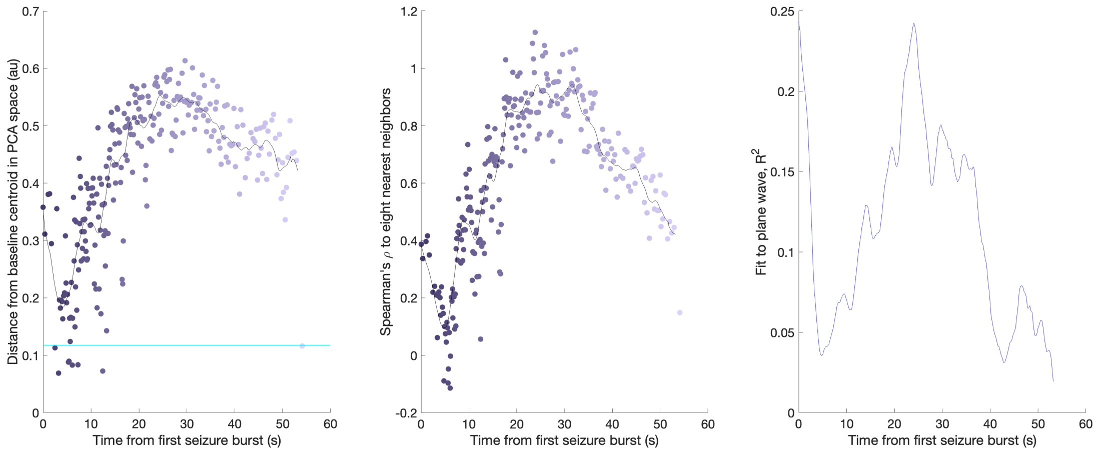

Supplementary Figure 41 Patient 1, array 1, seizure 4, measures of wave entrainment over time. *Left*: We used dimensionality reduction to collapse high-dimensional sequence information into two dimensions (see Figure 2a). We then took the location of the baseline centroid, and captured the distance, over time, between seizure bursts and the baseline centroid, in the low-dimensional manifold (see Figure 2c). *Dark purple* dots: early seizure; *light purple* dots: late seizure. A three-second moving average is superimposed (*black line*). Mean distance of baseline bursts to the baseline centroid is indicated by the *cyan line*. *Middle*: we captured Spearman's ρ between each seizure burst and its eight nearest temporal neighbors (see Figure 3a, b). This can be thought of as a measure of consistency of seizure bursts to each other in time. A three-second moving average of the ρ values is shown (*black line*). *Right*: for each burst, the R^2 value was determined from spatial linear regression (see *Methods*, section **Directionality of spike bursts and LFP discharges**). R^2 is a measure of the extent to which a burst is directional. A three-second moving average of the R^2 values is shown (*blue line*).

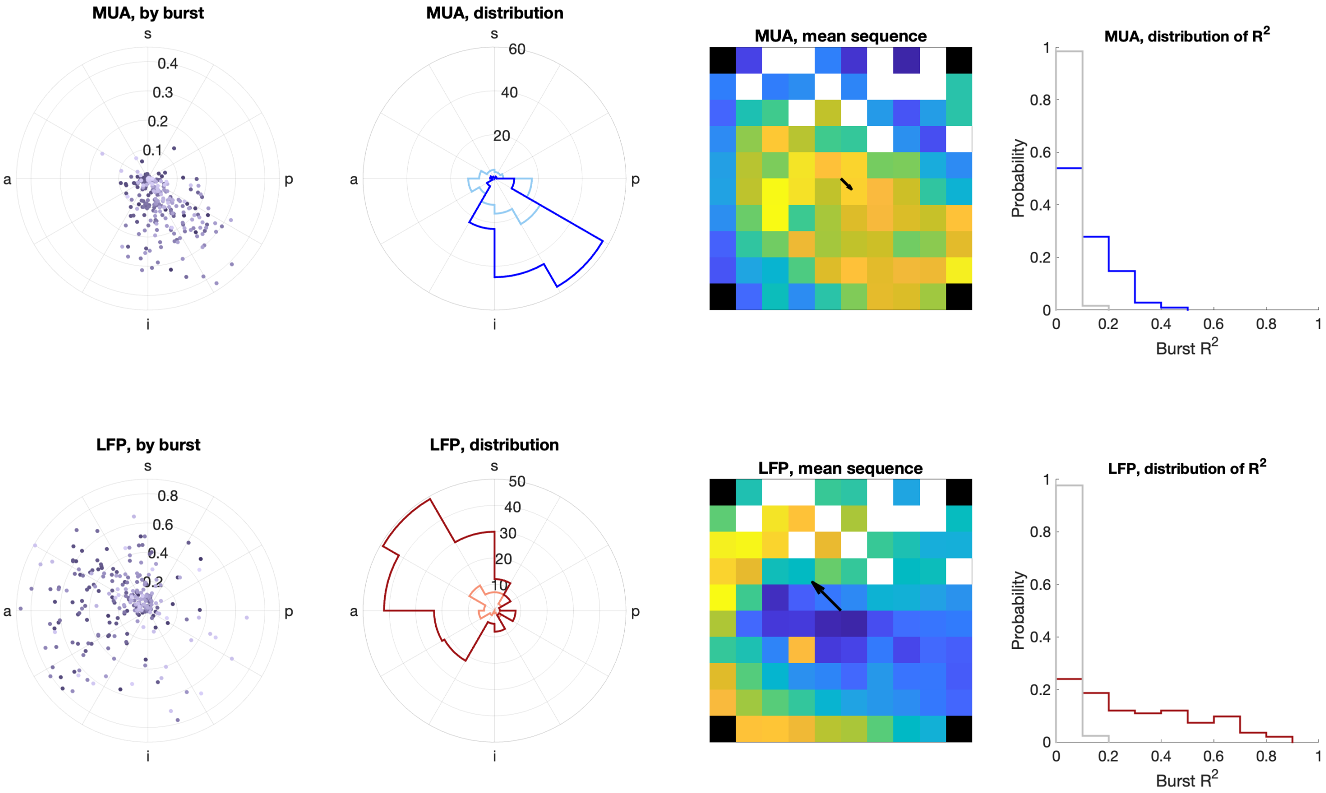

Supplementary Figure 42 Patient 1, array 1, seizure 4, seizure directionality. Top row: LFP. Bottom row: MUA. *Blue* histograms are used to designated MUA, while *maroon* histograms are used to designate LFP. First column from left: each dot represents a burst. θ reflects direction, and ρ is R^2, a measure of goodness of fit of the data to a plane. In other words, it is a measure of directionality of the burst. Second column from left: distribution direction of all recorded bursts. *Dark colors* indicate bursts with spatial linear regression giving $p \leq 0.05$, while *light* colors indicate bursts with $p > 0.05$. Third column from left: the average (*backbone*) sequence is shown over all bursts. This is obtained by taking the mean normalized rank of all sequences. The length of the *black arrow* indicates the mean R^2 over all bursts of this type, while the angle of the arrow indicates the mode of the histogram in the *second from left* column, for bursts with $p \leq 0.05$. Fourth column from left: R^2 for all bursts is shown. *Colored* histogram indicates the true sequences. *Gray* histogram, on the other hand, indicates R^2 for a set of null sequences, created by shuffling the ranks of the true sequences.

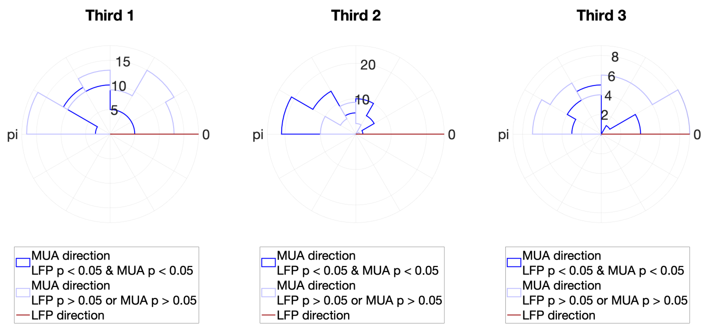

Supplementary Figure 43 Patient 1, array 1, seizure 4, relationship between MUA and LFP direction. Seizure bursts were divided into thirds (*left*: first third; *middle*: middle third, *right*: last third). For each third, we provided the distribution of burst-wise LFP-to-MUA directions. For each burst, LFP and MUA direction are compared, and the absolute value of the angular difference is put into the histogram. Bursts are divided into those with LFP and MUA p-value both less than 0.05 (*dark blue* histogram), and those with either MUA or LFP with p-value greater than 0.05. (*light blue* histogram).

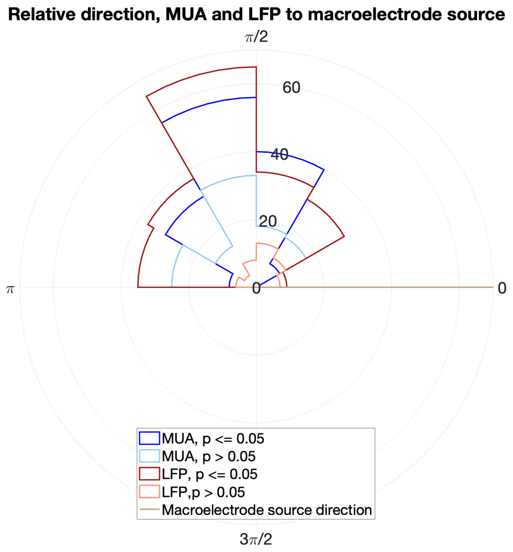

Supplementary Figure 44 Patient 1, array 1, seizure 4, relationship between discharge source, as determined by macroelectrode recordings, and direction of burst MUA and LFP. We used the time differences of the signal recorded in macroelectrodes to determine the location of the discharge source (Diamond, et al 2021, Diamond, et al 2023, see Supplementary Figure 12). The shortest path from the source to the array was then obtained, and we measured the angle of approach of the shortest path to the array (*tan* line). We then determined the relative direction of MUA and LFP signal, for each burst, to the discharge source as measured by macroelectrodes. Histograms are then provided, for the absolute value of the relative direction between source and MUA sequences (*blue*) and source and LFP sequences (*red*), for significant sequences (*dark* colors) and non-significant sequences (*light* colors).

Seizure activity, seizure 5

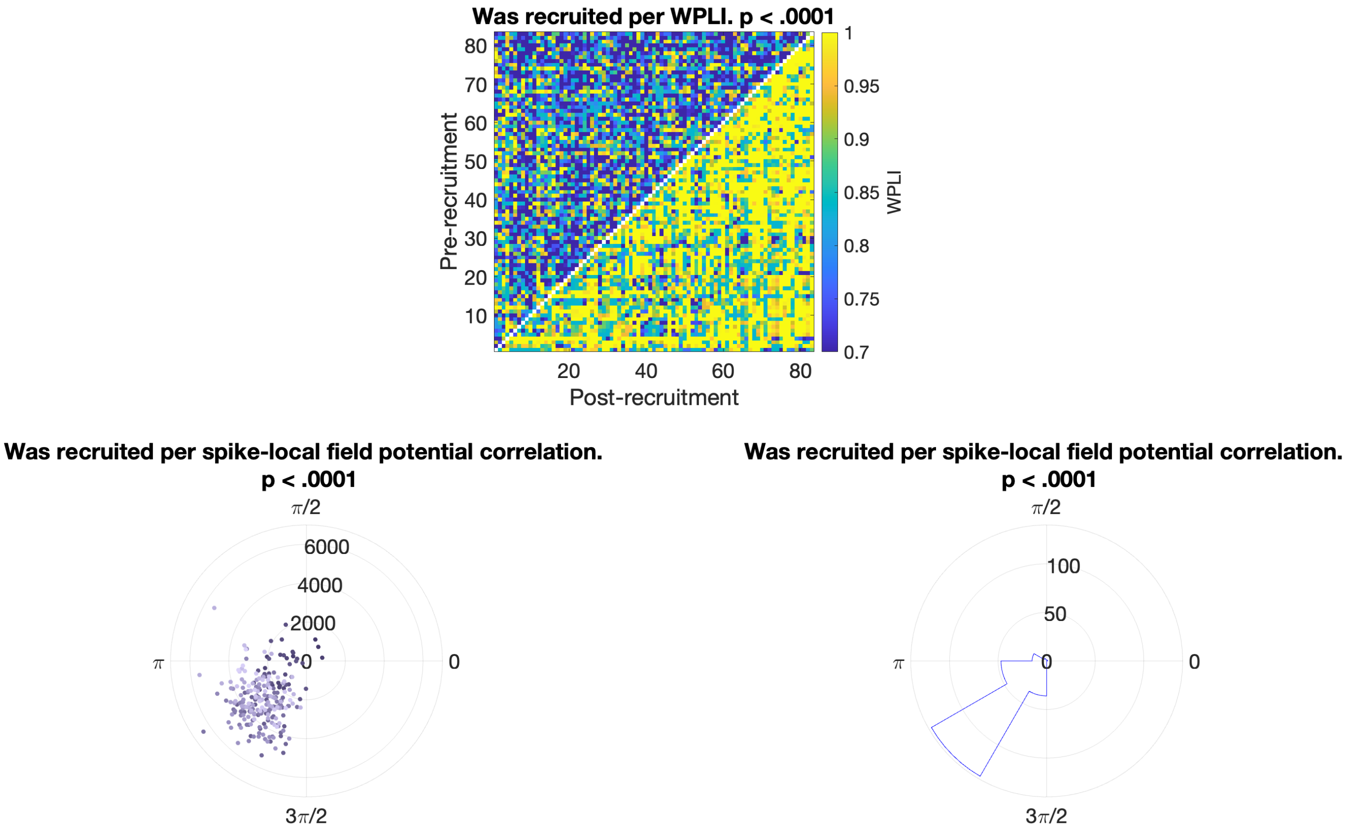

Supplementary Figure 45 Measures of recruitment for patient 1, array 1, seizure 5. Top: weighted phase lag index (WPLI), 10 seconds prior to seizure onset (top left), and 10 seconds after recruitment (bottom right, see *Methods*). Bottom left: relationship between LFP phase (θ) and LFP phase power (ρ), over the course of the seizure. *Dark purple* dots: early seizure; *light purple* dots: late seizure. Bottom right: distribution of spike-local field correlation values.

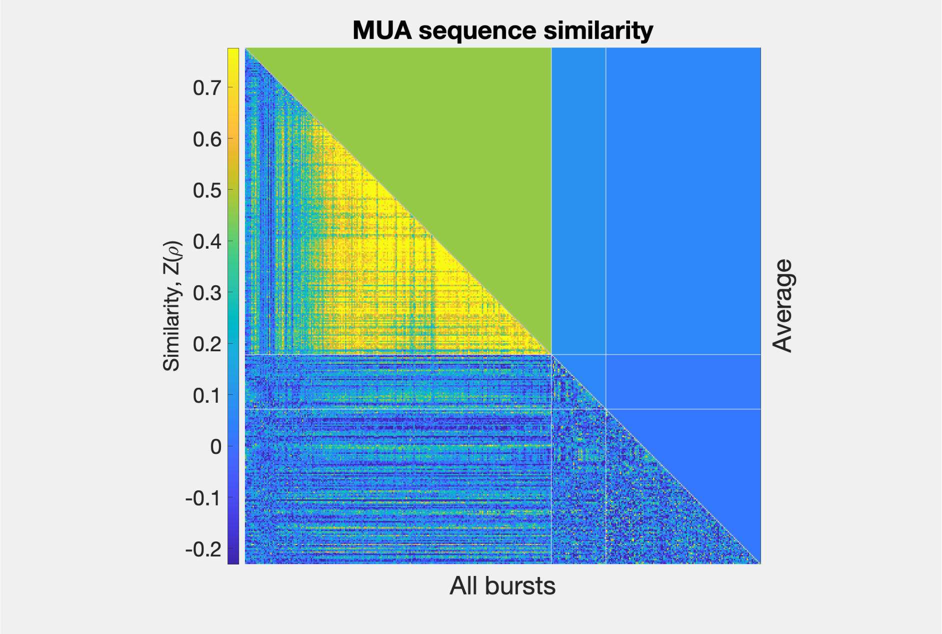

Supplementary Figure 46 Patient 1, array 1, seizure 5, burst similarity. For this patient, array, and seizure, every sequence was compared to every other sequence. Each cell indicates the similarity of the row sequence to the column sequence (Spearman's ρ). Sequences are divided by *white* lines into seizure sequences (*left, top*), IED sequences (*middle*), and baseline sequences (*right, bottom*). Actual values are provided in the lower triangle. In the upper triangle, average values are provided for each group, with values represented by the same color axis.

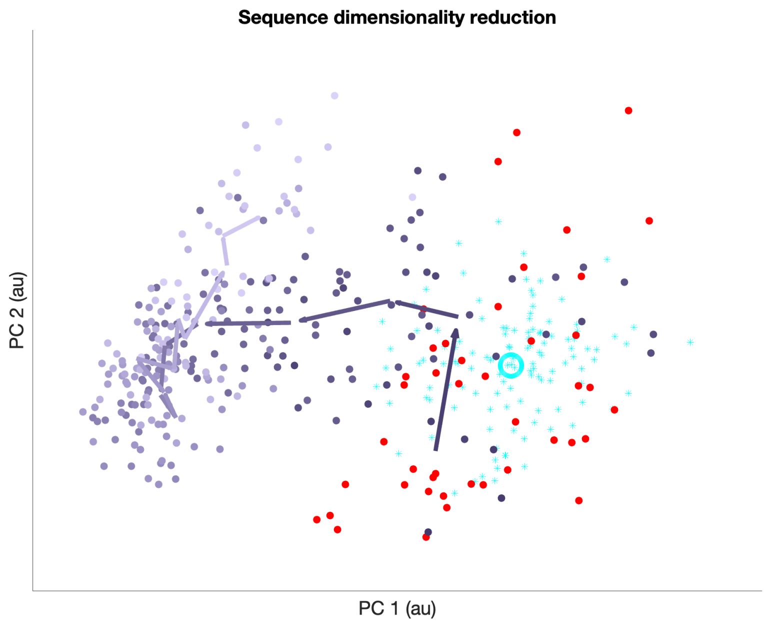

Supplementary Figure 47 Patient 1, array 1, seizure 5, dimensionality reduction using principal component analysis (PCA) was performed. We used PCA to decompose high-dimensional data into two dimensions in an unsupervised fashion (see *Methods*). Data provided include baseline bursts (*cyan stars*), IED bursts (*red dots*), and seizure bursts (*colored dots*; *dark purple*, early seizure, *light purple*, late seizure). The baseline centroid is indicated by the *cyan circle*.

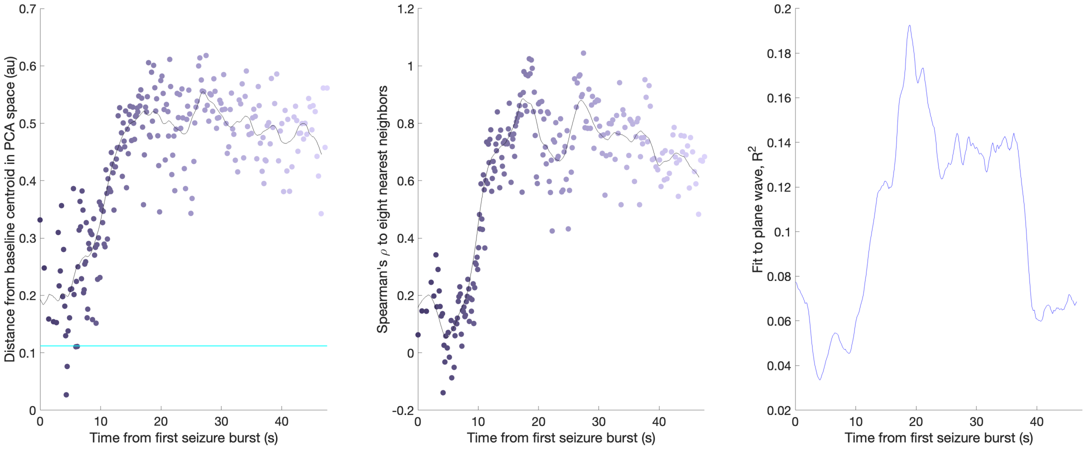

Supplementary Figure 48 Patient 1, array 1, seizure 5, measures of wave entrainment over time. *Left*: We used dimensionality reduction to collapse high-dimensional sequence information into two dimensions (see Figure 2a). We then took the location of the baseline centroid, and captured the distance, over time, between seizure bursts and the baseline centroid, in the low-dimensional manifold (see Figure 2c). *Dark purple* dots: early seizure; *light purple* dots: late seizure. A three-second moving average is superimposed (*black line*). Mean distance of baseline bursts to the baseline centroid is indicated by the *cyan line*. *Middle*: we captured Spearman's ρ between each seizure burst and its eight nearest temporal neighbors (see Figure 3a, b). This can be thought of as a measure of consistency of seizure bursts to each other in time. A three-second moving average of the ρ values is shown (*black line*). *Right*: for each burst, the R^2 value was determined from spatial linear regression (see *Methods*, section **Directionality of spike bursts and LFP discharges**). R^2 is a measure of the extent to which a burst is directional. A three-second moving average of the R^2 values is shown (*blue line*).

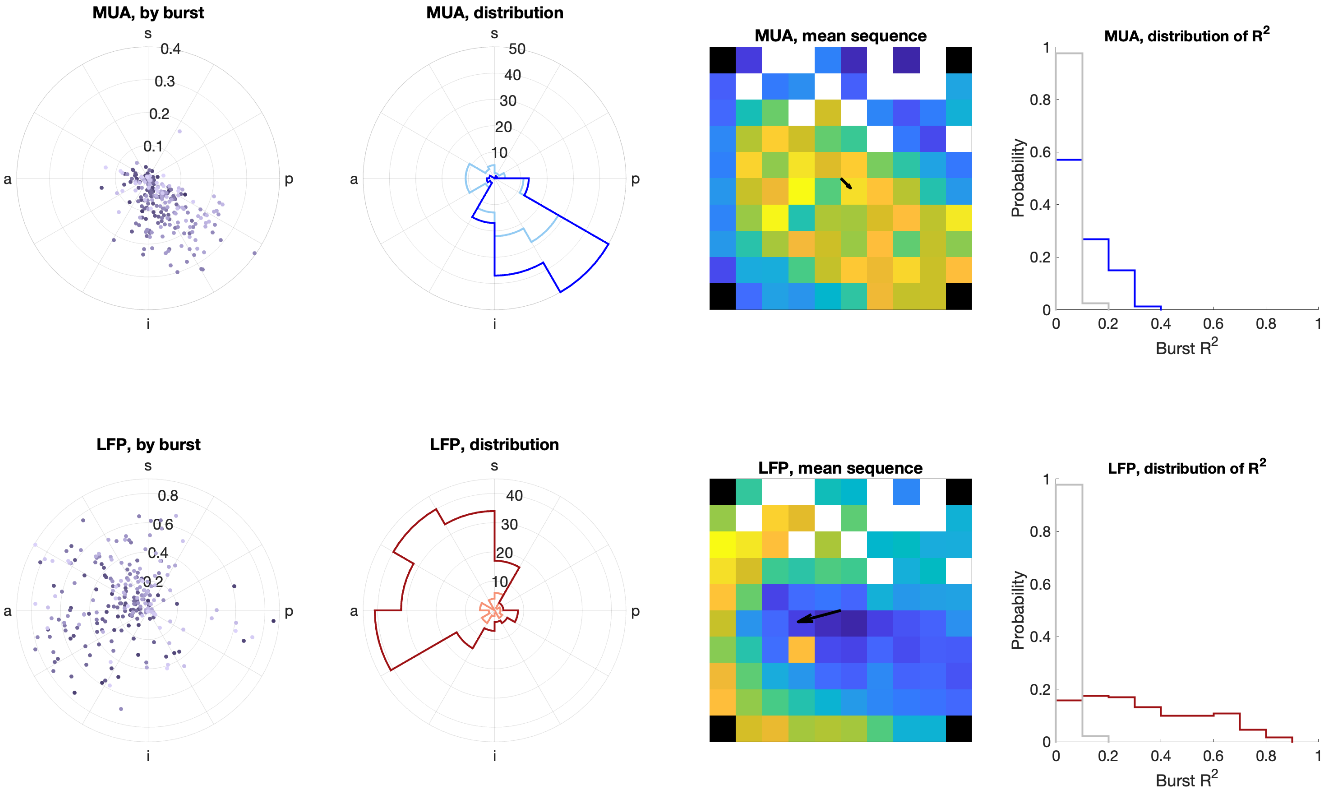

Supplementary Figure 49 Patient 1, array 1, seizure 5, seizure directionality. Top row: LFP. Bottom row: MUA. *Blue* histograms are used to designated MUA, while *maroon* histograms are used to designate LFP. First column from left: each dot represents a burst. θ reflects direction, and ρ is R^2, a measure of goodness of fit of the data to a plane. In other words, it is a measure of directionality of the burst. Second column from left: distribution direction of all recorded bursts. *Dark colors* indicate bursts with spatial linear regression giving $p \leq 0.05$, while *light* colors indicate bursts with $p > 0.05$. Third column from left: the average (*backbone*) sequence is shown over all bursts. This is obtained by taking the mean normalized rank of all sequences. The length of the *black arrow* indicates the mean R^2 over all bursts of this type, while the angle of the arrow indicates the mode of the histogram in the *second from left* column, for bursts with $p \leq 0.05$. Fourth column from left: R^2 for all bursts is shown. *Colored* histogram indicates the true sequences. *Gray* histogram, on the other hand, indicates R^2 for a set of null sequences, created by shuffling the ranks of the true sequences.

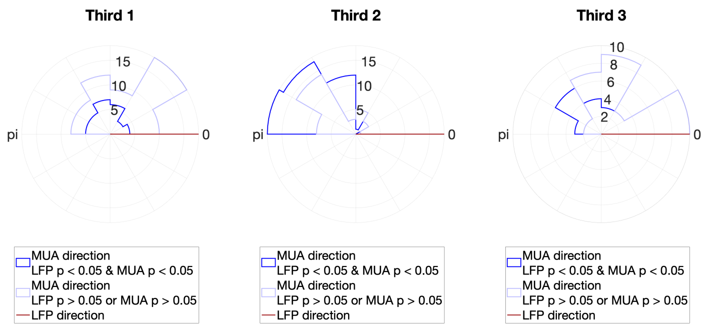

Supplementary Figure 50 Patient 1, array 1, seizure 5, relationship between MUA and LFP direction. Seizure bursts were divided into thirds (*left*: first third; *middle*: middle third, *right*: last third). For each third, we provided the distribution of burst-wise LFP-to-MUA directions. For each burst, LFP and MUA direction are compared, and the absolute value of the angular difference is put into the histogram. Bursts are divided into those with LFP and MUA p-value both less than 0.05 (*dark blue* histogram), and those with either MUA or LFP with p-value greater than 0.05. (*light blue* histogram).

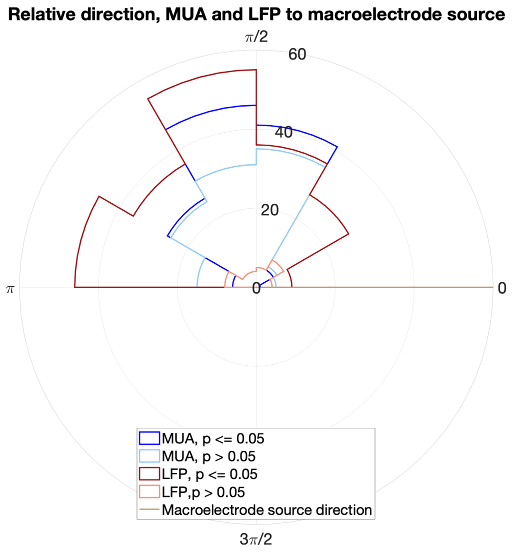

Supplementary Figure 51 Patient 1, array 1, seizure 5, relationship between discharge source, as determined by macroelectrode recordings, and direction of burst MUA and LFP. We used the time differences of the signal recorded in macroelectrodes to determine the location of the discharge source (Diamond, et al 2021, Diamond, et al 2023, see Supplementary Figure 12). The shortest path from the source to the array was then obtained, and we measured the angle of approach of the shortest path to the array (*tan* line). We then determined the relative direction of MUA and LFP signal, for each burst, to the discharge source as measured by macroelectrodes. Histograms are then provided, for the absolute value of the relative direction between source and MUA sequences (*blue*) and source and LFP sequences (*red*), for significant sequences (*dark* colors) and non-significant sequences (*light* colors).

Seizure activity, seizure 6

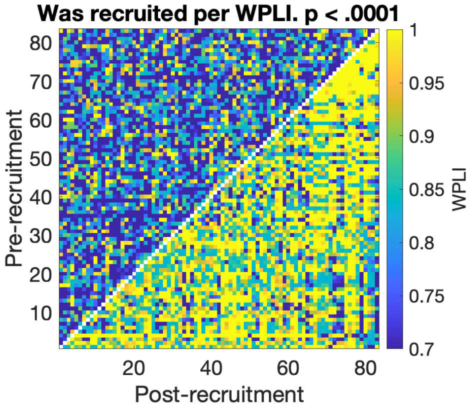

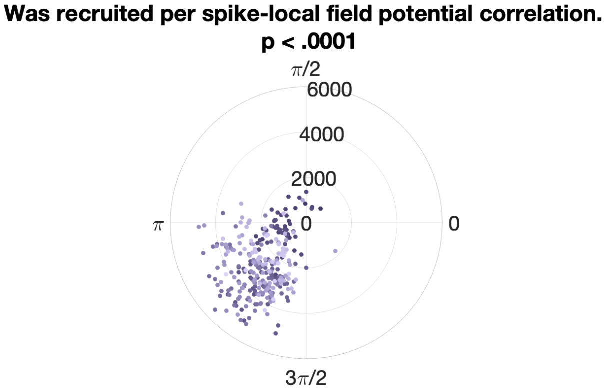

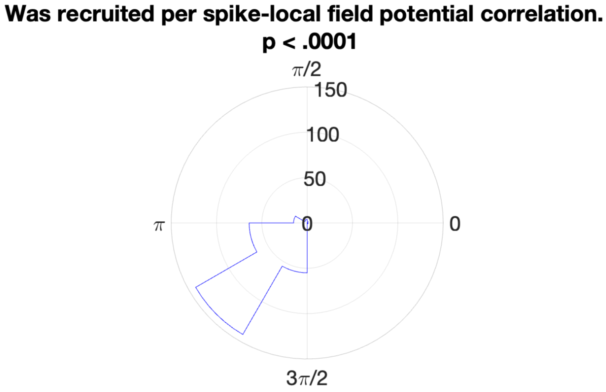

Supplementary Figure 52 Measures of recruitment for patient 1, array 1, seizure 6. Top: weighted phase lag index (WPLI), 10 seconds prior to seizure onset (top left), and 10 seconds after recruitment (bottom right, see *Methods*). Bottom left: relationship between LFP phase (θ) and LFP phase power (ρ), over the course of the seizure. *Dark purple* dots: early seizure; *light purple* dots: late seizure. Bottom right: distribution of spike-local field correlation values.

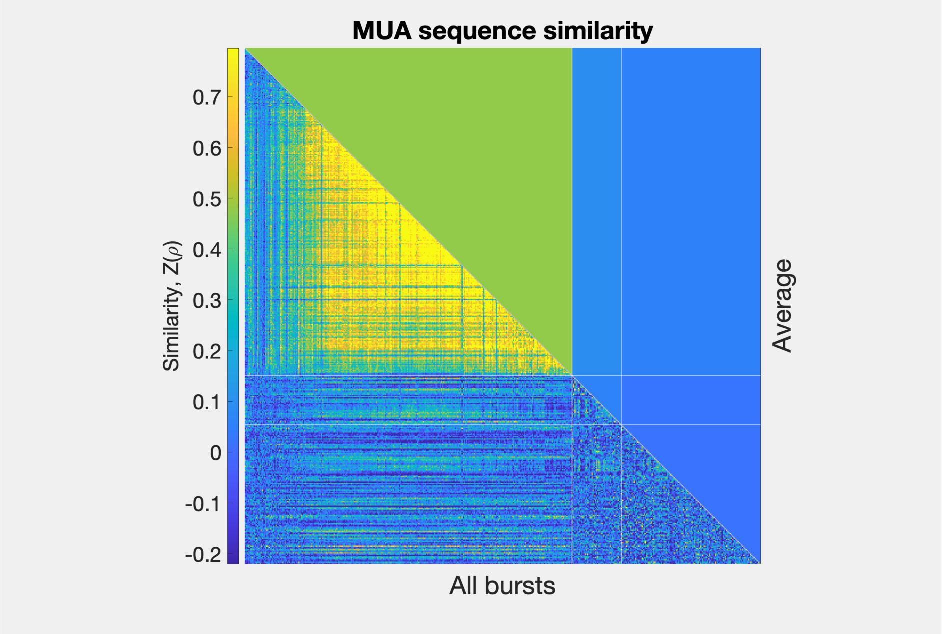

Supplementary Figure 53 Patient 1, array 1, seizure 6, burst similarity. For this patient, array, and seizure, every sequence was compared to every other sequence. Each cell indicates the similarity of the row sequence to the column sequence (Spearman's ρ). Sequences are divided by *white* lines into seizure sequences (*left, top*), IED sequences (*middle*), and baseline sequences (*right, bottom*). Actual values are provided in the lower triangle. In the upper triangle, average values are provided for each group, with values represented by the same color axis.

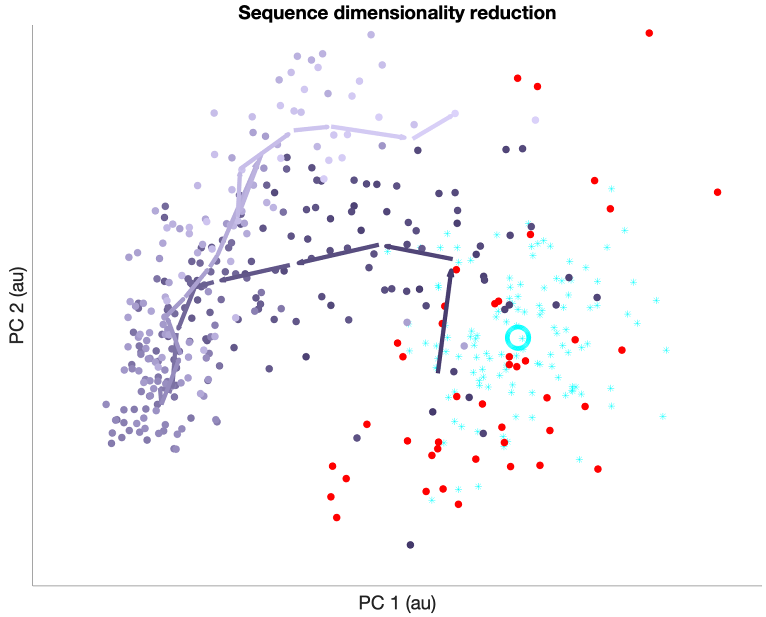

Supplementary Figure 54 Patient 1, array 1, seizure 6, dimensionality reduction using principal component analysis (PCA) was performed. We used PCA to decompose high-dimensional data into two dimensions in an unsupervised fashion (see *Methods*). Data provided include baseline bursts (*cyan stars*), IED bursts (*red dots*), and seizure bursts (*colored dots*; *dark purple*, early seizure, *light purple*, late seizure). The baseline centroid is indicated by the *cyan circle*.

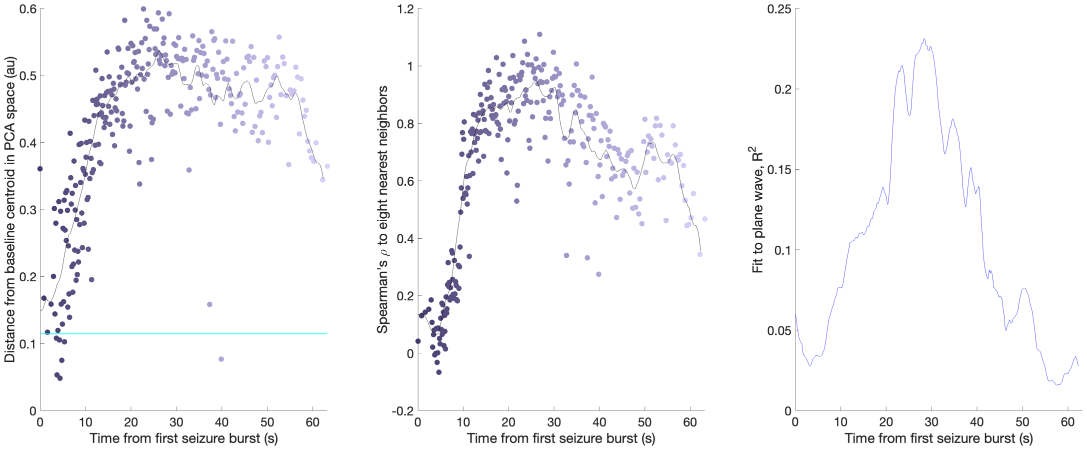

Supplementary Figure 55 Patient 1, array 1, seizure 6, measures of wave entrainment over time. *Left*: We used dimensionality reduction to collapse high-dimensional sequence information into two dimensions (see Figure 2a). We then took the location of the baseline centroid, and captured the distance, over time, between seizure bursts and the baseline centroid, in the low-dimensional manifold (see Figure 2c). *Dark purple* dots: early seizure; *light purple* dots: late seizure. A three-second moving average is superimposed (*black line*). Mean distance of baseline bursts to the baseline centroid is indicated by the *cyan line*. *Middle*: we captured Spearman's ρ between each seizure burst and its eight nearest temporal neighbors (see Figure 3a, b). This can be thought of as a measure of consistency of seizure bursts to each other in time. A three-second moving average of the ρ values is shown (*black line*). *Right*: for each burst, the R^2 value was determined from spatial linear regression (see *Methods*, section **Directionality of spike bursts and LFP discharges**). R^2 is a measure of the extent to which a burst is directional. A three-second moving average of the R^2 values is shown (*blue line*).

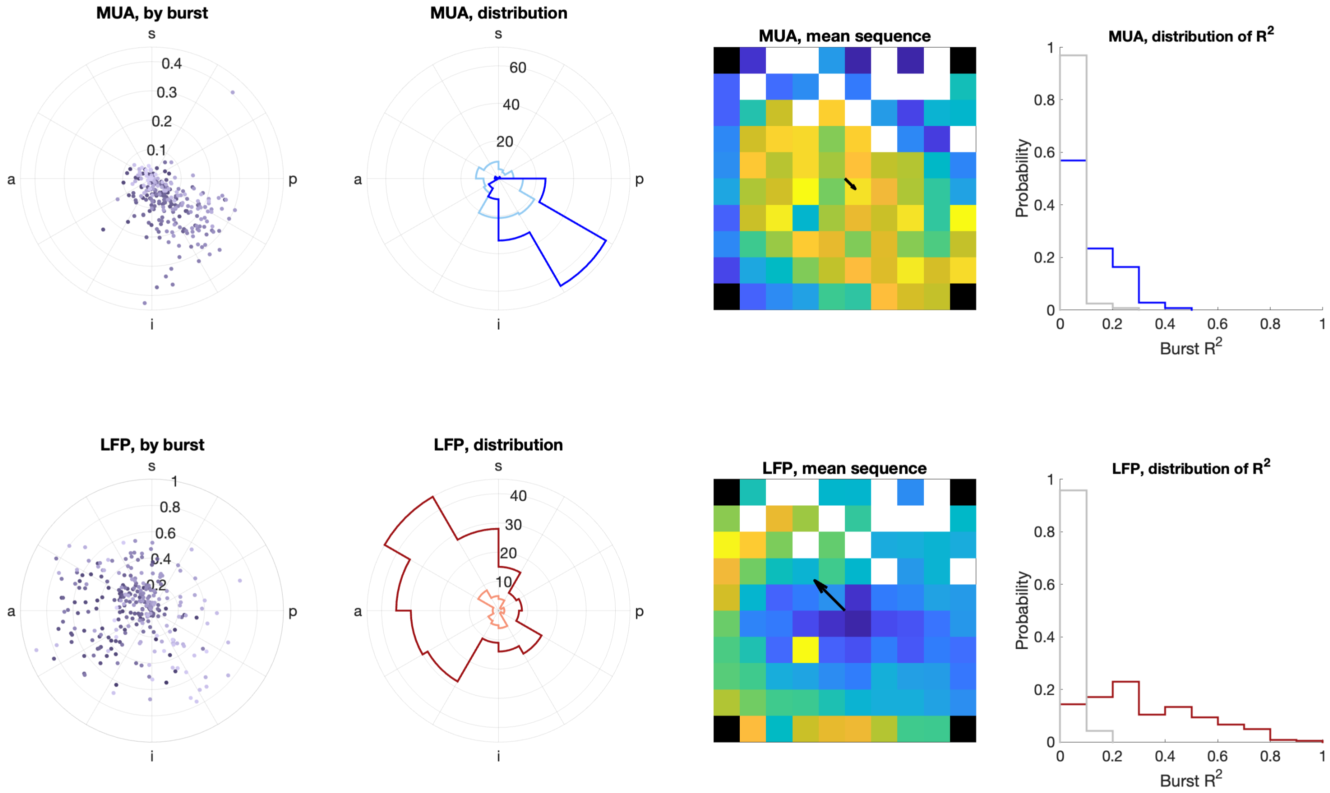

Supplementary Figure 56 Patient 1, array 1, seizure 6, seizure directionality. Top row: LFP. Bottom row: MUA. *Blue* histograms are used to designated MUA, while *maroon* histograms are used to designate LFP. First column from left: each dot represents a burst. θ reflects direction, and ρ is R^2, a measure of goodness of fit of the data to a plane. In other words, it is a measure of directionality of the burst. Second column from left: distribution direction of all recorded bursts. *Dark colors* indicate bursts with spatial linear regression giving $p \leq 0.05$, while *light* colors indicate bursts with $p > 0.05$. Third column from left: the average (*backbone*) sequence is shown over all bursts. This is obtained by taking the mean normalized rank of all sequences. The length of the *black arrow* indicates the mean R^2 over all bursts of this type, while the angle of the arrow indicates the mode of the histogram in the *second from left* column, for bursts with $p \leq 0.05$. Fourth column from left: R^2 for all bursts is shown. *Colored* histogram indicates the true sequences. *Gray* histogram, on the other hand, indicates R^2 for a set of null sequences, created by shuffling the ranks of the true sequences.

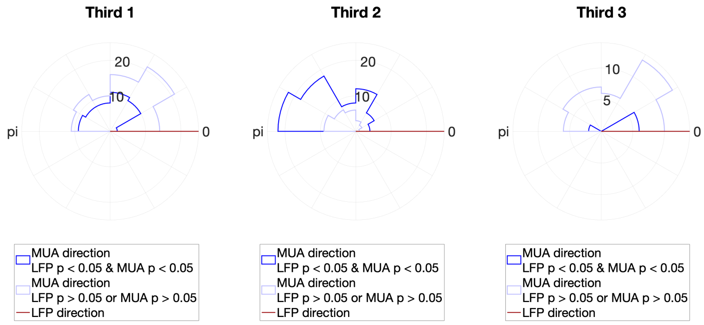

Supplementary Figure 57 Patient 1, array 1, seizure 6, relationship between MUA and LFP direction. Seizure bursts were divided into thirds (*left*: first third; *middle*: middle third, *right*: last third). For each third, we provided the distribution of burst-wise LFP-to-MUA directions. For each burst, LFP and MUA direction are compared, and the absolute value of the angular difference is put into the histogram. Bursts are divided into those with LFP and MUA p-value both less than 0.05 (*dark blue* histogram), and those with either MUA or LFP with p-value greater than 0.05. (*light blue* histogram).

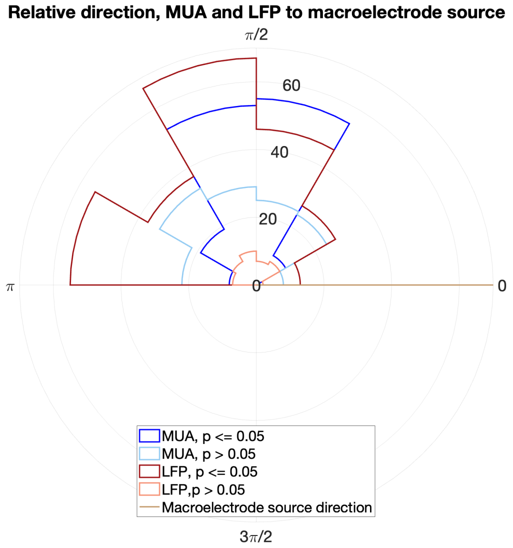

Supplementary Figure 58 Patient 1, array 1, seizure 6, relationship between discharge source, as determined by macroelectrode recordings, and direction of burst MUA and LFP. We used the time differences of the signal recorded in macroelectrodes to determine the location of the discharge source (Diamond, et al 2021, Diamond, et al 2023, see Supplementary Figure 12). The shortest path from the source to the array was then obtained, and we measured the angle of approach of the shortest path to the array (*tan* line). We then determined the relative direction of MUA and LFP signal, for each burst, to the discharge source as measured by macroelectrodes. Histograms are then provided, for the absolute value of the relative direction between source and MUA sequences (*blue*) and source and LFP sequences (*red*), for significant sequences (*dark* colors) and non-significant sequences (*light* colors).

Patient 2, array 1

Baseline activity

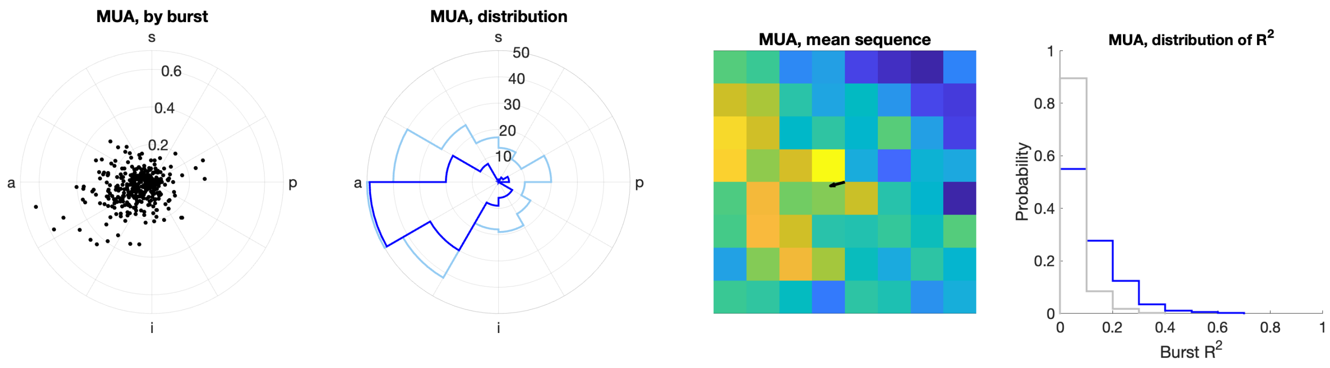

Supplementary Figure 59 Patient 2, array 1, baseline wavelike properties. First panel from left: each dot represents a burst. θ reflects direction, and ρ is R^2, a measure of goodness of fit of the data to a plane. In other words, it is a measure of the 'wavelike-ness' of the burst. Second panel from left: distribution direction of all recorded bursts. *Dark* colors indicate bursts with spatial linear regression giving $p \leq 0.05$, while *light* colors indicate bursts with $p > 0.05$. Third panel from left: the average (*backbone*) sequence is shown over all bursts. This is obtained by taking the mean normalized rank of all sequences. The length of the *black arrow* indicates the mean R^2 over all bursts of this type, while the angle of the arrow indicates the mode of the histogram in the *second from left* panel, for bursts with $p \leq 0.05$. Fourth panel from left: R^2 for all bursts is shown. *Blue* histogram indicates the true sequences. *Gray* histogram, on the other hand, indicates R^2 for a set of null sequences, created by shuffling the ranks of the true sequences.

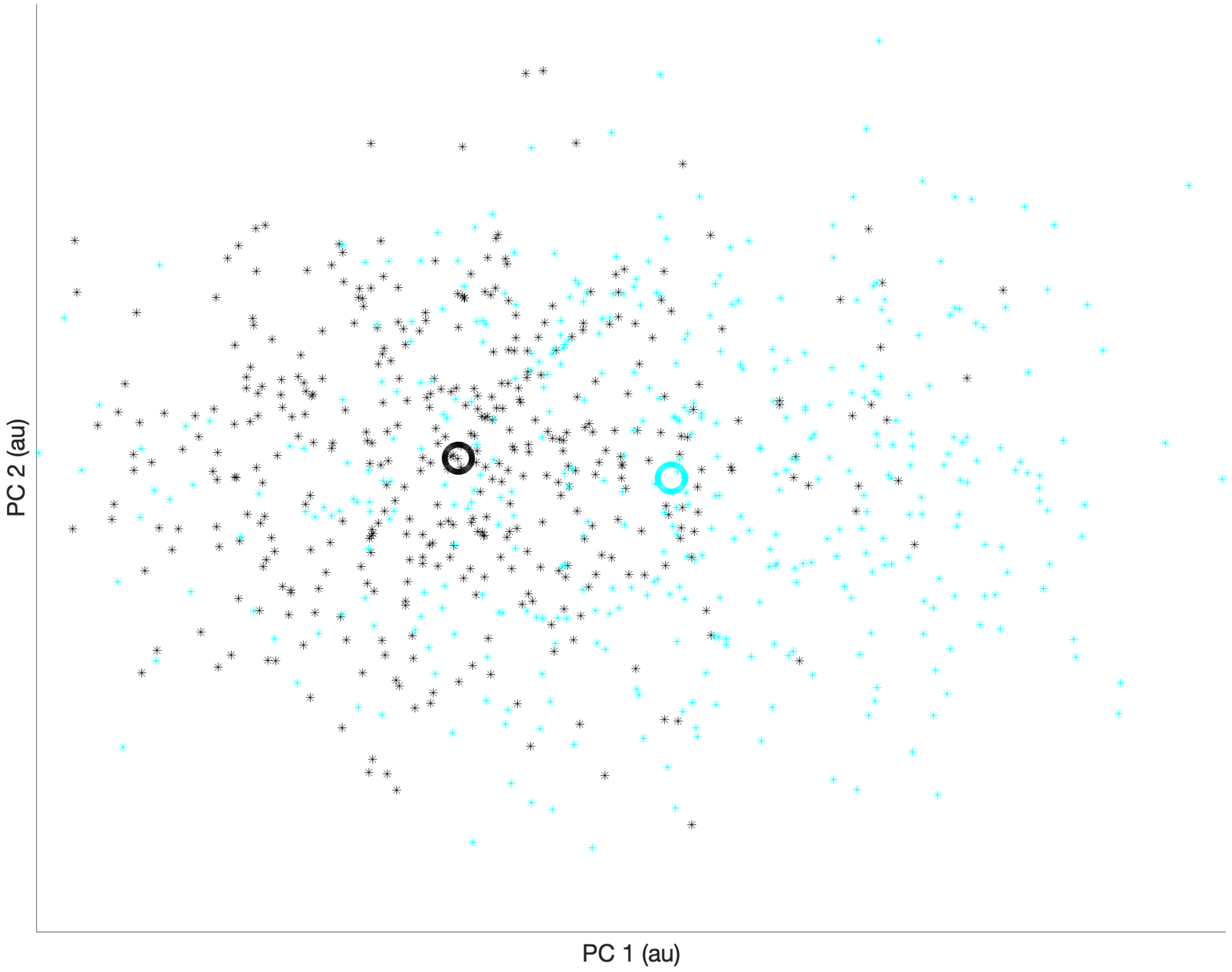

Supplementary Figure 60 Patient 2, array 1, baseline bursts versus random bursts. We were interested in determining that our baseline bursts were non-random. Therefore, for each baseline burst, we created a null counterpart, in which the MUA timings were shuffled only among spiking electrodes. We then applied both the true baselines and the random counterparts to the same dimensionality reduction procedure. The baseline centroid is indicated by the *blue circle*. The random centroid indicated by the *black* circle. For most patients and arrays, baseline bursts are displaced from the random bursts. Distance from baseline bursts to the random centroid tends to be greater than distance from random bursts to the random centroid (Supplementary Figure 4).

IED activity

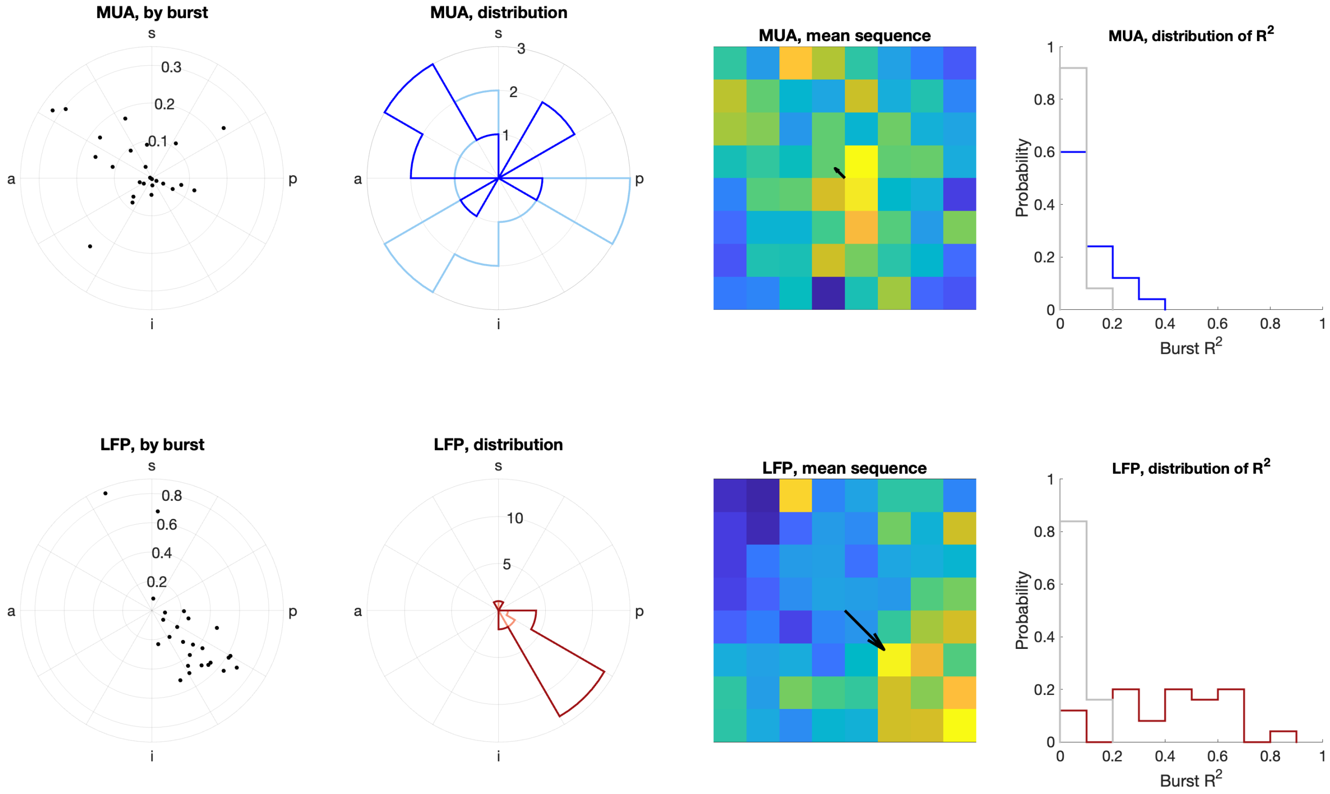

Supplementary Figure 61 Patient 2, array 1, IED directionality. Top row: LFP. Bottom row: MUA. *Dark blue* histograms are used to designated MUA, while *maroon* histograms are used to designate LFP. First column from left: each dot represents a burst. θ reflects direction, and ρ is R^2, a measure of goodness of fit of the data to a plane. In other words, it is a measure of the strength of directionality of the burst. Second column from left: distribution direction of all recorded bursts. *Dark colors* indicate bursts with spatial linear regression giving $p \leq 0.05$, while *light* colors indicate bursts with $p > 0.05$. Third column from left: the average (*backbone*) sequence is shown over all bursts for this seizure. This is obtained by taking the mean normalized rank of all sequences. The length of the *black arrow* indicates the mean R^2 over all bursts of this type, while the angle of the arrow indicates the mode of the histogram in the *second from left* column, for bursts with $p \leq 0.05$. Fourth column from left: R^2 for all bursts is shown. *Colored* histogram indicates the true sequences. *Gray* histogram, on the other hand, indicates R^2 for a set of null sequences, created by shuffling the ranks of spiking electrodes in the true sequences.

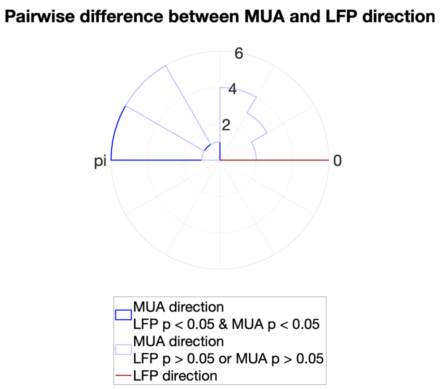

Supplementary Figure 62 Patient 2, array 1, IED relationship between MUA and LFP direction. Distribution of pairwise LFP-to-MUA direction. For each burst, LFP and MUA direction are compared, and the absolute value of the angular difference is put into the histogram. Bursts are divided into those with LFP and MUA p-value both less than 0.05, or those with either MUA or LFP with p-value greater than 0.05.

Seizure activity, seizure 1

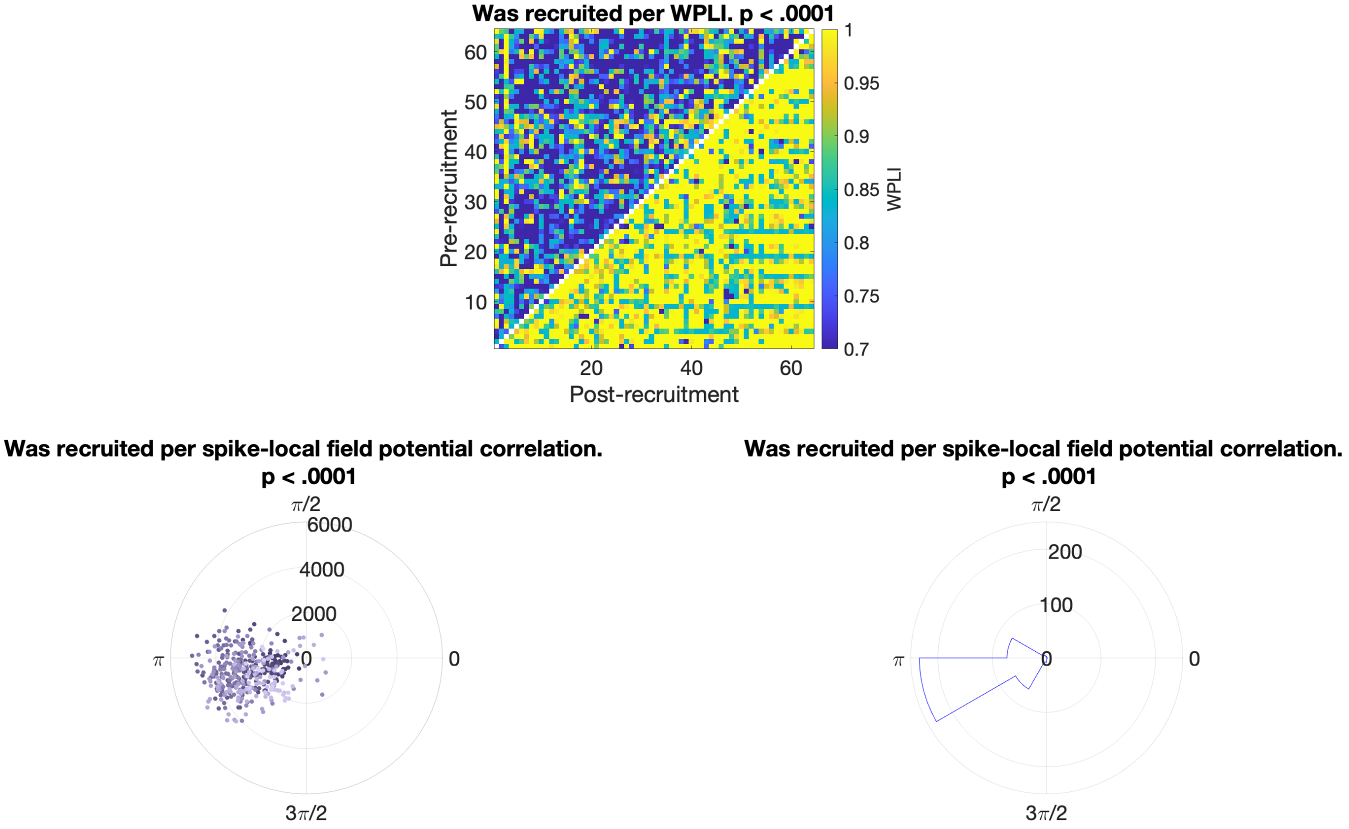

Supplementary Figure 63 Measures of recruitment for patient 2, array 1, seizure 1. Top: weighted phase lag index (WPLI), 10 seconds prior to seizure onset (top left), and 10 seconds after recruitment (bottom right, see *Methods*). Bottom left: relationship between LFP phase (θ) and LFP phase power (ρ), over the course of the seizure. *Dark purple* dots: early seizure; *light purple* dots: late seizure. Bottom right: distribution of spike-local field correlation values.

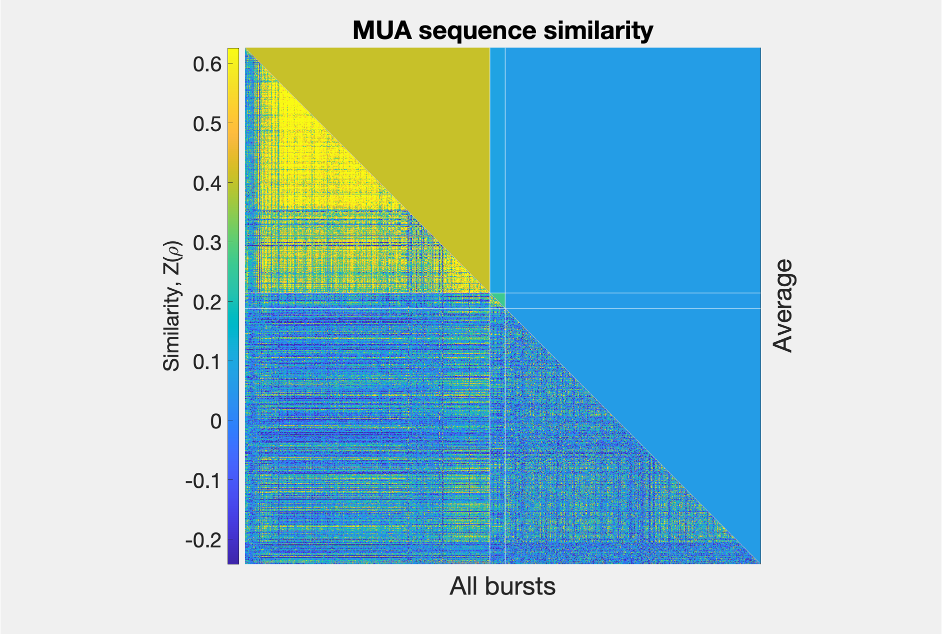

Supplementary Figure 64 Patient 2, array 1, seizure 1, burst similarity. For this patient, array, and seizure, every sequence was compared to every other sequence. Each cell indicates the similarity of the row sequence to the column sequence (Spearman's ρ). Sequences are divided by *white* lines into seizure sequences (*left, top*), IED sequences (*middle*), and baseline sequences (*right, bottom*). Actual values are provided in the lower triangle. In the upper triangle, average values are provided for each group, with values represented by the same color axis.

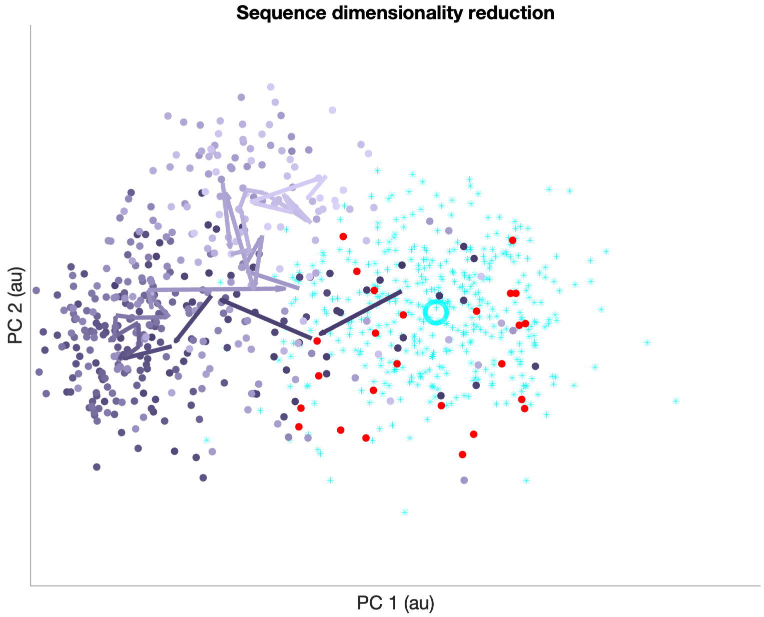

Supplementary Figure 65 Patient 2, array 1, seizure 1, dimensionality reduction using principal component analysis (PCA) was performed. We used PCA to decompose high-dimensional data into two dimensions in an unsupervised fashion (see *Methods*). Data provided include baseline bursts (*cyan stars*), IED bursts (*red dots*), and seizure bursts (*colored dots*; *dark purple*, early seizure, *light purple*, late seizure). The baseline centroid is indicated by the *cyan circle*.

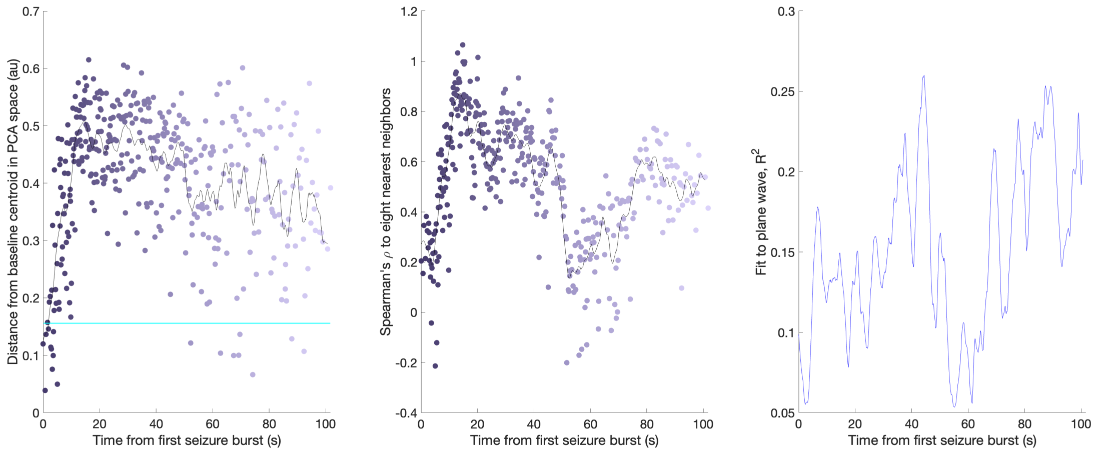

Supplementary Figure 66 Patient 2, array 1, seizure 1, measures of wave entrainment over time. *Left*: We used dimensionality reduction to collapse high-dimensional sequence information into two dimensions (see Figure 2a). We then took the location of the baseline centroid, and captured the distance, over time, between seizure bursts and the baseline centroid, in the low-dimensional manifold (see Figure 2c). *Dark purple* dots: early seizure; *light purple* dots: late seizure. A three-second moving average is superimposed (*black line*). Mean distance of baseline bursts to the baseline centroid is indicated by the *cyan line*. *Middle*: we captured Spearman's ρ between each seizure burst and its eight nearest temporal neighbors (see Figure 3a, b). This can be thought of as a measure of consistency of seizure bursts to each other in time. A three-second moving average of the ρ values is shown (*black line*). *Right*: for each burst, the R^2 value was determined from spatial linear regression (see *Methods*, section **Directionality of spike bursts and LFP discharges**). R^2 is a measure of the extent to which a burst is directional. A three-second moving average of the R^2 values is shown (*blue line*).

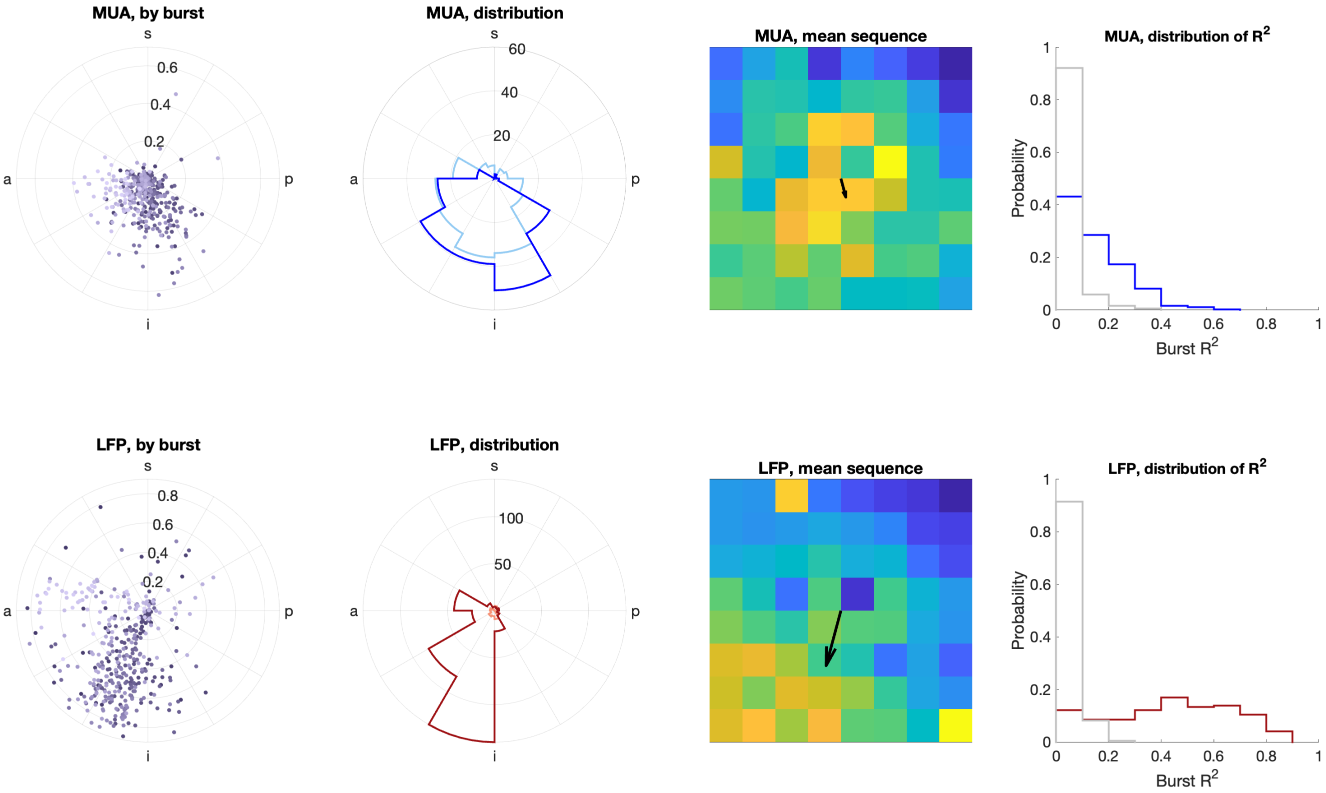

Supplementary Figure 67 Patient 2, array 1, seizure 1, seizure directionality. Top row: LFP. Bottom row: MUA. *Blue* histograms are used to designated MUA, while *maroon* histograms are used to designate LFP. First column from left: each dot represents a burst. θ reflects direction, and ρ is R^2, a measure of goodness of fit of the data to a plane. In other words, it is a measure of directionality of the burst. Second column from left: distribution direction of all recorded bursts. *Dark colors* indicate bursts with spatial linear regression giving $p \leq 0.05$, while *light* colors indicate bursts with $p > 0.05$. Third column from left: the average (*backbone*) sequence is shown over all bursts. This is obtained by taking the mean normalized rank of all sequences. The length of the *black arrow* indicates the mean R^2 over all bursts of this type, while the angle of the arrow indicates the mode of the histogram in the *second from left* column, for bursts with $p \leq 0.05$. Fourth column from left: R^2 for all bursts is shown. *Colored* histogram indicates the true sequences. *Gray* histogram, on the other hand, indicates R^2 for a set of null sequences, created by shuffling the ranks of the true sequences.

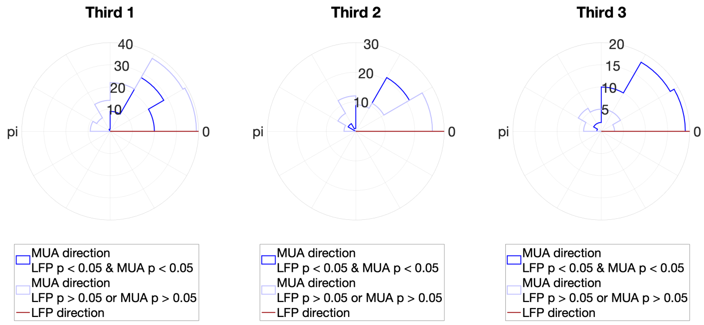

Supplementary Figure 68 Patient 2, array 1, seizure 1, relationship between MUA and LFP direction. Seizure bursts were divided into thirds (*left*: first third; *middle*: middle third, *right*: last third). For each third, we provided the distribution of burst-wise LFP-to-MUA directions. For each burst, LFP and MUA direction are compared, and the absolute value of the angular difference is put into the histogram. Bursts are divided into those with LFP and MUA p-value both less than 0.05 (*dark blue* histogram), and those with either MUA or LFP with p-value greater than 0.05. (*light blue* histogram).

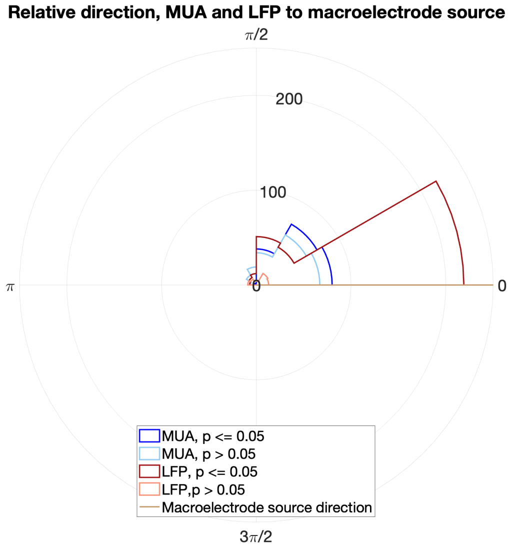

Supplementary Figure 69 Patient 2, array 1, seizure 1, relationship between discharge source, as determined by macroelectrode recordings, and direction of burst MUA and LFP. We used the time differences of the signal recorded in macroelectrodes to determine the location of the discharge source (Diamond, et al 2021, Diamond, et al 2023, see Supplementary Figure 12). The shortest path from the source to the array was then obtained, and we measured the angle of approach of the shortest path to the array (*tan* line). We then determined the relative direction of MUA and LFP signal, for each burst, to the discharge source as measured by macroelectrodes. Histograms are then provided, for the absolute value of the relative direction between source and MUA sequences (*blue*) and source and LFP sequences (*red*), for significant sequences (*dark* colors) and non-significant sequences (*light* colors).

Seizure activity, seizure 2

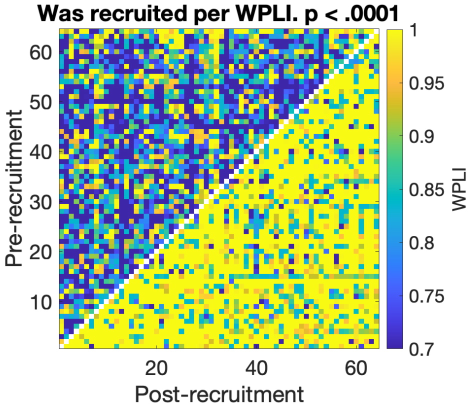

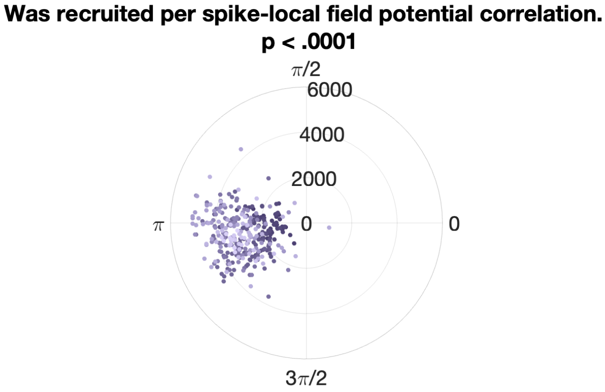

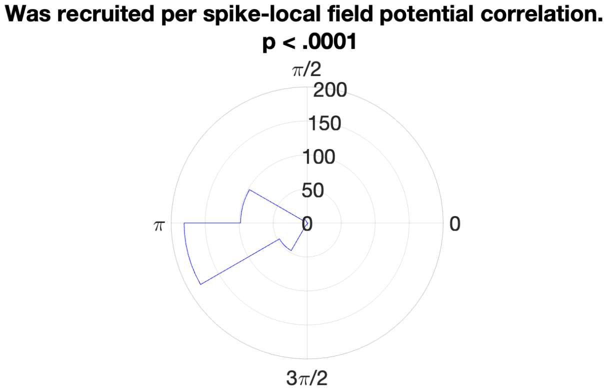

Supplementary Figure 70 Measures of recruitment for patient 2, array 1, seizure 2. Top: weighted phase lag index (WPLI), 10 seconds prior to seizure onset (top left), and 10 seconds after recruitment (bottom right, see *Methods*). Bottom left: relationship between LFP phase (θ) and LFP phase power (ρ), over the course of the seizure. *Dark purple* dots: early seizure; *light purple* dots: late seizure. Bottom right: distribution of spike-local field correlation values.

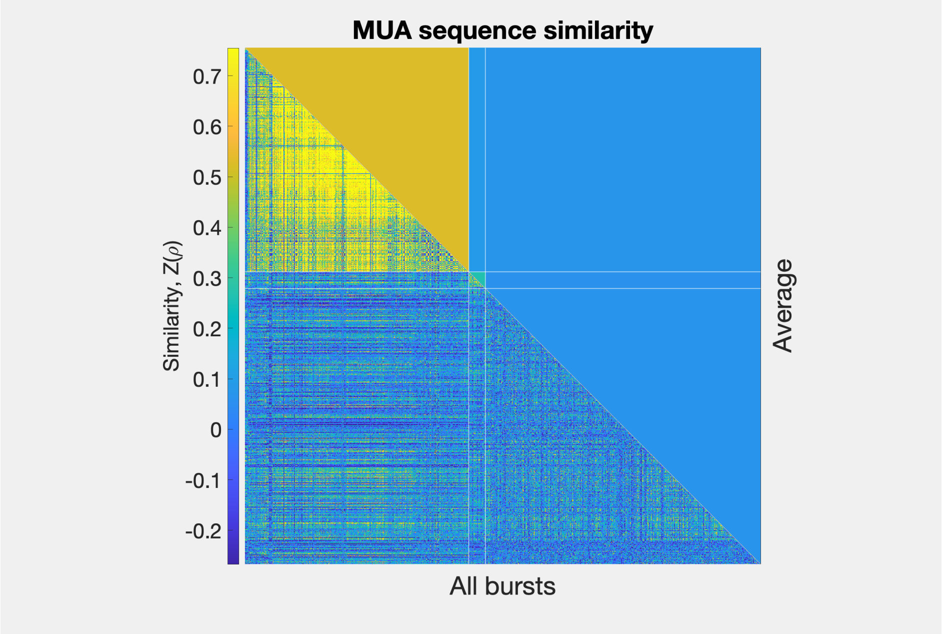

Supplementary Figure 71 Patient 2, array 1, seizure 2, burst similarity. For this patient, array, and seizure, every sequence was compared to every other sequence. Each cell indicates the similarity of the row sequence to the column sequence (Spearman's ρ). Sequences are divided by *white* lines into seizure sequences (*left, top*), IED sequences (*middle*), and baseline sequences (*right, bottom*). Actual values are provided in the lower triangle. In the upper triangle, average values are provided for each group, with values represented by the same color axis.

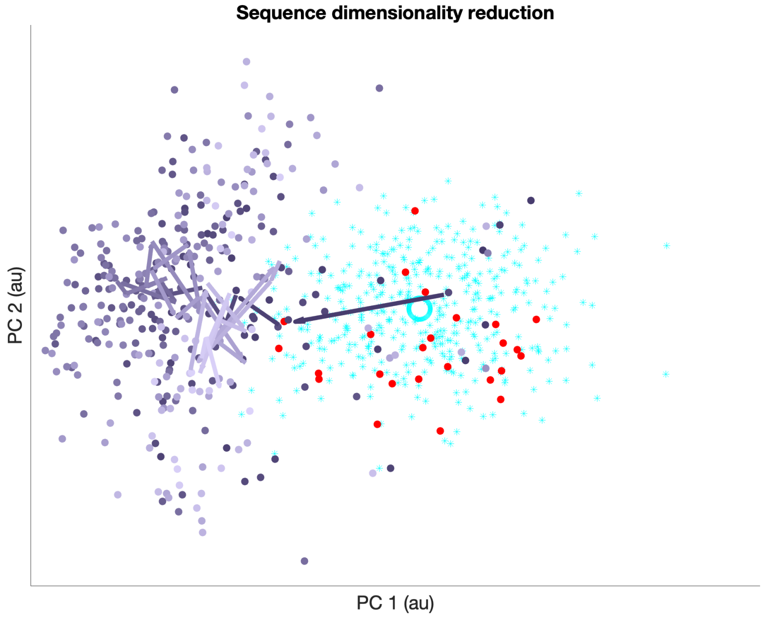

Supplementary Figure 72 Patient 2, array 1, seizure 2, dimensionality reduction using principal component analysis (PCA) was performed. We used PCA to decompose high-dimensional data into two dimensions in an unsupervised fashion (see *Methods*). Data provided include baseline bursts (*cyan stars*), IED bursts (*red dots*), and seizure bursts (*colored dots*; *dark purple*, early seizure, *light purple*, late seizure). The baseline centroid is indicated by the *cyan circle*.

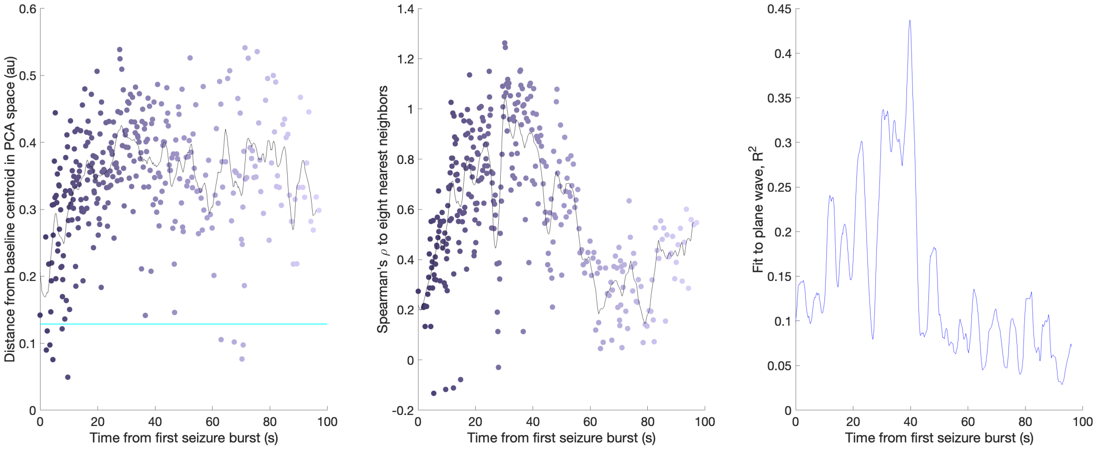

Supplementary Figure 73 Patient 2, array 1, seizure 2, measures of wave entrainment over time. *Left*: We used dimensionality reduction to collapse high-dimensional sequence information into two dimensions (see Figure 2a). We then took the location of the baseline centroid, and captured the distance, over time, between seizure bursts and the baseline centroid, in the low-dimensional manifold (see Figure 2c). *Dark purple* dots: early seizure; *light purple* dots: late seizure. A three-second moving average is superimposed (*black line*). Mean distance of baseline bursts to the baseline centroid is indicated by the *cyan line*. *Middle*: we captured Spearman's ρ between each seizure burst and its eight nearest temporal neighbors (see Figure 3a, b). This can be thought of as a measure of consistency of seizure bursts to each other in time. A three-second moving average of the ρ values is shown (*black line*). *Right*: for each burst, the R^2 value was determined from spatial linear regression (see *Methods*, section **Directionality of spike bursts and LFP discharges**). R^2 is a measure of the extent to which a burst is directional. A three-second moving average of the R^2 values is shown (*blue line*).

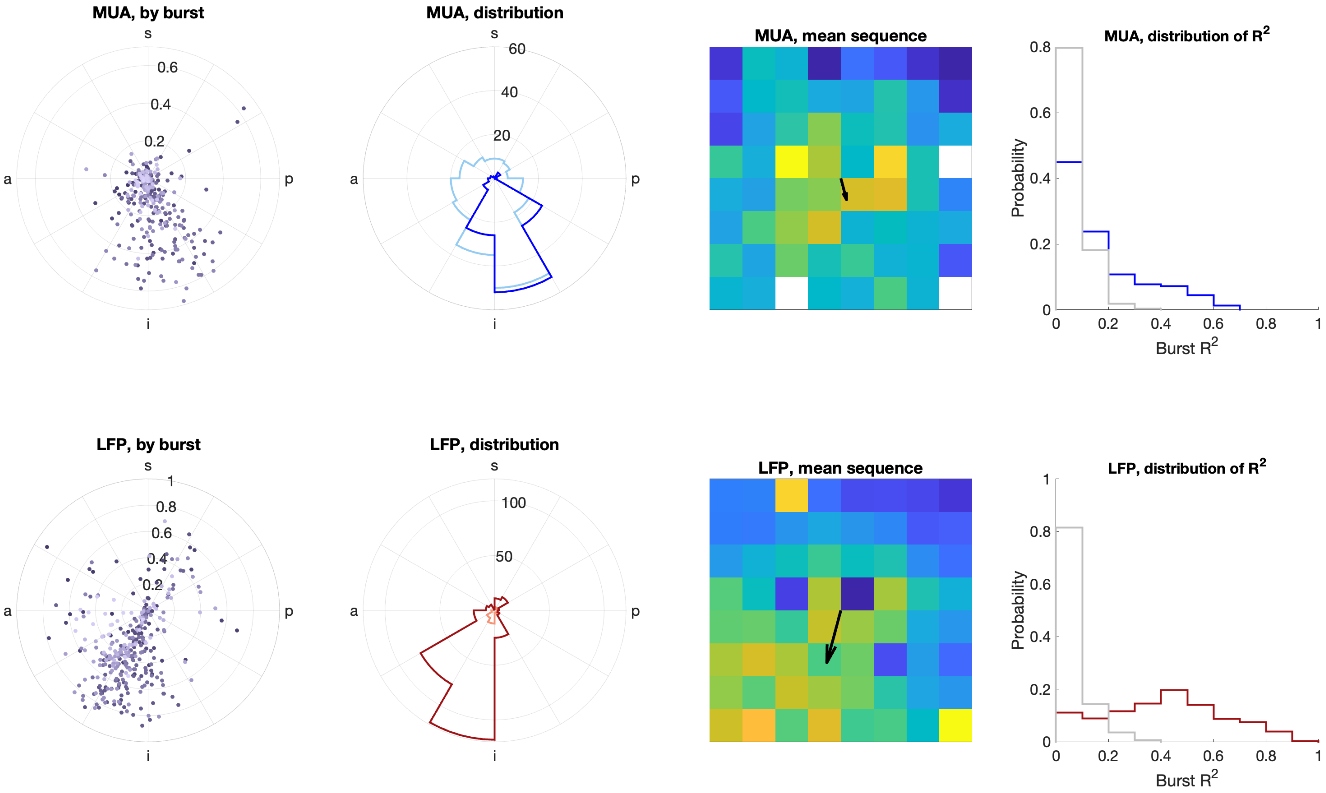

Supplementary Figure 74 Patient 2, array 1, seizure 2, seizure directionality. Top row: LFP. Bottom row: MUA. *Blue* histograms are used to designated MUA, while *maroon* histograms are used to designate LFP. First column from left: each dot represents a burst. θ reflects direction, and ρ is R^2, a measure of goodness of fit of the data to a plane. In other words, it is a measure of directionality of the burst. Second column from left: distribution direction of all recorded bursts. *Dark colors* indicate bursts with spatial linear regression giving $p \leq 0.05$, while *light* colors indicate bursts with $p > 0.05$. Third column from left: the average (*backbone*) sequence is shown over all bursts. This is obtained by taking the mean normalized rank of all sequences. The length of the *black arrow* indicates the mean R^2 over all bursts of this type, while the angle of the arrow indicates the mode of the histogram in the *second from left* column, for bursts with $p \leq 0.05$. Fourth column from left: R^2 for all bursts is shown. *Colored* histogram indicates the true sequences. *Gray* histogram, on the other hand, indicates R^2 for a set of null sequences, created by shuffling the ranks of the true sequences.

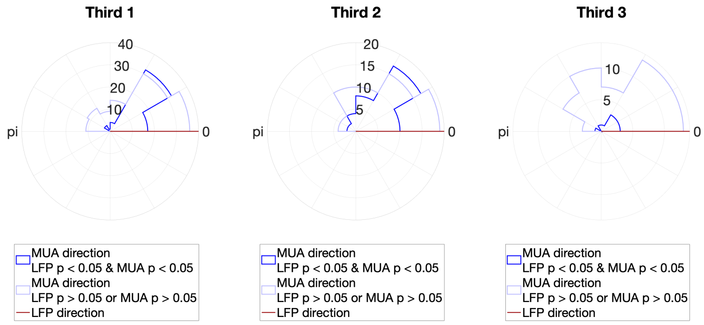

Supplementary Figure 75 Patient 2, array 1, seizure 2, relationship between MUA and LFP direction. Seizure bursts were divided into thirds (*left*: first third; *middle*: middle third, *right*: last third). For each third, we provided the distribution of burst-wise LFP-to-MUA directions. For each burst, LFP and MUA direction are compared, and the absolute value of the angular difference is put into the histogram. Bursts are divided into those with LFP and MUA p-value both less than 0.05 (*dark blue* histogram), and those with either MUA or LFP with p-value greater than 0.05. (*light blue* histogram).

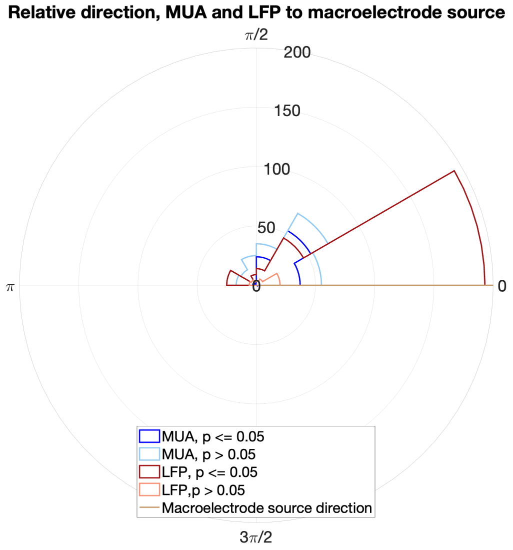

Supplementary Figure 76 Patient 2, array 1, seizure 2, relationship between discharge source, as determined by macroelectrode recordings, and direction of burst MUA and LFP. We used the time differences of the signal recorded in macroelectrodes to determine the location of the discharge source (Diamond, et al 2021, Diamond, et al 2023, see Supplementary Figure 12). The shortest path from the source to the array was then obtained, and we measured the angle of approach of the shortest path to the array (*tan* line). We then determined the relative direction of MUA and LFP signal, for each burst, to the discharge source as measured by macroelectrodes. Histograms are then provided, for the absolute value of the relative direction between source and MUA sequences (*blue*) and source and LFP sequences (*red*), for significant sequences (*dark* colors) and non-significant sequences (*light* colors).

Patient 2, array 2

Baseline activity

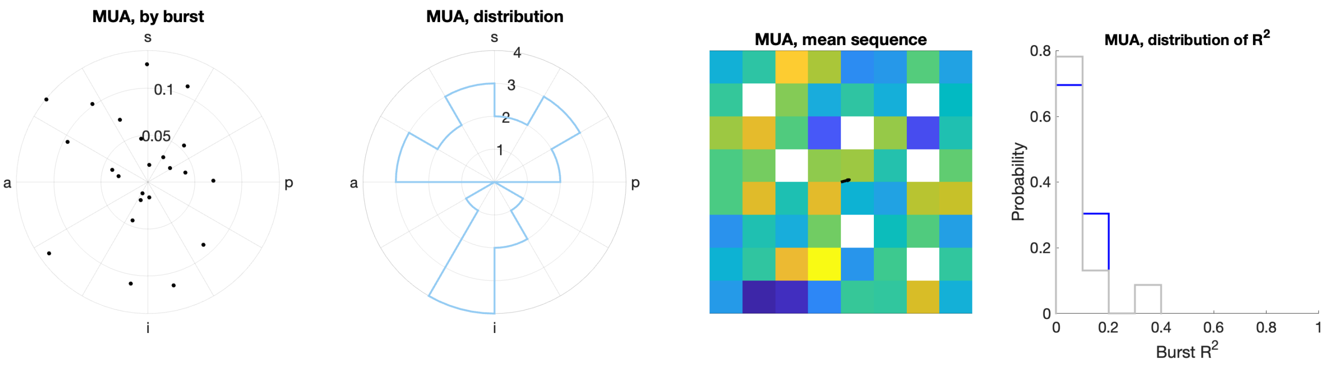

Supplementary Figure 77 Patient 2, array 2, baseline wavelike properties. First panel from left: each dot represents a burst. θ reflects direction, and ρ is R^2, a measure of goodness of fit of the data to a plane. In other words, it is a measure of the 'wavelike-ness' of the burst. Second panel from left: distribution direction of all recorded bursts. *Dark* colors indicate bursts with spatial linear regression giving $p \leq 0.05$, while *light* colors indicate bursts with $p > 0.05$. Third panel from left: the average (*backbone*) sequence is shown over all bursts. This is obtained by taking the mean normalized rank of all sequences. The length of the *black arrow* indicates the mean R^2 over all bursts of this type, while the angle of the arrow indicates the mode of the histogram in the *second from left* panel, for bursts with $p \leq 0.05$. Fourth panel from left: R^2 for all bursts is shown. *Blue* histogram indicates the true sequences. *Gray* histogram, on the other hand, indicates R^2 for a set of null sequences, created by shuffling the ranks of the true sequences.

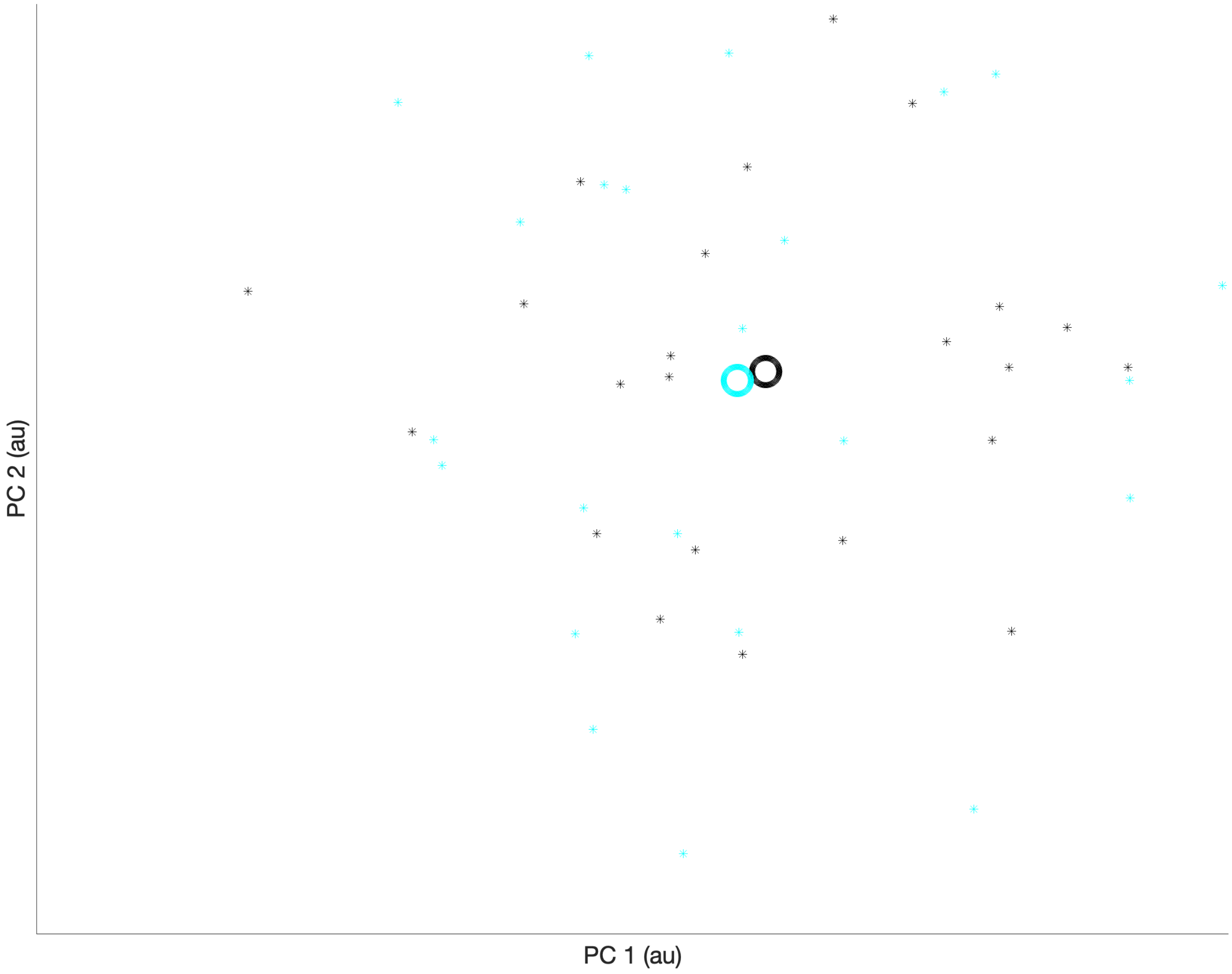

Supplementary Figure 78 Patient 2, array 2, baseline bursts versus random bursts. We were interested in determining that our baseline bursts were non-random. Therefore, for each baseline burst, we created a null counterpart, in which the MUA timings were shuffled only among spiking electrodes. We then applied both the true baselines and the random counterparts to the same dimensionality reduction procedure. The baseline centroid is indicated by the *blue circle*. The random centroid indicated by the *black* circle. For most patients and arrays, baseline bursts are displaced from the random bursts. Distance from baseline bursts to the random centroid tends to be greater than distance from random bursts to the random centroid (Supplementary Figure 4).

IED activity

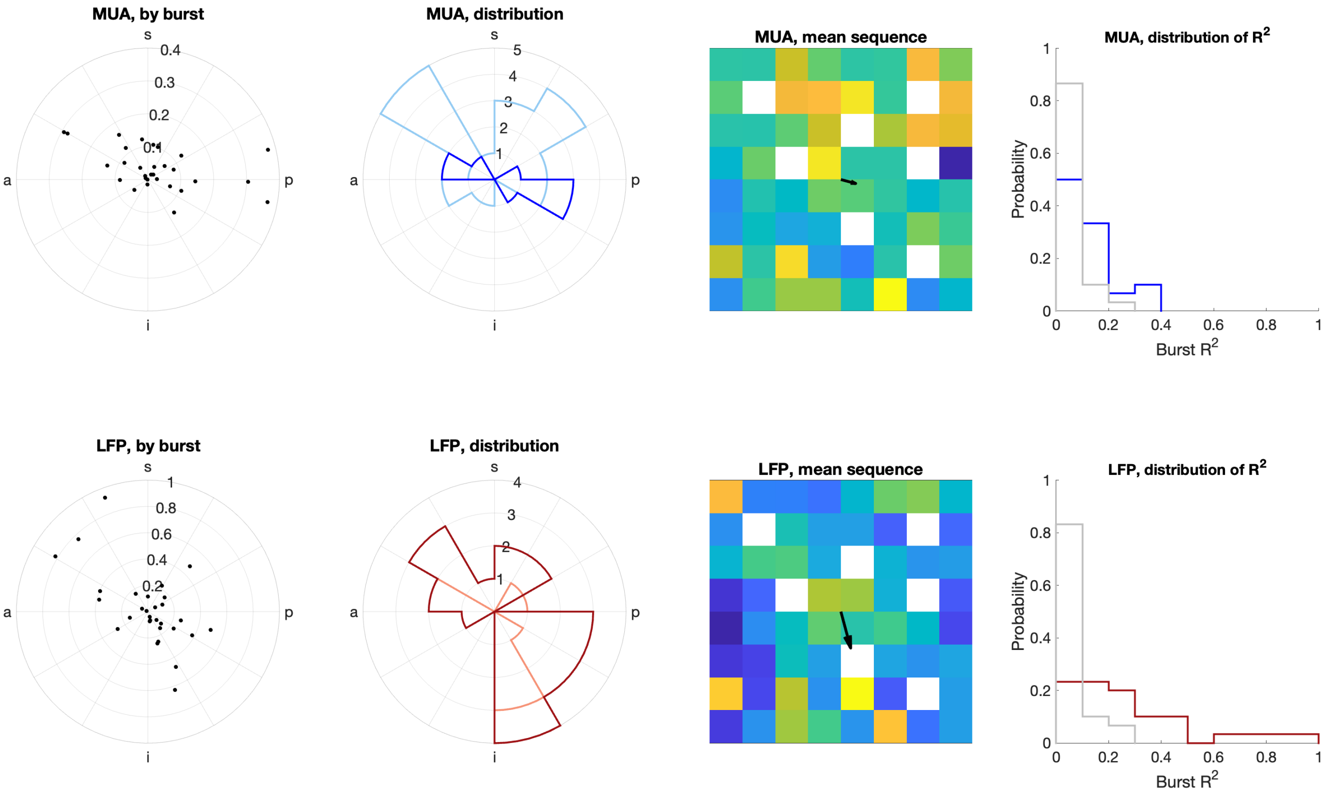

Supplementary Figure 79 Patient 2, array 2, IED directionality. Top row: LFP. Bottom row: MUA. *Dark blue* histograms are used to designated MUA, while *maroon* histograms are used to designate LFP. First column from left: each dot represents a burst. θ reflects direction, and ρ is R^2, a measure of goodness of fit of the data to a plane. In other words, it is a measure of the strength of directionality of the burst. Second column from left: distribution direction of all recorded bursts. *Dark colors* indicate bursts with spatial linear regression giving $p \leq 0.05$, while *light* colors indicate bursts with $p > 0.05$. Third column from left: the average (*backbone*) sequence is shown over all bursts for this seizure. This is obtained by taking the mean normalized rank of all sequences. The length of the *black arrow* indicates the mean R^2 over all bursts of this type, while the angle of the arrow indicates the mode of the histogram in the *second from left* column, for bursts with $p \leq 0.05$. Fourth column from left: R^2 for all bursts is shown. *Colored* histogram indicates the true sequences. *Gray* histogram, on the other hand, indicates R^2 for a set of null sequences, created by shuffling the ranks of spiking electrodes in the true sequences.

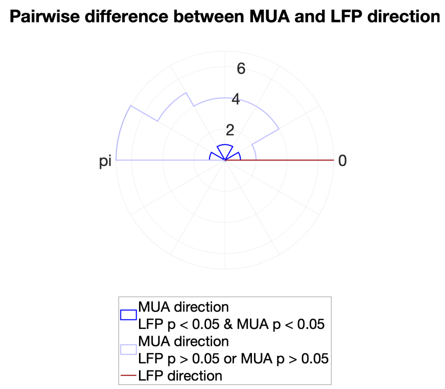

Supplementary Figure 80 Patient 2, array 2, IED relationship between MUA and LFP direction. Distribution of pairwise LFP-to-MUA direction. For each burst, LFP and MUA direction are compared, and the absolute value of the angular difference is put into the histogram. Bursts are divided into those with LFP and MUA p-value both less than 0.05, or those with either MUA or LFP with p-value greater than 0.05.

Seizure activity, seizure 1

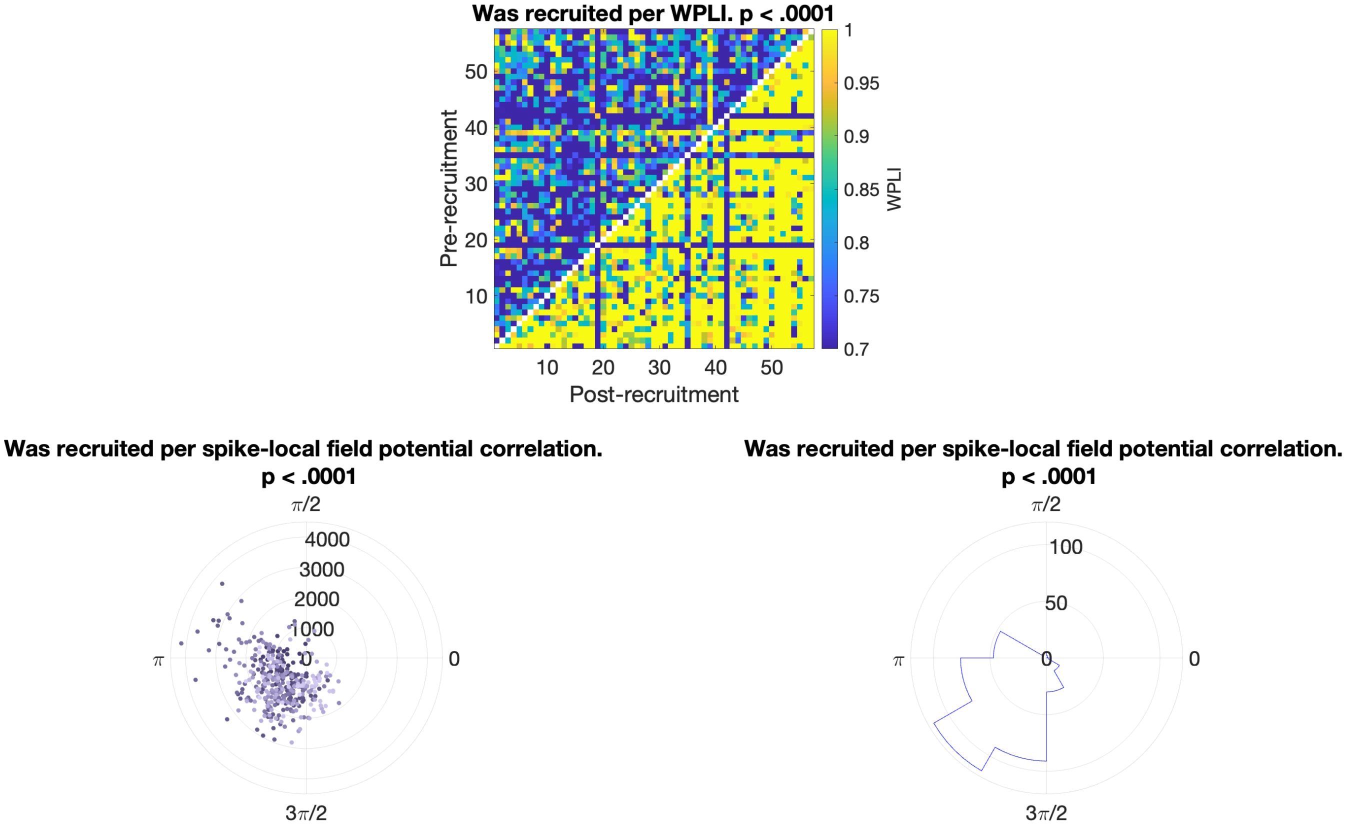

Supplementary Figure 81 Measures of recruitment for patient 2, array 2, seizure 1. Top: weighted phase lag index (WPLI), 10 seconds prior to seizure onset (top left), and 10 seconds after recruitment (bottom right, see *Methods*). Bottom left: relationship between LFP phase (θ) and LFP phase power (ρ), over the course of the seizure. *Dark purple* dots: early seizure; *light purple* dots: late seizure. Bottom right: distribution of spike-local field correlation values.

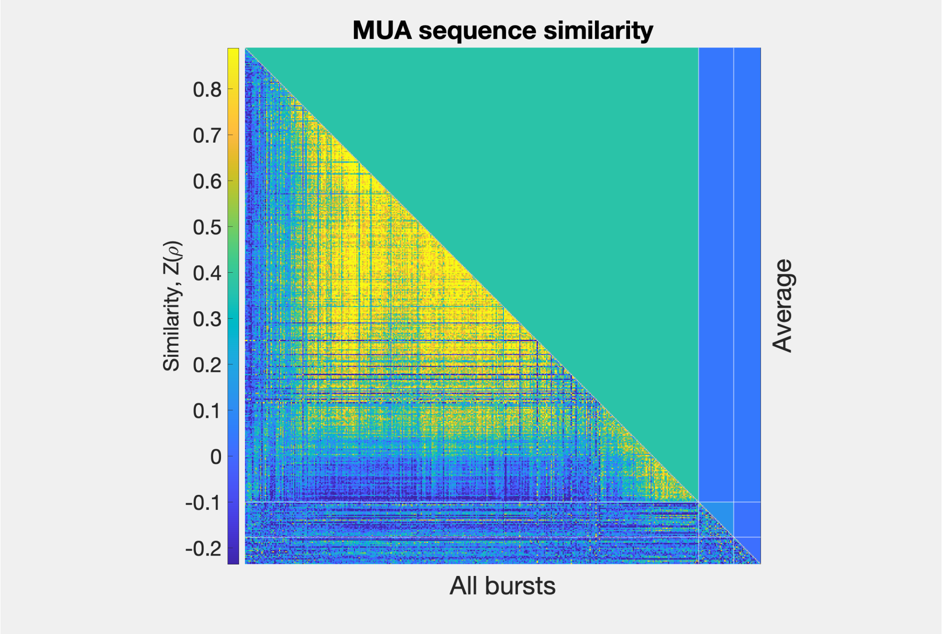

Supplementary Figure 82 Patient 2, array 2, seizure 1, burst similarity. For this patient, array, and seizure, every sequence was compared to every other sequence. Each cell indicates the similarity of the row sequence to the column sequence (Spearman's ρ). Sequences are divided by *white* lines into seizure sequences (*left, top*), IED sequences (*middle*), and baseline sequences (*right, bottom*). Actual values are provided in the lower triangle. In the upper triangle, average values are provided for each group, with values represented by the same color axis.

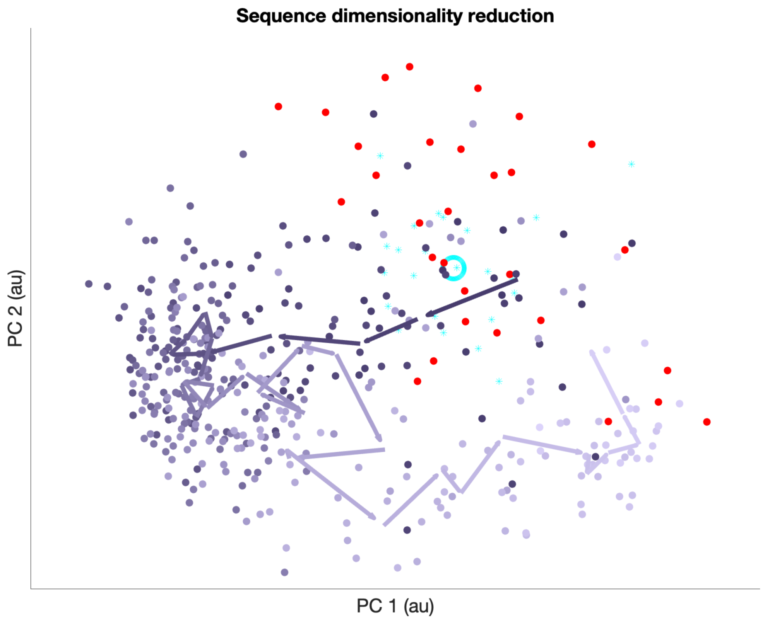

Supplementary Figure 83 Patient 2, array 2, seizure 1, dimensionality reduction using principal component analysis (PCA) was performed. We used PCA to decompose high-dimensional data into two dimensions in an unsupervised fashion (see *Methods*). Data provided include baseline bursts (*cyan stars*), IED bursts (*red dots*), and seizure bursts (*colored dots*; *dark purple*, early seizure, *light purple*, late seizure). The baseline centroid is indicated by the *cyan circle*.

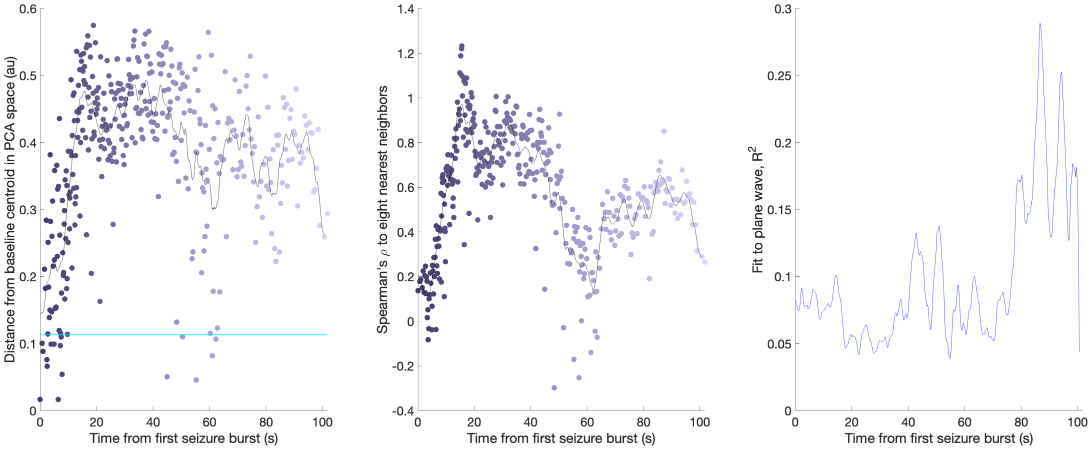

Supplementary Figure 84 Patient 2, array 2, seizure 1, measures of wave entrainment over time. *Left*: We used dimensionality reduction to collapse high-dimensional sequence information into two dimensions (see Figure 2a). We then took the location of the baseline centroid, and captured the distance, over time, between seizure bursts and the baseline centroid, in the low-dimensional manifold (see Figure 2c). *Dark purple* dots: early seizure; *light purple* dots: late seizure. A three-second moving average is superimposed (*black line*). Mean distance of baseline bursts to the baseline centroid is indicated by the *cyan line*. *Middle*: we captured Spearman's ρ between each seizure burst and its eight nearest temporal neighbors (see Figure 3a, b). This can be thought of as a measure of consistency of seizure bursts to each other in time. A three-second moving average of the ρ values is shown (*black line*). *Right*: for each burst, the R^2 value was determined from spatial linear regression (see *Methods*, section **Directionality of spike bursts and LFP discharges**). R^2 is a measure of the extent to which a burst is directional. A three-second moving average of the R^2 values is shown (*blue line*).

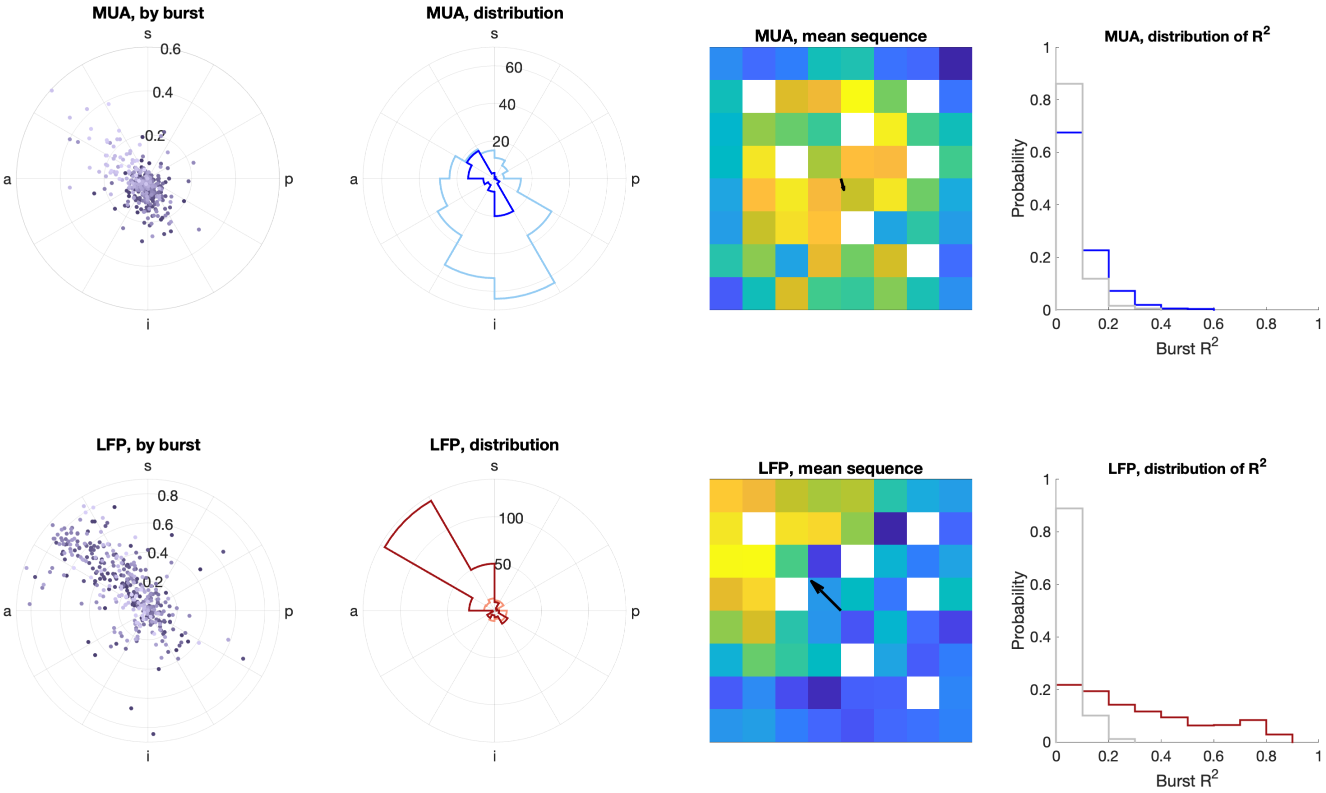

Supplementary Figure 85 Patient 2, array 2, seizure 1, seizure directionality. Top row: LFP. Bottom row: MUA. *Blue* histograms are used to designated MUA, while *maroon* histograms are used to designate LFP. First column from left: each dot represents a burst. θ reflects direction, and ρ is R^2, a measure of goodness of fit of the data to a plane. In other words, it is a measure of directionality of the burst. Second column from left: distribution direction of all recorded bursts. *Dark colors* indicate bursts with spatial linear regression giving $p \leq 0.05$, while *light* colors indicate bursts with $p > 0.05$. Third column from left: the average (*backbone*) sequence is shown over all bursts. This is obtained by taking the mean normalized rank of all sequences. The length of the *black arrow* indicates the mean R^2 over all bursts of this type, while the angle of the arrow indicates the mode of the histogram in the *second from left* column, for bursts with $p \leq 0.05$. Fourth column from left: R^2 for all bursts is shown. *Colored* histogram indicates the true sequences. *Gray* histogram, on the other hand, indicates R^2 for a set of null sequences, created by shuffling the ranks of the true sequences.

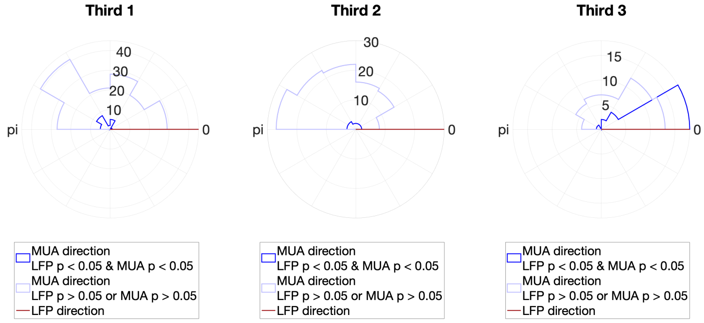

Supplementary Figure 86 Patient 2, array 2, seizure 1, relationship between MUA and LFP direction. Seizure bursts were divided into thirds (*left*: first third; *middle*: middle third, *right*: last third). For each third, we provided the distribution of burst-wise LFP-to-MUA directions. For each burst, LFP and MUA direction are compared, and the absolute value of the angular difference is put into the histogram. Bursts are divided into those with LFP and MUA p-value both less than 0.05 (*dark blue* histogram), and those with either MUA or LFP with p-value greater than 0.05. (*light blue* histogram).

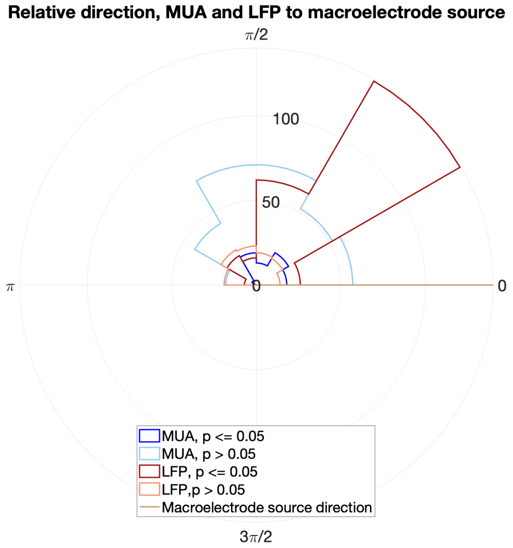

Supplementary Figure 87 Patient 2, array 2, seizure 1, relationship between discharge source, as determined by macroelectrode recordings, and direction of burst MUA and LFP. We used the time differences of the signal recorded in macroelectrodes to determine the location of the discharge source (Diamond, et al 2021, Diamond, et al 2023, see Supplementary Figure 12). The shortest path from the source to the array was then obtained, and we measured the angle of approach of the shortest path to the array (*tan* line). We then determined the relative direction of MUA and LFP signal, for each burst, to the discharge source as measured by macroelectrodes. Histograms are then provided, for the absolute value of the relative direction between source and MUA sequences (*blue*) and source and LFP sequences (*red*), for significant sequences (*dark* colors) and non-significant sequences (*light* colors).

Seizure activity, seizure 2

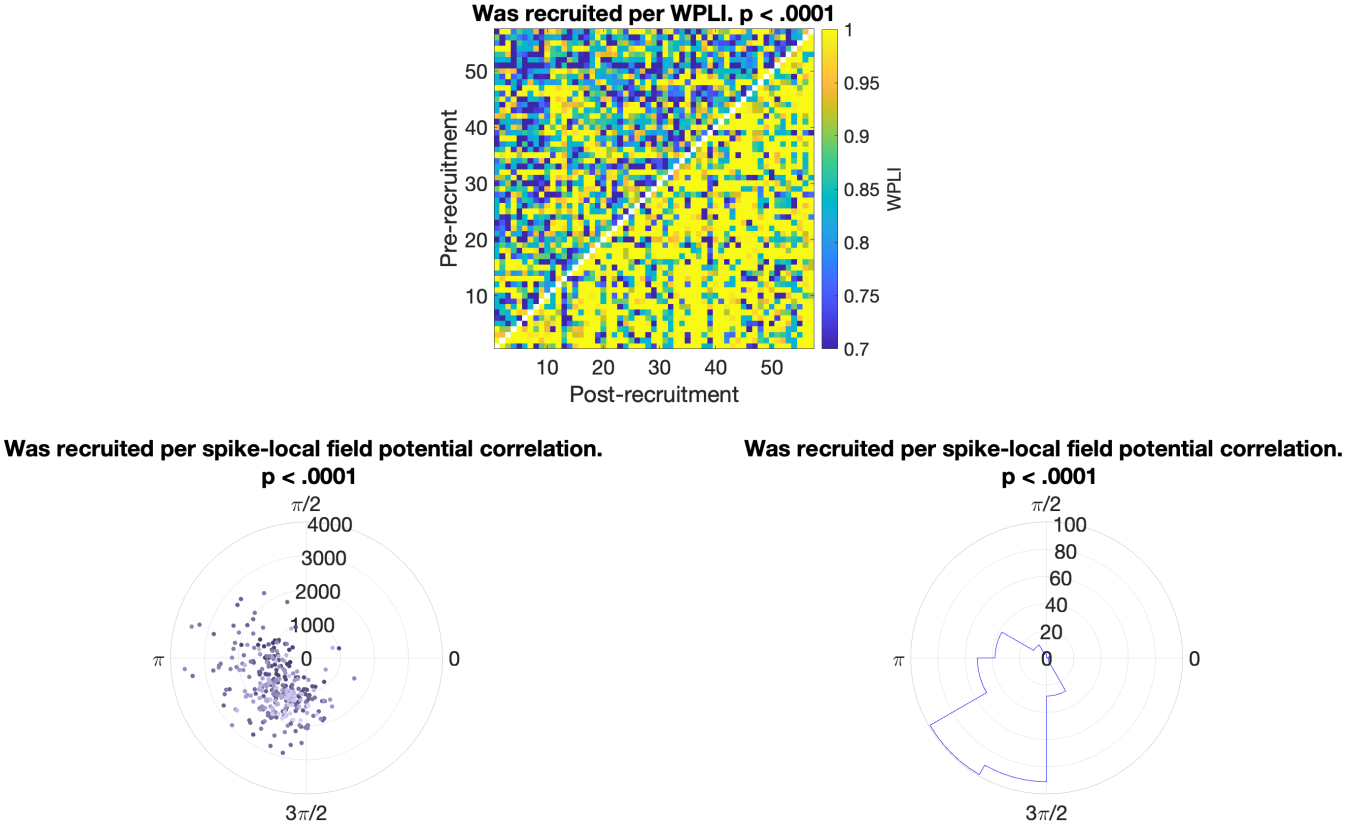

Supplementary Figure 88 Measures of recruitment for patient 2, array 2, seizure 2. Top: weighted phase lag index (WPLI), 10 seconds prior to seizure onset (top left), and 10 seconds after recruitment (bottom right, see *Methods*). Bottom left: relationship between LFP phase (θ) and LFP phase power (ρ), over the course of the seizure. *Dark purple* dots: early seizure; *light purple* dots: late seizure. Bottom right: distribution of spike-local field correlation values.

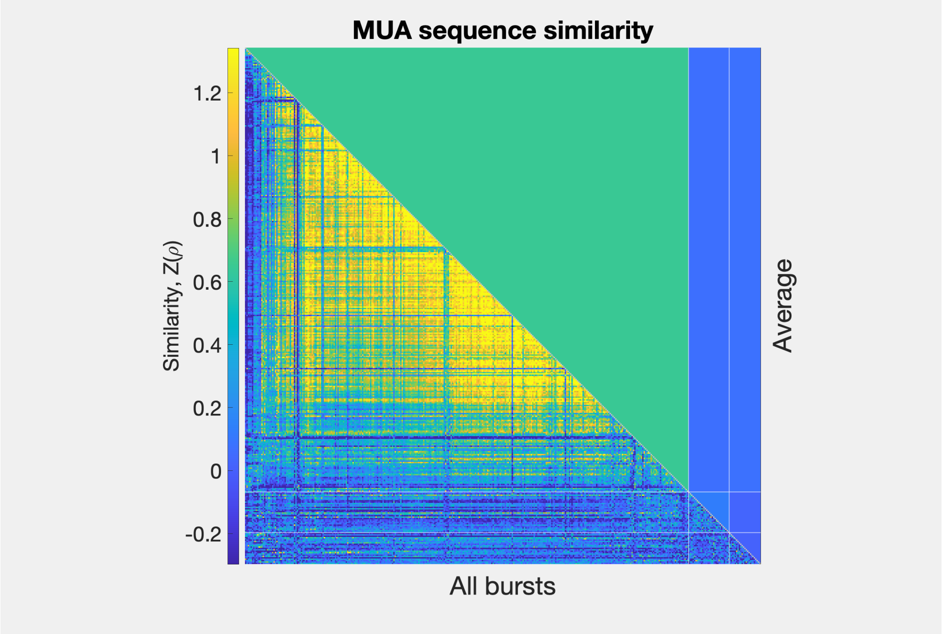

Supplementary Figure 89 Patient 2, array 2, seizure 2, burst similarity. For this patient, array, and seizure, every sequence was compared to every other sequence. Each cell indicates the similarity of the row sequence to the column sequence (Spearman's ρ). Sequences are divided by *white* lines into seizure sequences (*left, top*), IED sequences (*middle*), and baseline sequences (*right, bottom*). Actual values are provided in the lower triangle. In the upper triangle, average values are provided for each group, with values represented by the same color axis.

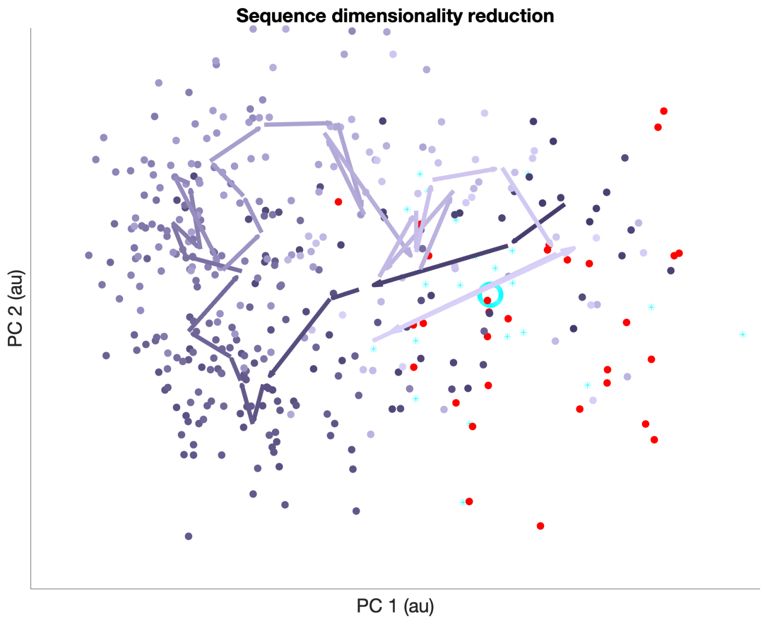

Supplementary Figure 90 Patient 2, array 2, seizure 2, dimensionality reduction using principal component analysis (PCA) was performed. We used PCA to decompose high-dimensional data into two dimensions in an unsupervised fashion (see *Methods*). Data provided include baseline bursts (*cyan stars*), IED bursts (*red dots*), and seizure bursts (*colored dots*; *dark purple*, early seizure, *light purple*, late seizure). The baseline centroid is indicated by the *cyan circle*.

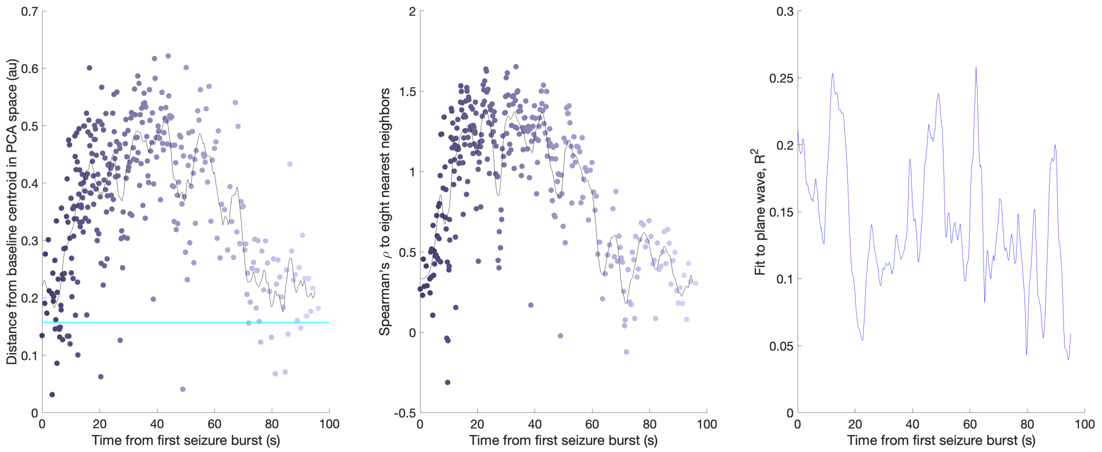

Supplementary Figure 91 Patient 2, array 2, seizure 2, measures of wave entrainment over time. *Left*: We used dimensionality reduction to collapse high-dimensional sequence information into two dimensions (see Figure 2a). We then took the location of the baseline centroid, and captured the distance, over time, between seizure bursts and the baseline centroid, in the low-dimensional manifold (see Figure 2c). *Dark purple* dots: early seizure; *light purple* dots: late seizure. A three-second moving average is superimposed (*black line*). Mean distance of baseline bursts to the baseline centroid is indicated by the *cyan line*. *Middle*: we captured Spearman's ρ between each seizure burst and its eight nearest temporal neighbors (see Figure 3a, b). This can be thought of as a measure of consistency of seizure bursts to each other in time. A three-second moving average of the ρ values is shown (*black line*). *Right*: for each burst, the R^2 value was determined from spatial linear regression (see *Methods*, section **Directionality of spike bursts and LFP discharges**). R^2 is a measure of the extent to which a burst is directional. A three-second moving average of the R^2 values is shown (*blue line*).

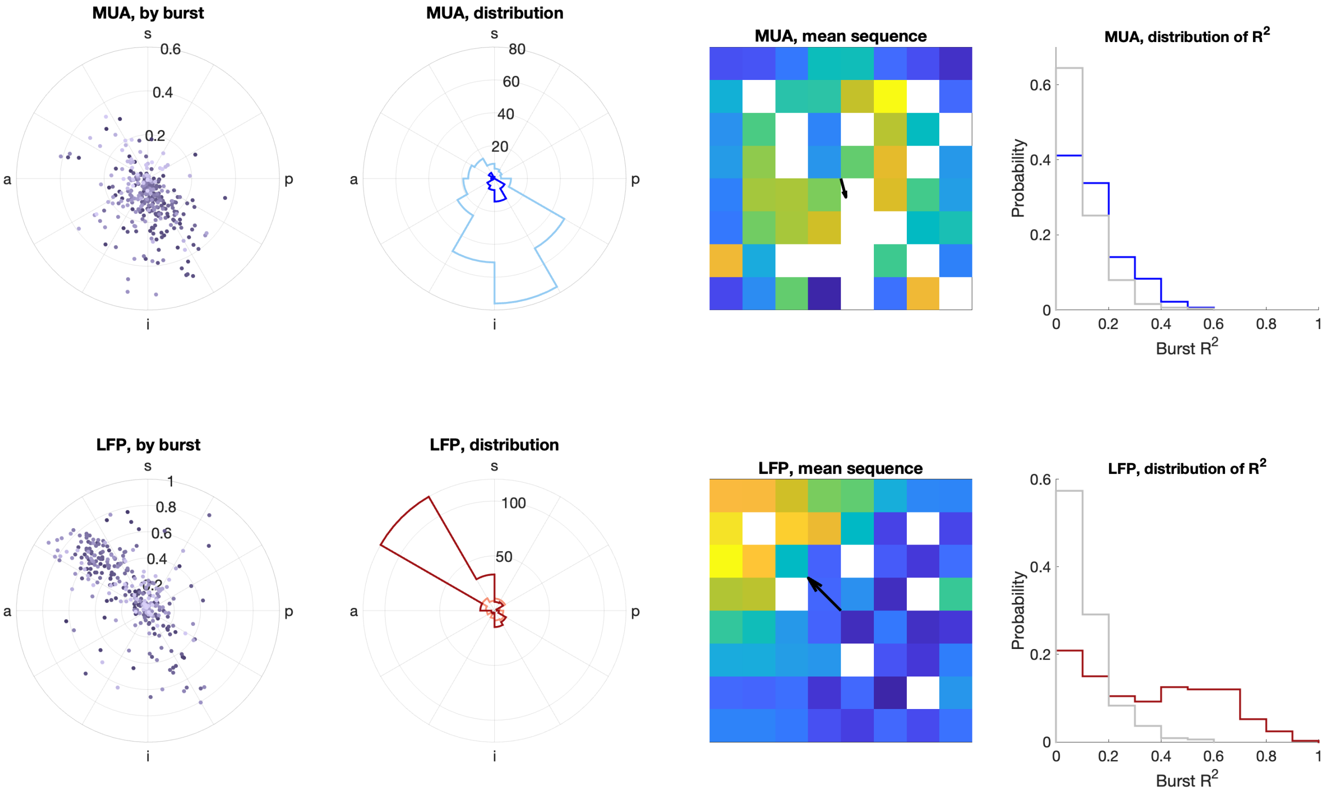

Supplementary Figure 92 Patient 2, array 2, seizure 2, seizure directionality. Top row: LFP. Bottom row: MUA. *Blue* histograms are used to designated MUA, while *maroon* histograms are used to designate LFP. First column from left: each dot represents a burst. θ reflects direction, and ρ is R^2, a measure of goodness of fit of the data to a plane. In other words, it is a measure of directionality of the burst. Second column from left: distribution direction of all recorded bursts. *Dark colors* indicate bursts with spatial linear regression giving $p \leq 0.05$, while *light* colors indicate bursts with $p > 0.05$. Third column from left: the average (*backbone*) sequence is shown over all bursts. This is obtained by taking the mean normalized rank of all sequences. The length of the *black arrow* indicates the mean R^2 over all bursts of this type, while the angle of the arrow indicates the mode of the histogram in the *second from left* column, for bursts with $p \leq 0.05$. Fourth column from left: R^2 for all bursts is shown. *Colored* histogram indicates the true sequences. *Gray* histogram, on the other hand, indicates R^2 for a set of null sequences, created by shuffling the ranks of the true sequences.

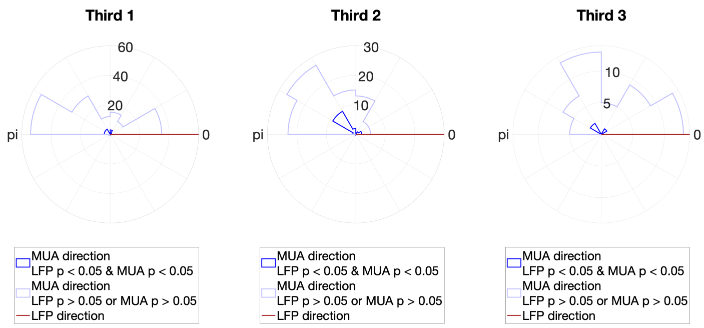

Supplementary Figure 93 Patient 2, array 2, seizure 2, relationship between MUA and LFP direction. Seizure bursts were divided into thirds (*left*: first third; *middle*: middle third, *right*: last third). For each third, we provided the distribution of burst-wise LFP-to-MUA directions. For each burst, LFP and MUA direction are compared, and the absolute value of the angular difference is put into the histogram. Bursts are divided into those with LFP and MUA p-value both less than 0.05 (*dark blue* histogram), and those with either MUA or LFP with p-value greater than 0.05. (*light blue* histogram).

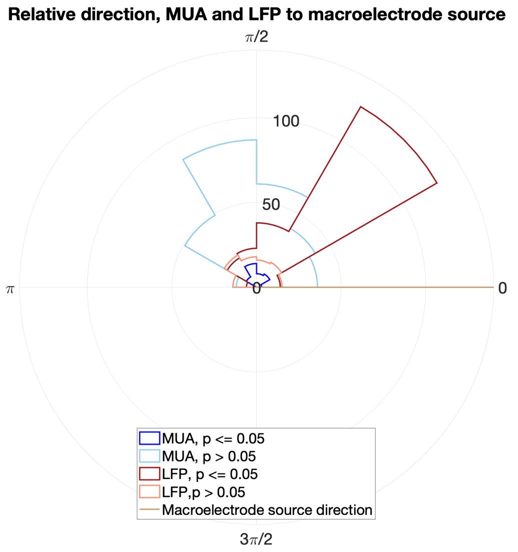

Supplementary Figure 94 Patient 2, array 2, seizure 2, relationship between discharge source, as determined by macroelectrode recordings, and direction of burst MUA and LFP. We used the time differences of the signal recorded in macroelectrodes to determine the location of the discharge source (Diamond, et al 2021, Diamond, et al 2023, see Supplementary Figure 12). The shortest path from the source to the array was then obtained, and we measured the angle of approach of the shortest path to the array (*tan* line). We then determined the relative direction of MUA and LFP signal, for each burst, to the discharge source as measured by macroelectrodes. Histograms are then provided, for the absolute value of the relative direction between source and MUA sequences (*blue*) and source and LFP sequences (*red*), for significant sequences (*dark* colors) and non-significant sequences (*light* colors).

Patient 3, array 1

Baseline activity

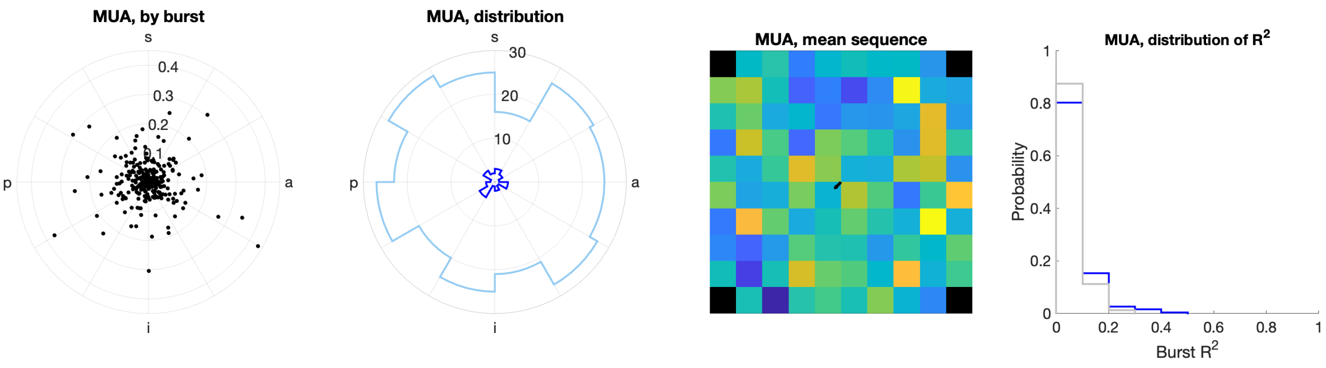

Supplementary Figure 95 Patient 3, array 1, baseline wavelike properties. First panel from left: each dot represents a burst. θ reflects direction, and ρ is R^2, a measure of goodness of fit of the data to a plane. In other words, it is a measure of the 'wavelike-ness' of the burst. Second panel from left: distribution direction of all recorded bursts. *Dark* colors indicate bursts with spatial linear regression giving $p \leq 0.05$, while *light* colors indicate bursts with $p > 0.05$. Third panel from left: the average (*backbone*) sequence is shown over all bursts. This is obtained by taking the mean normalized rank of all sequences. The length of the *black arrow* indicates the mean R^2 over all bursts of this type, while the angle of the arrow indicates the mode of the histogram in the *second from left* panel, for bursts with $p \leq 0.05$. Fourth panel from left: R^2 for all bursts is shown. *Blue* histogram indicates the true sequences. *Gray* histogram, on the other hand, indicates R^2 for a set of null sequences, created by shuffling the ranks of the true sequences.

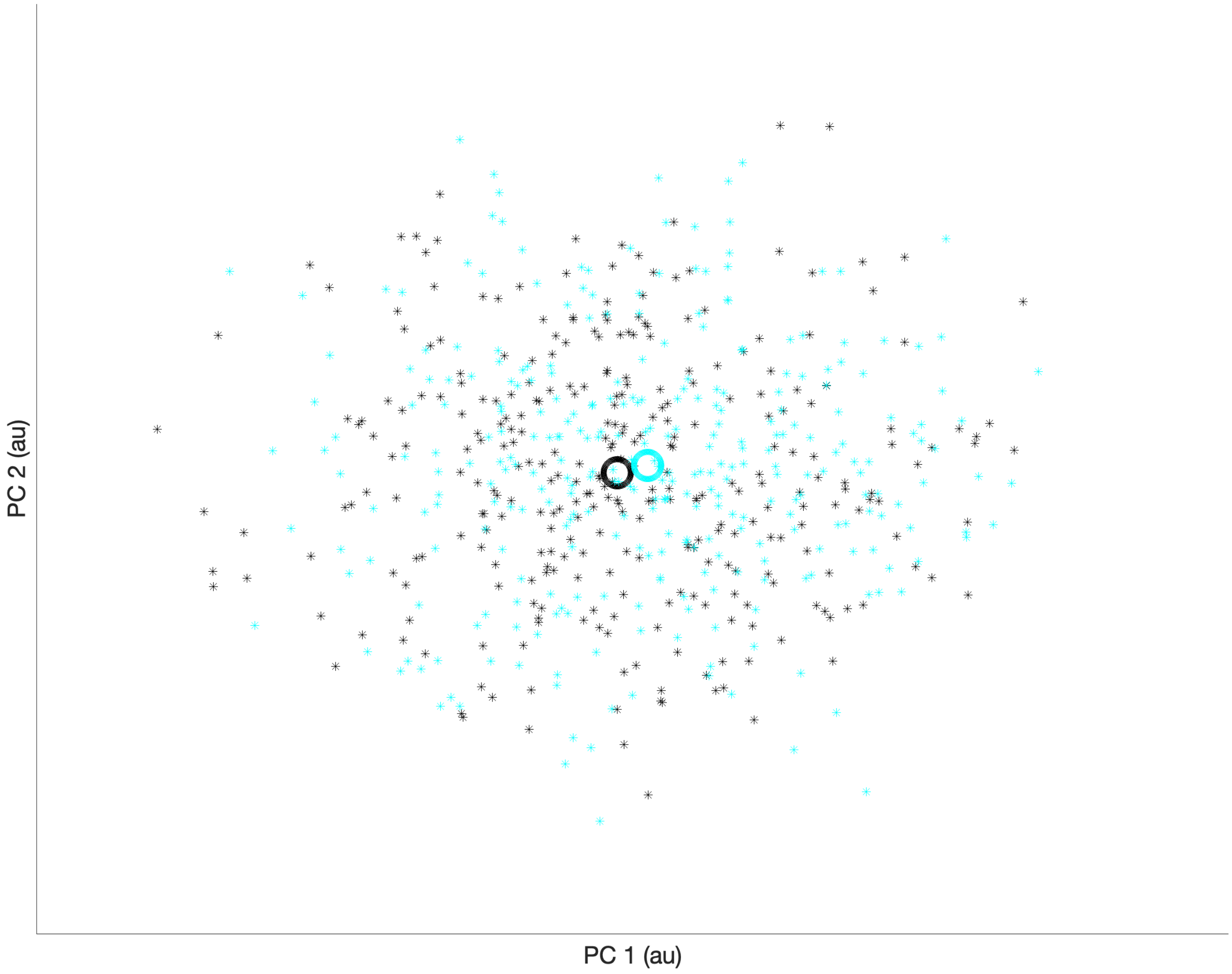

Supplementary Figure 96 Patient 3, array 1, baseline bursts versus random bursts. We were interested in determining that our baseline bursts were non-random. Therefore, for each baseline burst, we created a null counterpart, in which the MUA timings were shuffled only among spiking electrodes. We then applied both the true baselines and the random counterparts to the same dimensionality reduction procedure. The baseline centroid is indicated by the *blue circle*. The random centroid indicated by the *black* circle. For most patients and arrays, baseline bursts are displaced from the random bursts. Distance from baseline bursts to the random centroid tends to be greater than distance from random bursts to the random centroid (Supplementary Figure 4).

IED activity

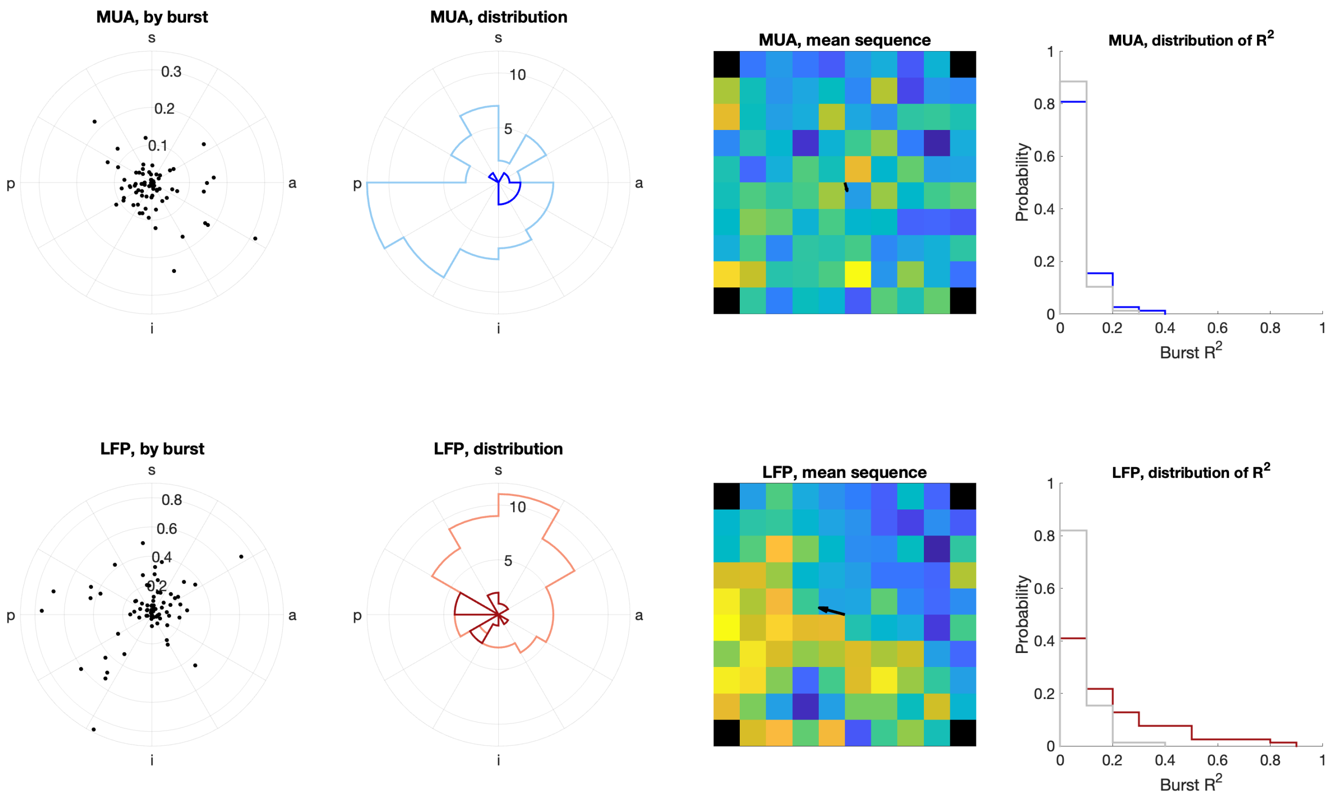

Supplementary Figure 97 Patient 3, array 1, IED directionality. Top row: LFP. Bottom row: MUA. *Dark blue* histograms are used to designated MUA, while *maroon* histograms are used to designate LFP. First column from left: each dot represents a burst. θ reflects direction, and ρ is R^2, a measure of goodness of fit of the data to a plane. In other words, it is a measure of the strength of directionality of the burst. Second column from left: distribution direction of all recorded bursts. *Dark colors* indicate bursts with spatial linear regression giving $p \leq 0.05$, while *light* colors indicate bursts with $p > 0.05$. Third column from left: the average (*backbone*) sequence is shown over all bursts for this seizure. This is obtained by taking the mean normalized rank of all sequences. The length of the *black arrow* indicates the mean R^2 over all bursts of this type, while the angle of the arrow indicates the mode of the histogram in the *second from left* column, for bursts with $p \leq 0.05$. Fourth column from left: R^2 for all bursts is shown. *Colored* histogram indicates the true sequences. *Gray* histogram, on the other hand, indicates R^2 for a set of null sequences, created by shuffling the ranks of spiking electrodes in the true sequences.

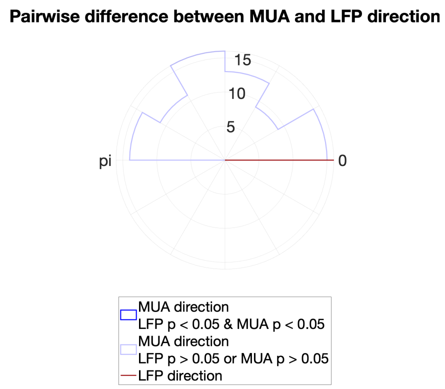

Supplementary Figure 98 Patient 3, array 1, IED relationship between MUA and LFP direction. Distribution of pairwise LFP-to-MUA direction. For each burst, LFP and MUA direction are compared, and the absolute value of the angular difference is put into the histogram. Bursts are divided into those with LFP and MUA p-value both less than 0.05, or those with either MUA or LFP with p-value greater than 0.05.

Seizure activity, seizure 1

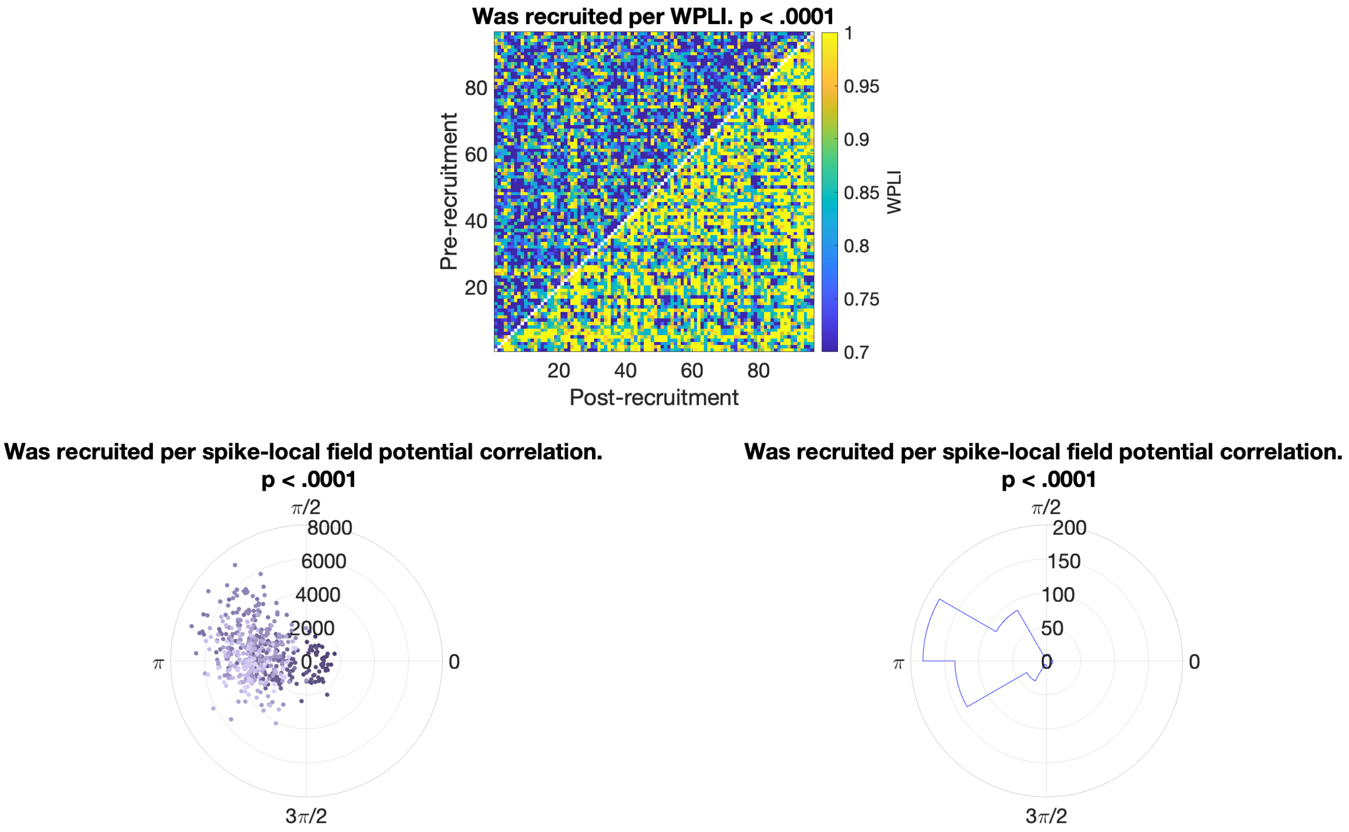

Supplementary Figure 99 Measures of recruitment for patient 3, array 1, seizure 1. Top: weighted phase lag index (WPLI), 10 seconds prior to seizure onset (top left), and 10 seconds after recruitment (bottom right, see *Methods*). Bottom left: relationship between LFP phase (θ) and LFP phase power (ρ), over the course of the seizure. *Dark purple* dots: early seizure; *light purple* dots: late seizure. Bottom right: distribution of spike-local field correlation values.

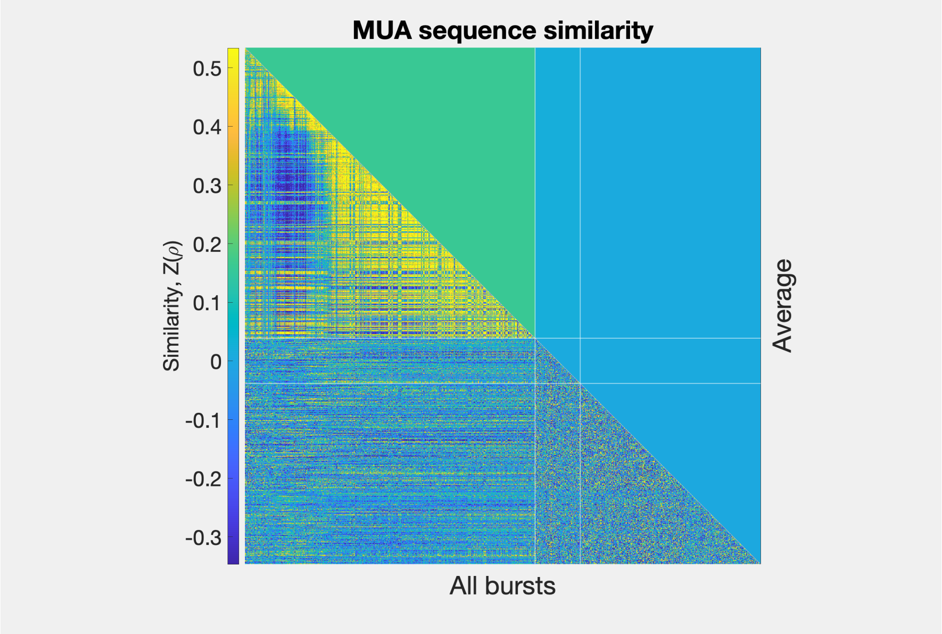

Supplementary Figure 100 Patient 3, array 1, seizure 1, burst similarity. For this patient, array, and seizure, every sequence was compared to every other sequence. Each cell indicates the similarity of the row sequence to the column sequence (Spearman's ρ). Sequences are divided by *white* lines into seizure sequences (*left, top*), IED sequences (*middle*), and baseline sequences (*right, bottom*). Actual values are provided in the lower triangle. In the upper triangle, average values are provided for each group, with values represented by the same color axis.

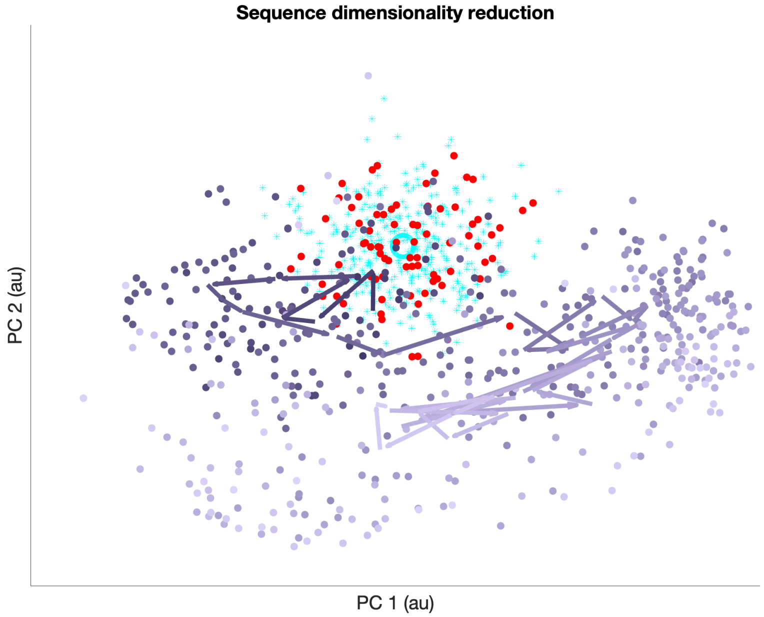

Supplementary Figure 101 Patient 3, array 1, seizure 1, dimensionality reduction using principal component analysis (PCA) was performed. We used PCA to decompose high-dimensional data into two dimensions in an unsupervised fashion (see *Methods*). Data provided include baseline bursts (*cyan stars*), IED bursts (*red dots*), and seizure bursts (*colored dots*; *dark purple*, early seizure, *light purple*, late seizure). The baseline centroid is indicated by the *cyan circle*.

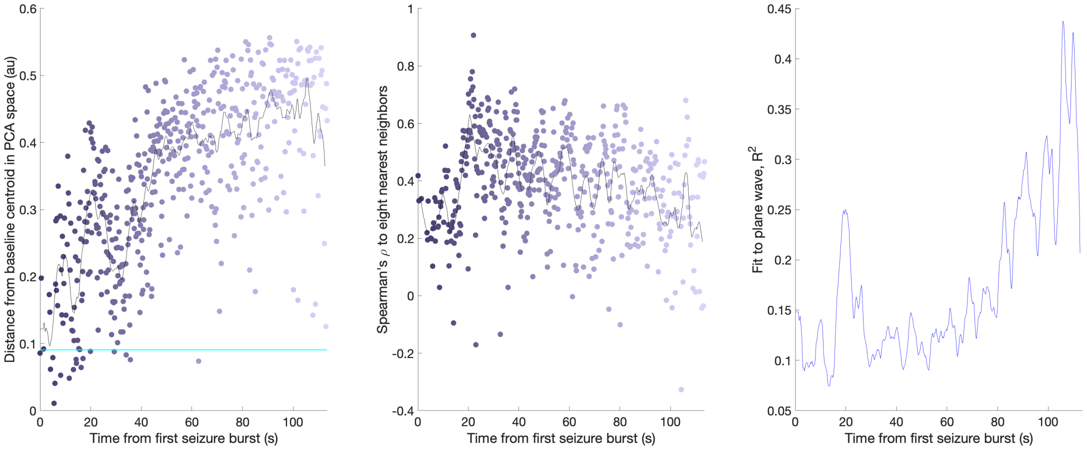

Supplementary Figure 102 Patient 3, array 1, seizure 1, measures of wave entrainment over time. *Left*: We used dimensionality reduction to collapse high-dimensional sequence information into two dimensions (see Figure 2a). We then took the location of the baseline centroid, and captured the distance, over time, between seizure bursts and the baseline centroid, in the low-dimensional manifold (see Figure 2c). *Dark purple* dots: early seizure; *light purple* dots: late seizure. A three-second moving average is superimposed (*black line*). Mean distance of baseline bursts to the baseline centroid is indicated by the *cyan line*. *Middle*: we captured Spearman's ρ between each seizure burst and its eight nearest temporal neighbors (see Figure 3a, b). This can be thought of as a measure of consistency of seizure bursts to each other in time. A three-second moving average of the ρ values is shown (*black line*). *Right*: for each burst, the R^2 value was determined from spatial linear regression (see *Methods*, section **Directionality of spike bursts and LFP discharges**). R^2 is a measure of the extent to which a burst is directional. A three-second moving average of the R^2 values is shown (*blue line*).

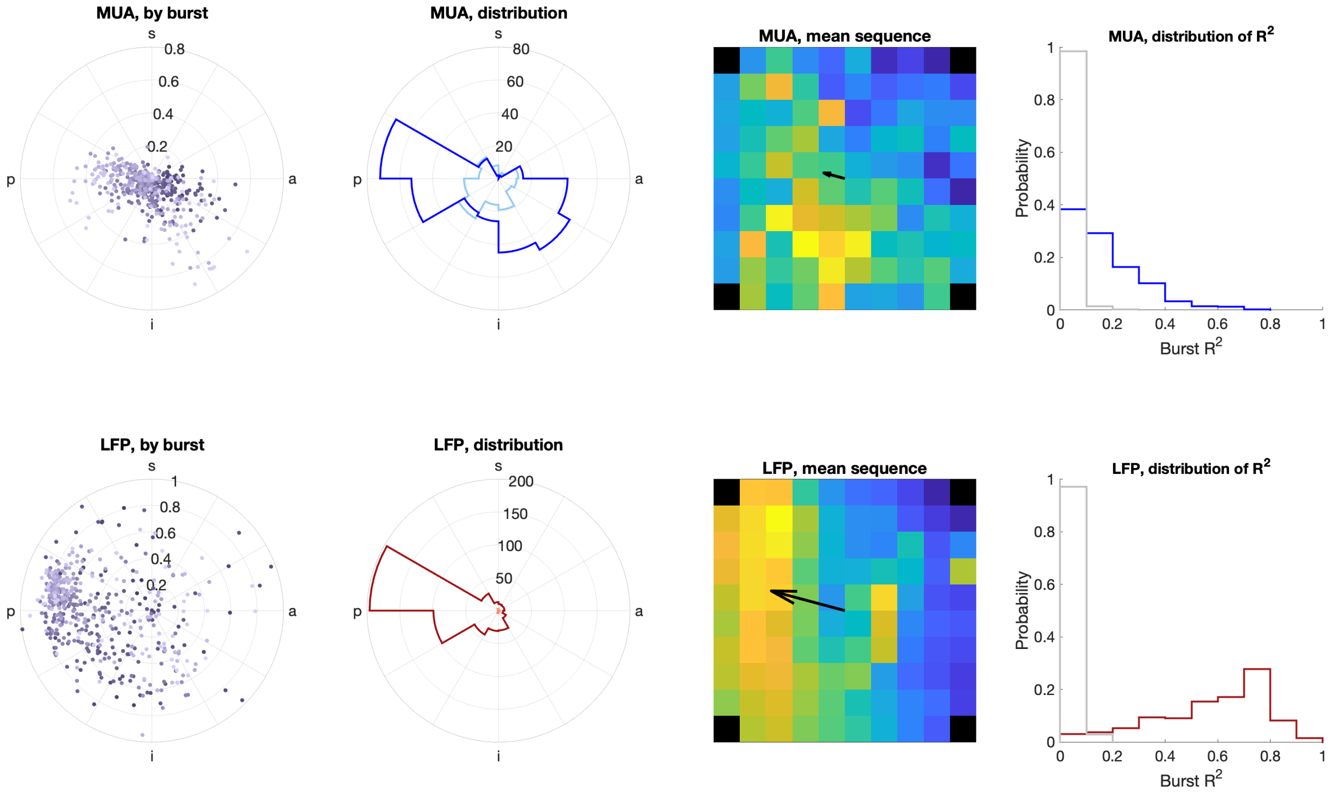

Supplementary Figure 103 Patient 3, array 1, seizure 1, seizure directionality. Top row: LFP. Bottom row: MUA. *Blue* histograms are used to designated MUA, while *maroon* histograms are used to designate LFP. First column from left: each dot represents a burst. θ reflects direction, and ρ is R^2, a measure of goodness of fit of the data to a plane. In other words, it is a measure of directionality of the burst. Second column from left: distribution direction of all recorded bursts. *Dark colors* indicate bursts with spatial linear regression giving $p \leq 0.05$, while *light* colors indicate bursts with $p > 0.05$. Third column from left: the average (*backbone*) sequence is shown over all bursts. This is obtained by taking the mean normalized rank of all sequences. The length of the *black arrow* indicates the mean R^2 over all bursts of this type, while the angle of the arrow indicates the mode of the histogram in the *second from left* column, for bursts with $p \leq 0.05$. Fourth column from left: R^2 for all bursts is shown. *Colored* histogram indicates the true sequences. *Gray* histogram, on the other hand, indicates R^2 for a set of null sequences, created by shuffling the ranks of the true sequences.

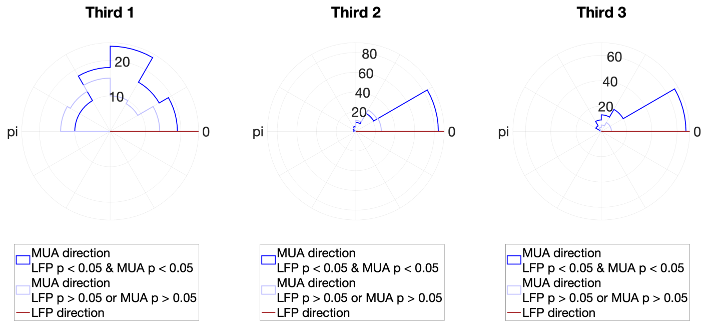

Supplementary Figure 104 Patient 3, array 1, seizure 1, relationship between MUA and LFP direction. Seizure bursts were divided into thirds (*left*: first third; *middle*: middle third, *right*: last third). For each third, we provided the distribution of burst-wise LFP-to-MUA directions. For each burst, LFP and MUA direction are compared, and the absolute value of the angular difference is put into the histogram. Bursts are divided into those with LFP and MUA p-value both less than 0.05 (*dark blue* histogram), and those with either MUA or LFP with p-value greater than 0.05. (*light blue* histogram).

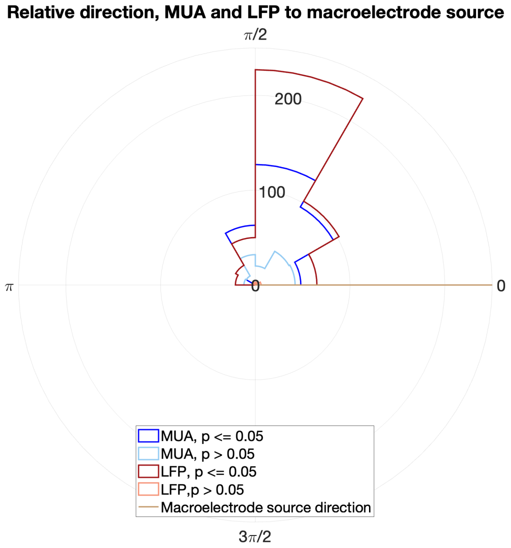

Supplementary Figure 105 Patient 3, array 1, seizure 1, relationship between discharge source, as determined by macroelectrode recordings, and direction of burst MUA and LFP. We used the time differences of the signal recorded in macroelectrodes to determine the location of the discharge source (Diamond, et al 2021, Diamond, et al 2023, see Supplementary Figure 12). The shortest path from the source to the array was then obtained, and we measured the angle of approach of the shortest path to the array (*tan* line). We then determined the relative direction of MUA and LFP signal, for each burst, to the discharge source as measured by macroelectrodes. Histograms are then provided, for the absolute value of the relative direction between source and MUA sequences (*blue*) and source and LFP sequences (*red*), for significant sequences (*dark* colors) and non-significant sequences (*light* colors).

Patient 4, array 1

Baseline activity

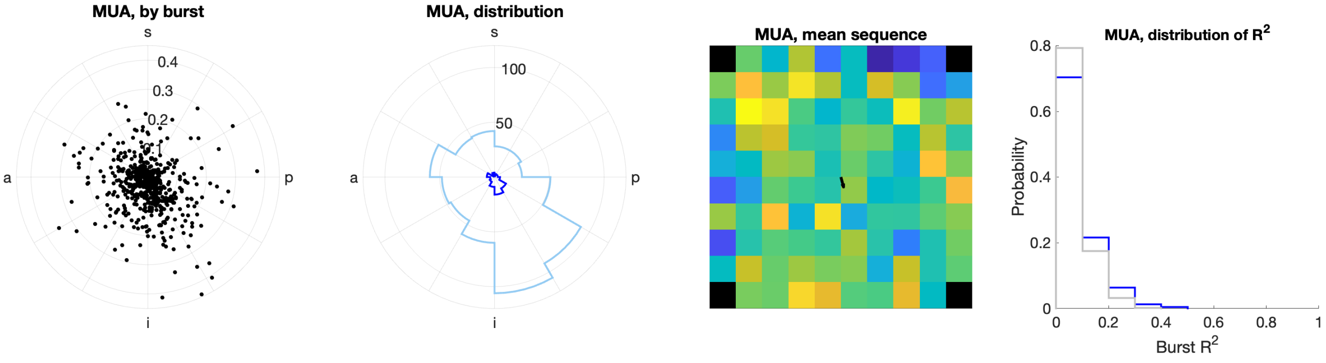

Supplementary Figure 106 Patient 4, array 1, baseline wavelike properties. First panel from left: each dot represents a burst. θ reflects direction, and ρ is R^2, a measure of goodness of fit of the data to a plane. In other words, it is a measure of the 'wavelike-ness' of the burst. Second panel from left: distribution direction of all recorded bursts. *Dark* colors indicate bursts with spatial linear regression giving $p \leq 0.05$, while *light* colors indicate bursts with $p > 0.05$. Third panel from left: the average (*backbone*) sequence is shown over all bursts. This is obtained by taking the mean normalized rank of all sequences. The length of the *black arrow* indicates the mean R^2 over all bursts of this type, while the angle of the arrow indicates the mode of the histogram in the *second from left* panel, for bursts with $p \leq 0.05$. Fourth panel from left: R^2 for all bursts is shown. *Blue* histogram indicates the true sequences. *Gray* histogram, on the other hand, indicates R^2 for a set of null sequences, created by shuffling the ranks of the true sequences.

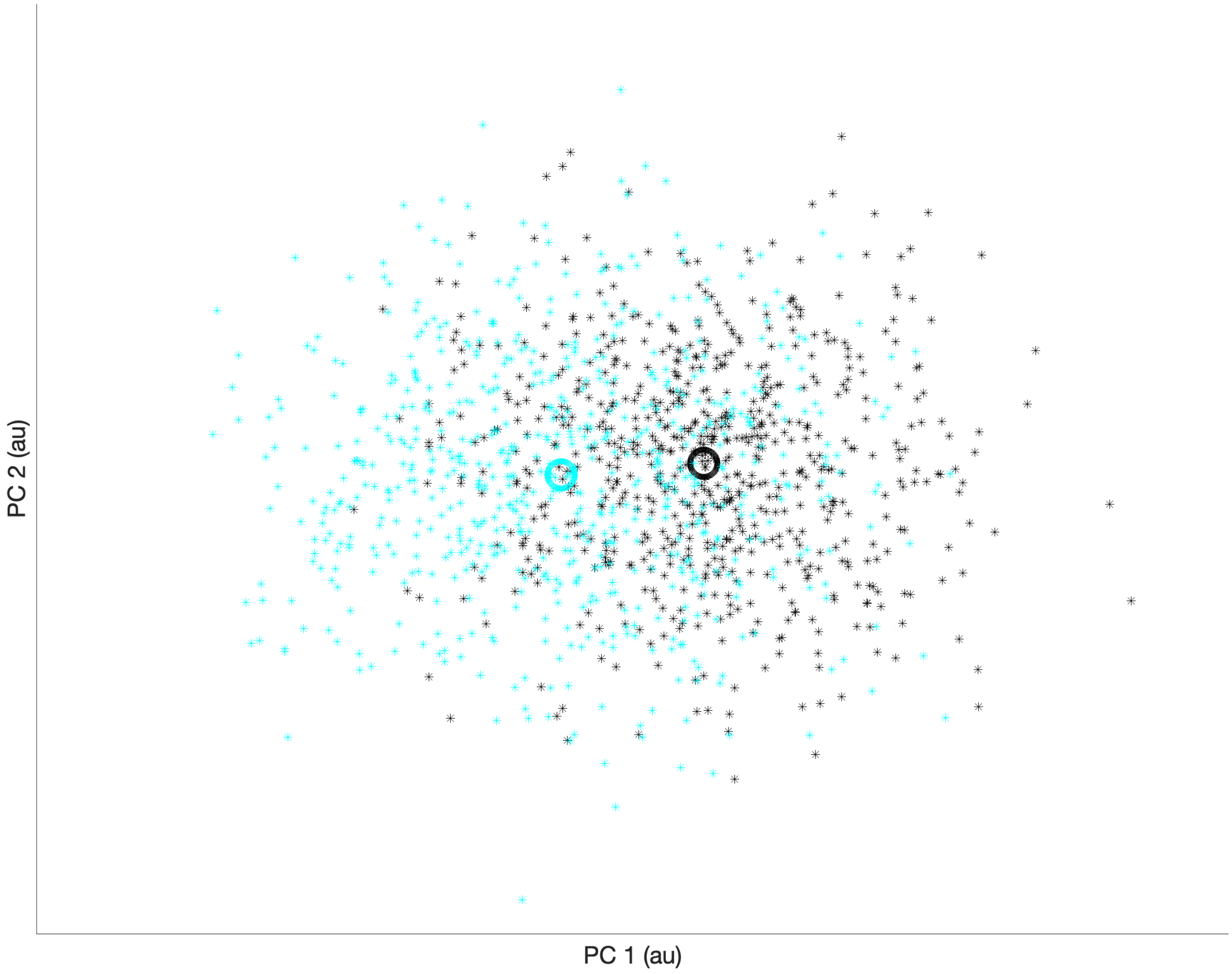

Supplementary Figure 107 Patient 4, array 1, baseline bursts versus random bursts. We were interested in determining that our baseline bursts were non-random. Therefore, for each baseline burst, we created a null counterpart, in which the MUA timings were shuffled only among spiking electrodes. We then applied both the true baselines and the random counterparts to the same dimensionality reduction procedure. The baseline centroid is indicated by the *blue circle*. The random centroid indicated by the *black* circle. For most patients and arrays, baseline bursts are displaced from the random bursts. Distance from baseline bursts to the random centroid tends to be greater than distance from random bursts to the random centroid (Supplementary Figure 4).

IED activity

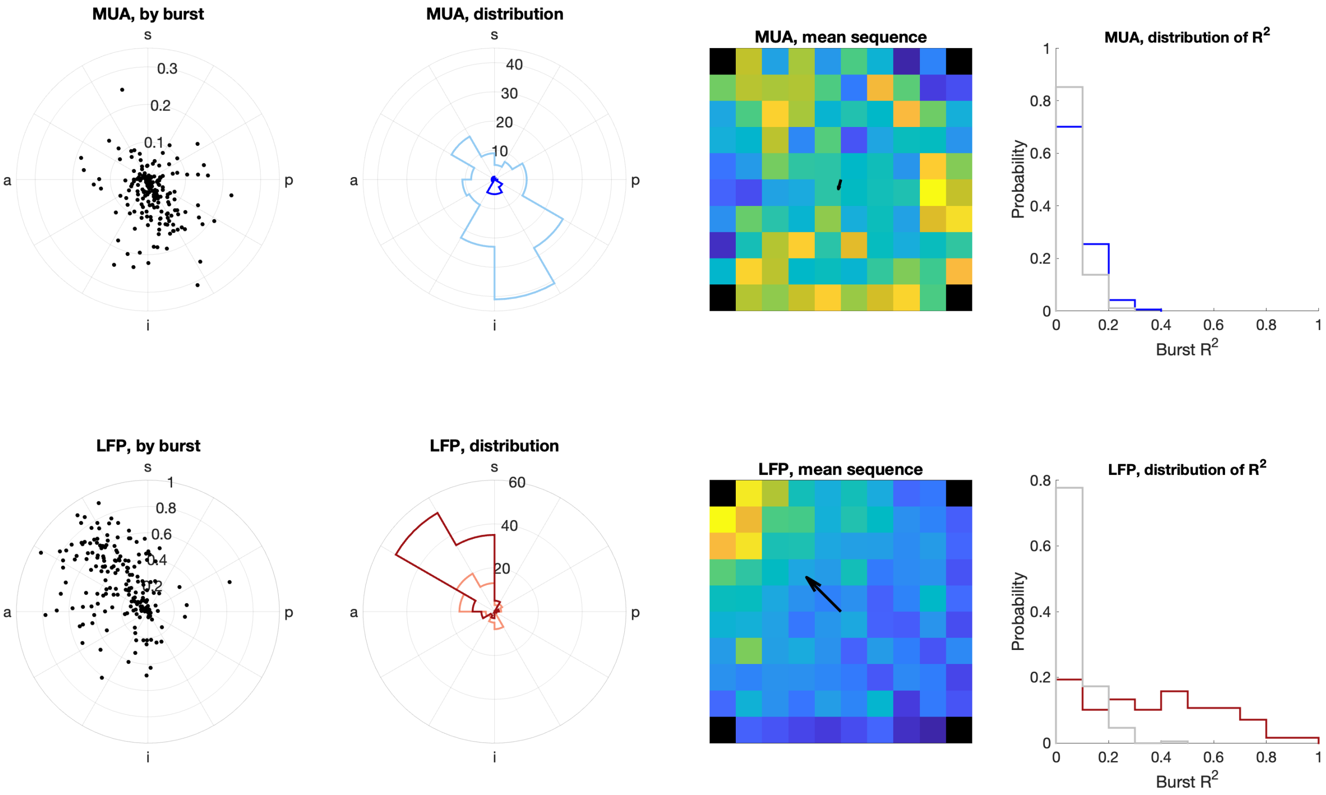

Supplementary Figure 108 Patient 4, array 1, IED directionality. Top row: LFP. Bottom row: MUA. *Dark blue* histograms are used to designated MUA, while *maroon* histograms are used to designate LFP. First column from left: each dot represents a burst. θ reflects direction, and ρ is R^2, a measure of goodness of fit of the data to a plane. In other words, it is a measure of the strength of directionality of the burst. Second column from left: distribution direction of all recorded bursts. *Dark colors* indicate bursts with spatial linear regression giving $p \leq 0.05$, while *light* colors indicate bursts with $p > 0.05$. Third column from left: the average (*backbone*) sequence is shown over all bursts for this seizure. This is obtained by taking the mean normalized rank of all sequences. The length of the *black arrow* indicates the mean R^2 over all bursts of this type, while the angle of the arrow indicates the mode of the histogram in the *second from left* column, for bursts with $p \leq 0.05$. Fourth column from left: R^2 for all bursts is shown. *Colored* histogram indicates the true sequences. *Gray* histogram, on the other hand, indicates R^2 for a set of null sequences, created by shuffling the ranks of spiking electrodes in the true sequences.

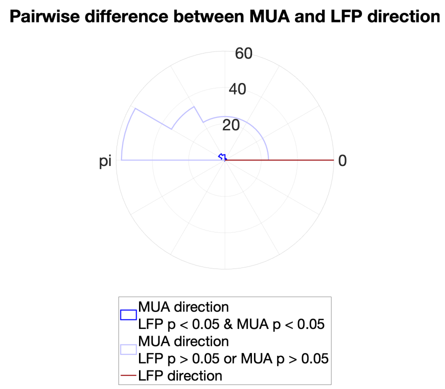

Supplementary Figure 109 Patient 4, array 1, IED relationship between MUA and LFP direction. Distribution of pairwise LFP-to-MUA direction. For each burst, LFP and MUA direction are compared, and the absolute value of the angular difference is put into the histogram. Bursts are divided into those with LFP and MUA p-value both less than 0.05, or those with either MUA or LFP with p-value greater than 0.05.

Seizure activity, seizure 1

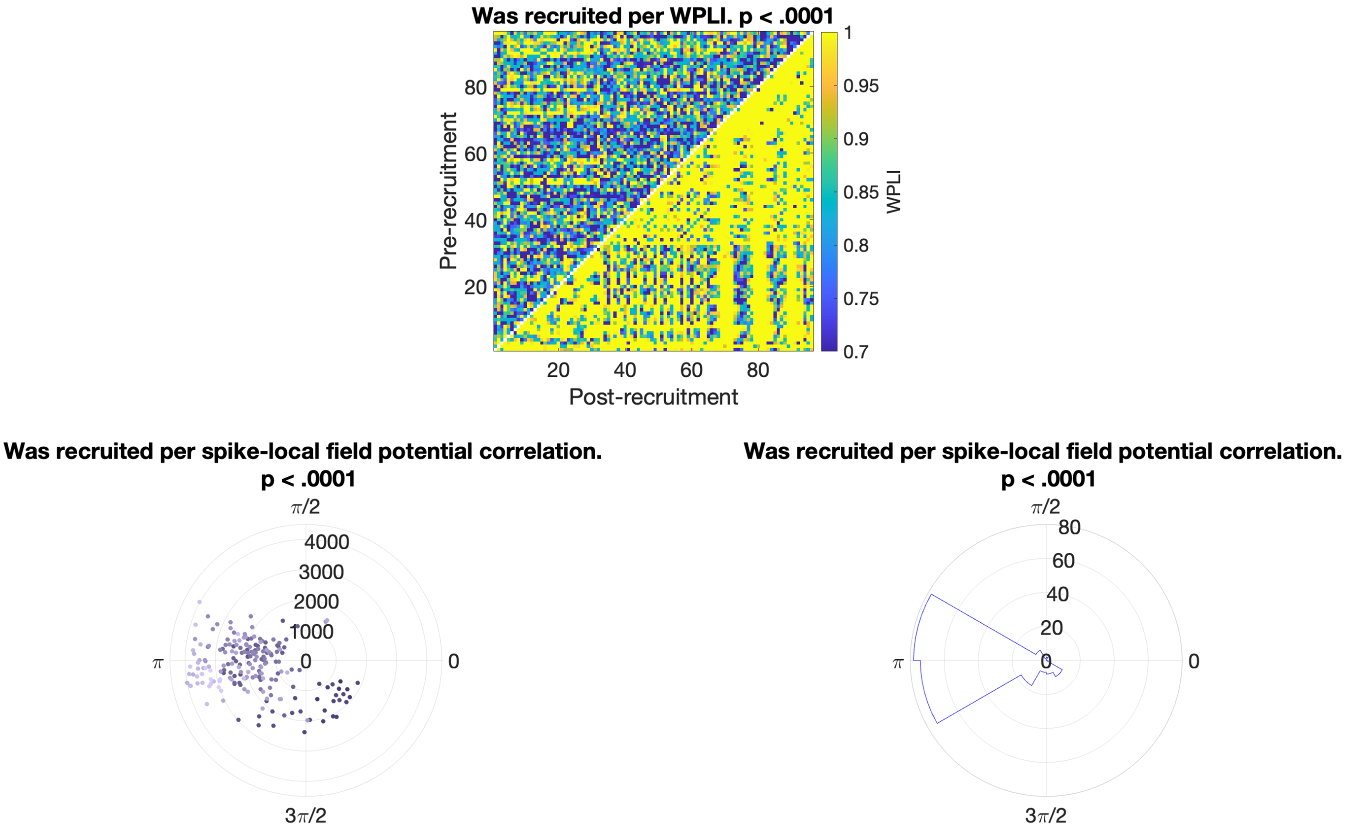

Supplementary Figure 110 Measures of recruitment for patient 4, array 1, seizure 1. Top: weighted phase lag index (WPLI), 10 seconds prior to seizure onset (top left), and 10 seconds after recruitment (bottom right, see *Methods*). Bottom left: relationship between LFP phase (θ) and LFP phase power (ρ), over the course of the seizure. *Dark purple* dots: early seizure; *light purple* dots: late seizure. Bottom right: distribution of spike-local field correlation values.

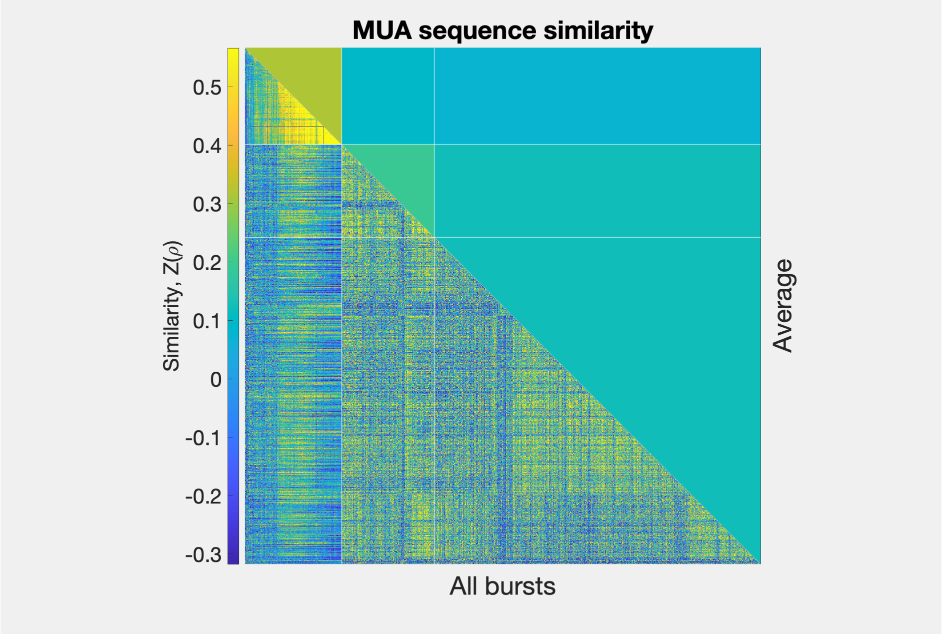

Supplementary Figure 111 Patient 4, array 1, seizure 1, burst similarity. For this patient, array, and seizure, every sequence was compared to every other sequence. Each cell indicates the similarity of the row sequence to the column sequence (Spearman's ρ). Sequences are divided by *white* lines into seizure sequences (*left, top*), IED sequences (*middle*), and baseline sequences (*right, bottom*). Actual values are provided in the lower triangle. In the upper triangle, average values are provided for each group, with values represented by the same color axis.

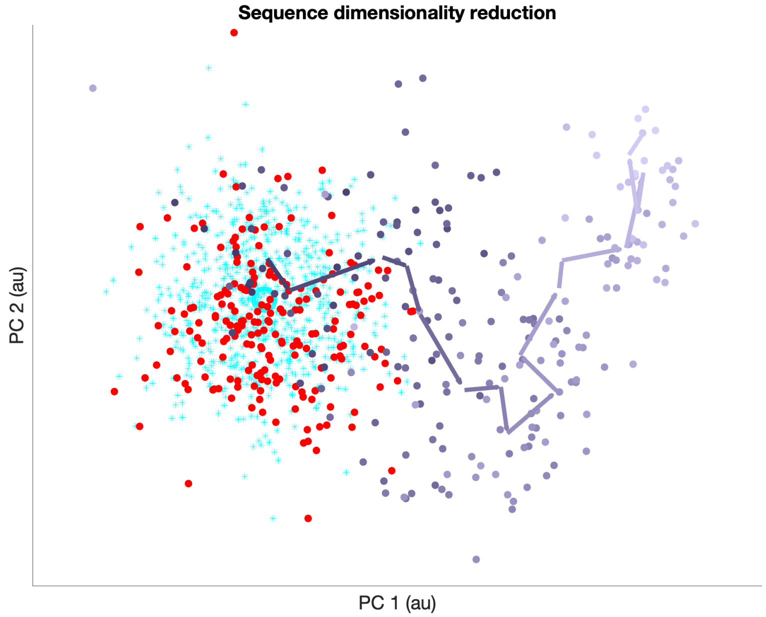

Supplementary Figure 112 Patient 4, array 1, seizure 1, dimensionality reduction using principal component analysis (PCA) was performed. We used PCA to decompose high-dimensional data into two dimensions in an unsupervised fashion (see *Methods*). Data provided include baseline bursts (*cyan stars*), IED bursts (*red dots*), and seizure bursts (*colored dots*; *dark purple*, early seizure, *light purple*, late seizure). The baseline centroid is indicated by the *cyan circle*.

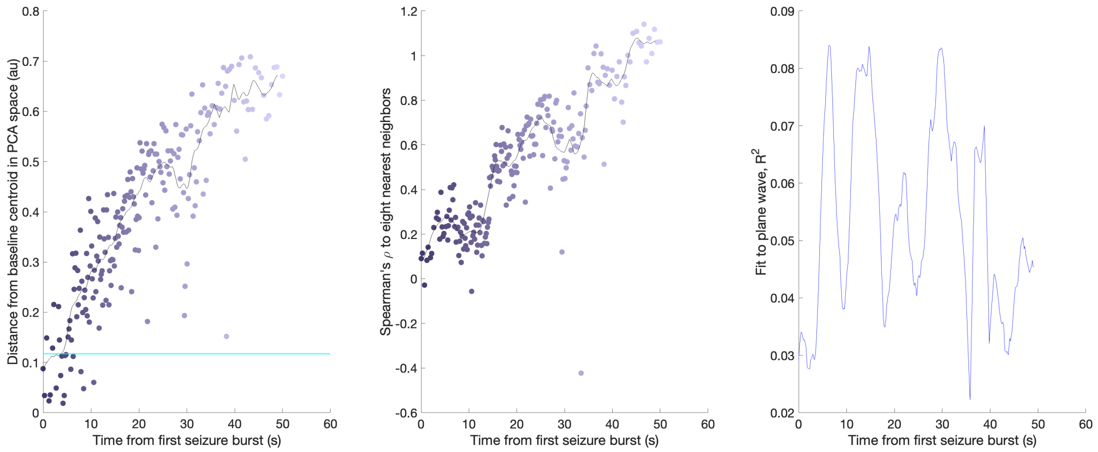

Supplementary Figure 113 Patient 4, array 1, seizure 1, measures of wave entrainment over time. *Left*: We used dimensionality reduction to collapse high-dimensional sequence information into two dimensions (see Figure 2a). We then took the location of the baseline centroid, and captured the distance, over time, between seizure bursts and the baseline centroid, in the low-dimensional manifold (see Figure 2c). *Dark purple* dots: early seizure; *light purple* dots: late seizure. A three-second moving average is superimposed (*black line*). Mean distance of baseline bursts to the baseline centroid is indicated by the *cyan line*. *Middle*: we captured Spearman's ρ between each seizure burst and its eight nearest temporal neighbors (see Figure 3a, b). This can be thought of as a measure of consistency of seizure bursts to each other in time. A three-second moving average of the ρ values is shown (*black line*). *Right*: for each burst, the R^2 value was determined from spatial linear regression (see *Methods*, section **Directionality of spike bursts and LFP discharges**). R^2 is a measure of the extent to which a burst is directional. A three-second moving average of the R^2 values is shown (*blue line*).

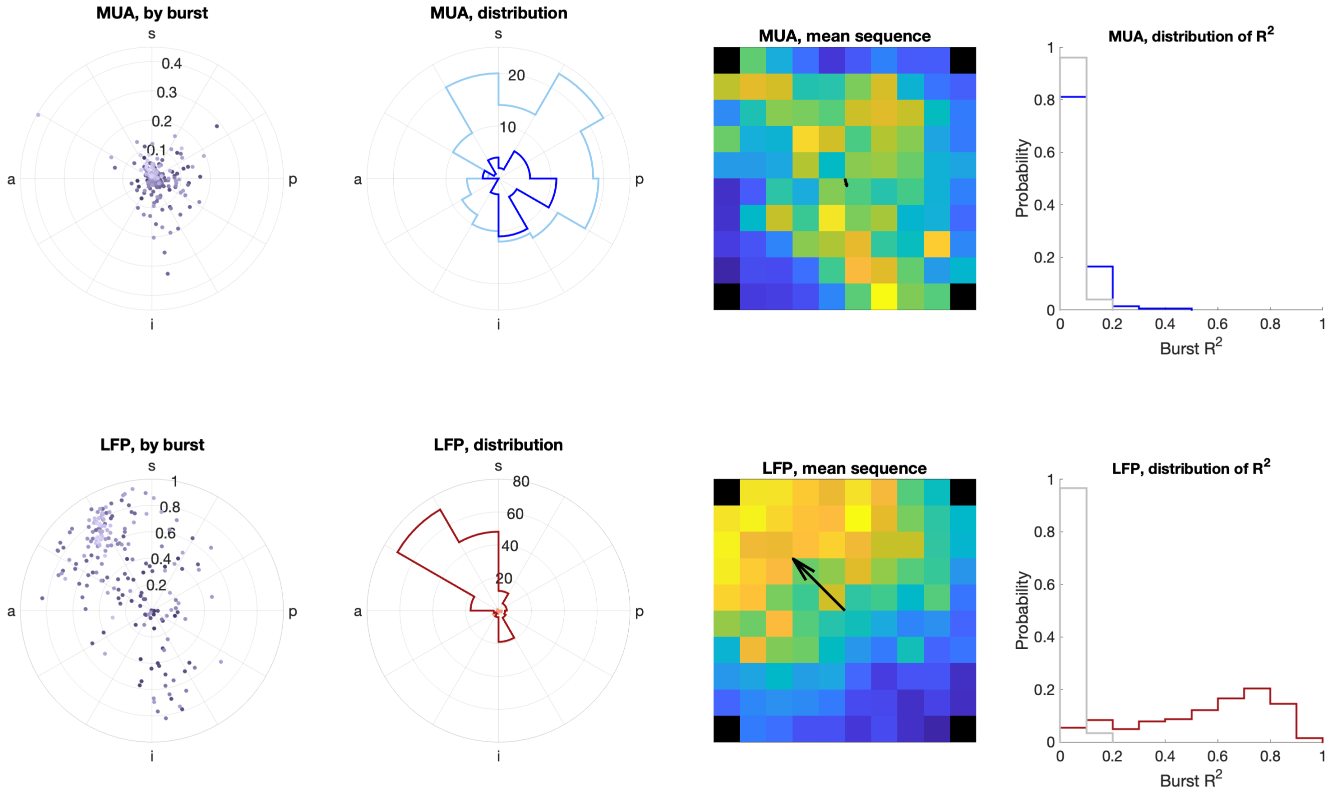

Supplementary Figure 114 Patient 4, array 1, seizure 1, seizure directionality. Top row: LFP. Bottom row: MUA. *Blue* histograms are used to designated MUA, while *maroon* histograms are used to designate LFP. First column from left: each dot represents a burst. θ reflects direction, and ρ is R^2, a measure of goodness of fit of the data to a plane. In other words, it is a measure of directionality of the burst. Second column from left: distribution direction of all recorded bursts. *Dark colors* indicate bursts with spatial linear regression giving $p \leq 0.05$, while *light* colors indicate bursts with $p > 0.05$. Third column from left: the average (*backbone*) sequence is shown over all bursts. This is obtained by taking the mean normalized rank of all sequences. The length of the *black arrow* indicates the mean R^2 over all bursts of this type, while the angle of the arrow indicates the mode of the histogram in the *second from left* column, for bursts with $p \leq 0.05$. Fourth column from left: R^2 for all bursts is shown. *Colored* histogram indicates the true sequences. *Gray* histogram, on the other hand, indicates R^2 for a set of null sequences, created by shuffling the ranks of the true sequences.

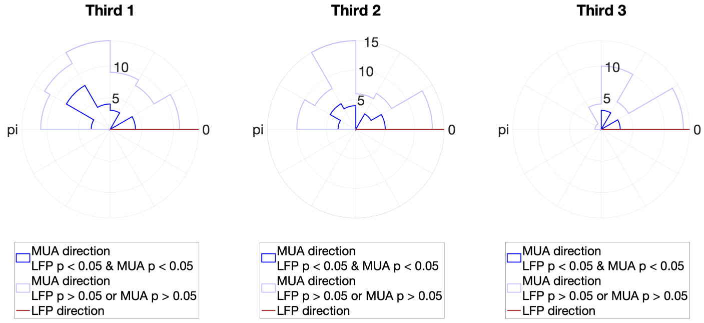

Supplementary Figure 115 Patient 4, array 1, seizure 1, relationship between MUA and LFP direction. Seizure bursts were divided into thirds (*left*: first third; *middle*: middle third, *right*: last third). For each third, we provided the distribution of burst-wise LFP-to-MUA directions. For each burst, LFP and MUA direction are compared, and the absolute value of the angular difference is put into the histogram. Bursts are divided into those with LFP and MUA p-value both less than 0.05 (*dark blue* histogram), and those with either MUA or LFP with p-value greater than 0.05. (*light blue* histogram).

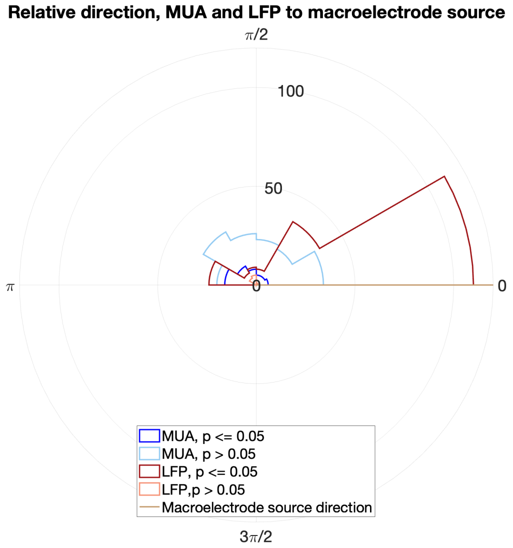

Supplementary Figure 116 Patient 4, array 1, seizure 1, relationship between discharge source, as determined by macroelectrode recordings, and direction of burst MUA and LFP. We used the time differences of the signal recorded in macroelectrodes to determine the location of the discharge source (Diamond, et al 2021, Diamond, et al 2023, see Supplementary Figure 12). The shortest path from the source to the array was then obtained, and we measured the angle of approach of the shortest path to the array (*tan* line). We then determined the relative direction of MUA and LFP signal, for each burst, to the discharge source as measured by macroelectrodes. Histograms are then provided, for the absolute value of the relative direction between source and MUA sequences (*blue*) and source and LFP sequences (*red*), for significant sequences (*dark* colors) and non-significant sequences (*light* colors).

Seizure activity, seizure 2

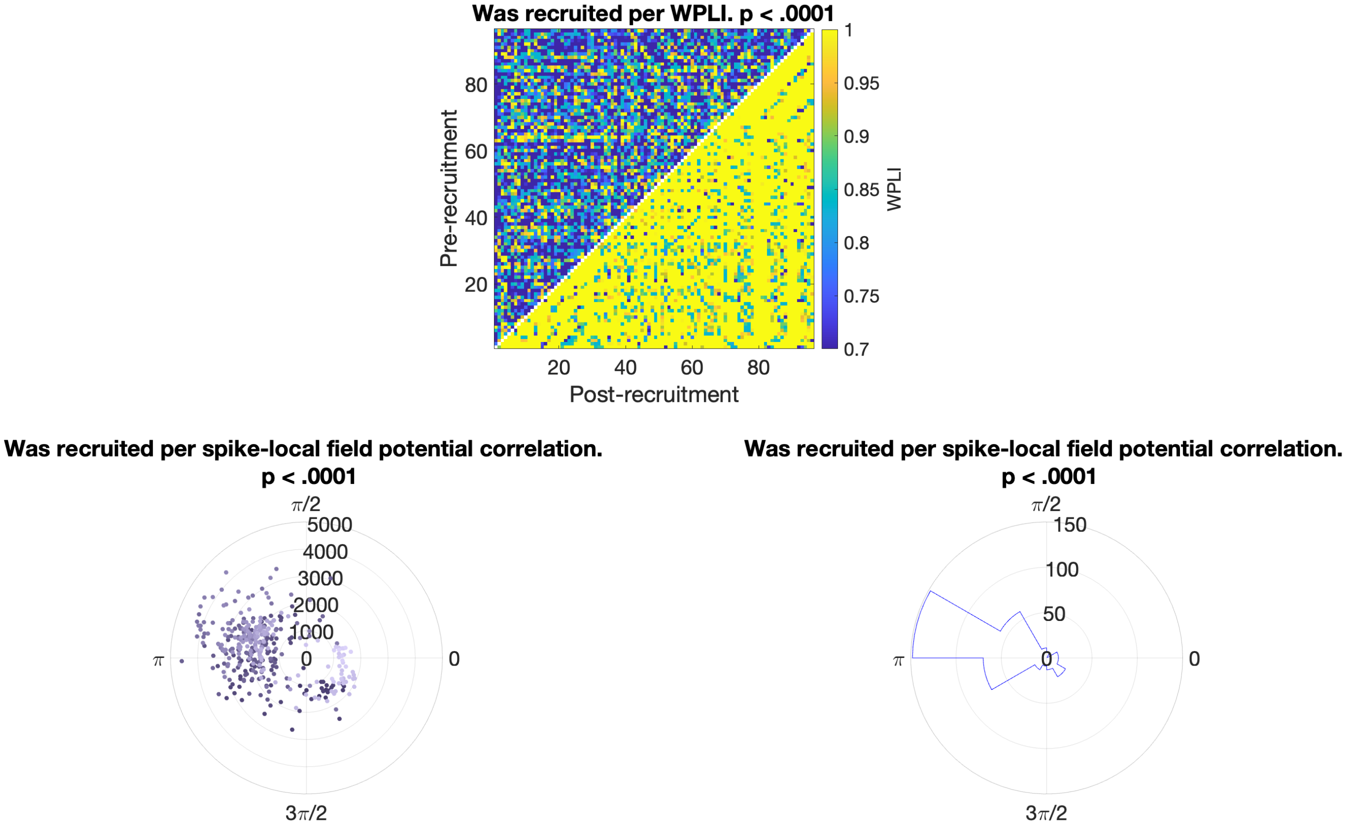

Supplementary Figure 117 Measures of recruitment for patient 4, array 1, seizure 2. Top: weighted phase lag index (WPLI), 10 seconds prior to seizure onset (top left), and 10 seconds after recruitment (bottom right, see *Methods*). Bottom left: relationship between LFP phase (θ) and LFP phase power (ρ), over the course of the seizure. *Dark purple* dots: early seizure; *light purple* dots: late seizure. Bottom right: distribution of spike-local field correlation values.

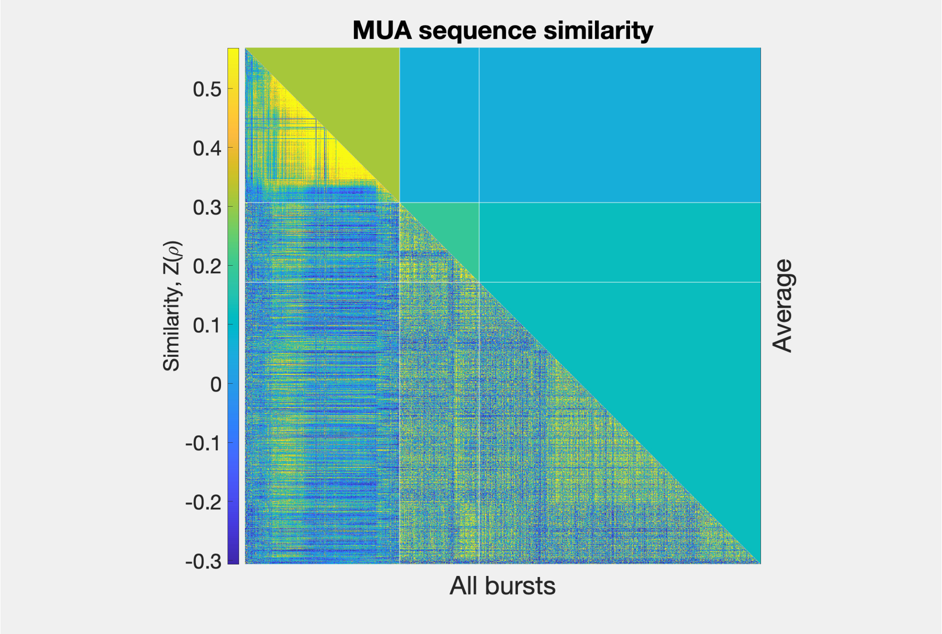

Supplementary Figure 118 Patient 4, array 1, seizure 2, burst similarity. For this patient, array, and seizure, every sequence was compared to every other sequence. Each cell indicates the similarity of the row sequence to the column sequence (Spearman's ρ). Sequences are divided by *white* lines into seizure sequences (*left, top*), IED sequences (*middle*), and baseline sequences (*right, bottom*). Actual values are provided in the lower triangle. In the upper triangle, average values are provided for each group, with values represented by the same color axis.

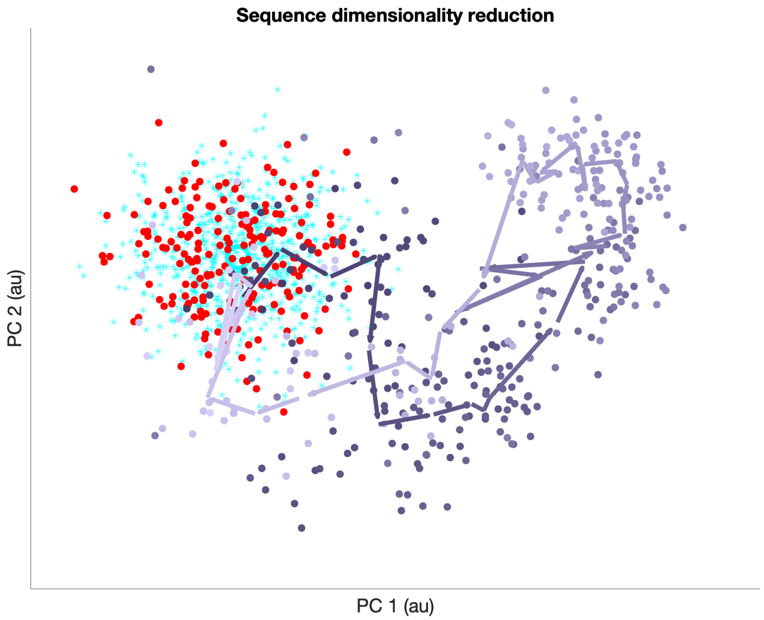

Supplementary Figure 119 Patient 4, array 1, seizure 2, dimensionality reduction using principal component analysis (PCA) was performed. We used PCA to decompose high-dimensional data into two dimensions in an unsupervised fashion (see *Methods*). Data provided include baseline bursts (*cyan stars*), IED bursts (*red dots*), and seizure bursts (*colored dots*; *dark purple*, early seizure, *light purple*, late seizure). The baseline centroid is indicated by the *cyan circle*.

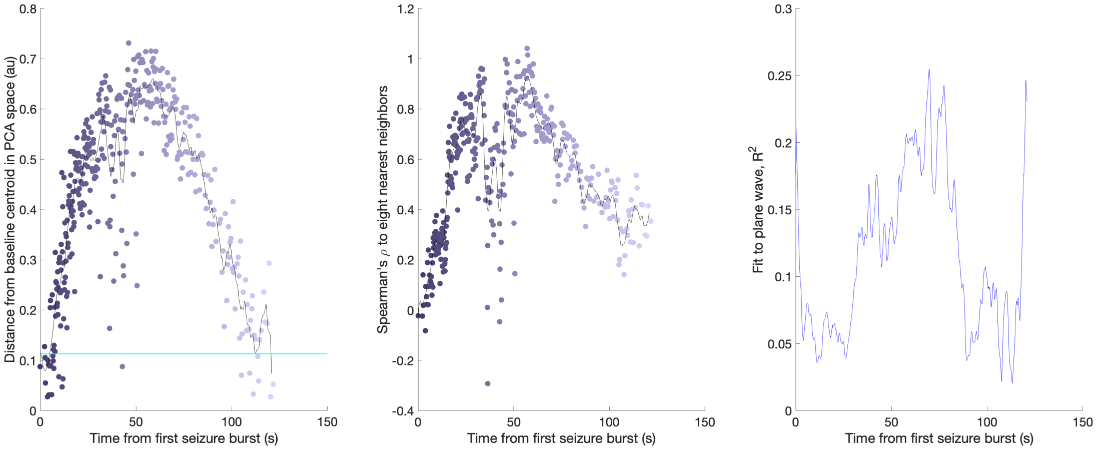

Supplementary Figure 120 Patient 4, array 1, seizure 2, measures of wave entrainment over time. *Left*: We used dimensionality reduction to collapse high-dimensional sequence information into two dimensions (see Figure 2a). We then took the location of the baseline centroid, and captured the distance, over time, between seizure bursts and the baseline centroid, in the low-dimensional manifold (see Figure 2c). *Dark purple* dots: early seizure; *light purple* dots: late seizure. A three-second moving average is superimposed (*black line*). Mean distance of baseline bursts to the baseline centroid is indicated by the *cyan line*. *Middle*: we captured Spearman's ρ between each seizure burst and its eight nearest temporal neighbors (see Figure 3a, b). This can be thought of as a measure of consistency of seizure bursts to each other in time. A three-second moving average of the ρ values is shown (*black line*). *Right*: for each burst, the R^2 value was determined from spatial linear regression (see *Methods*, section **Directionality of spike bursts and LFP discharges**). R^2 is a measure of the extent to which a burst is directional. A three-second moving average of the R^2 values is shown (*blue line*).

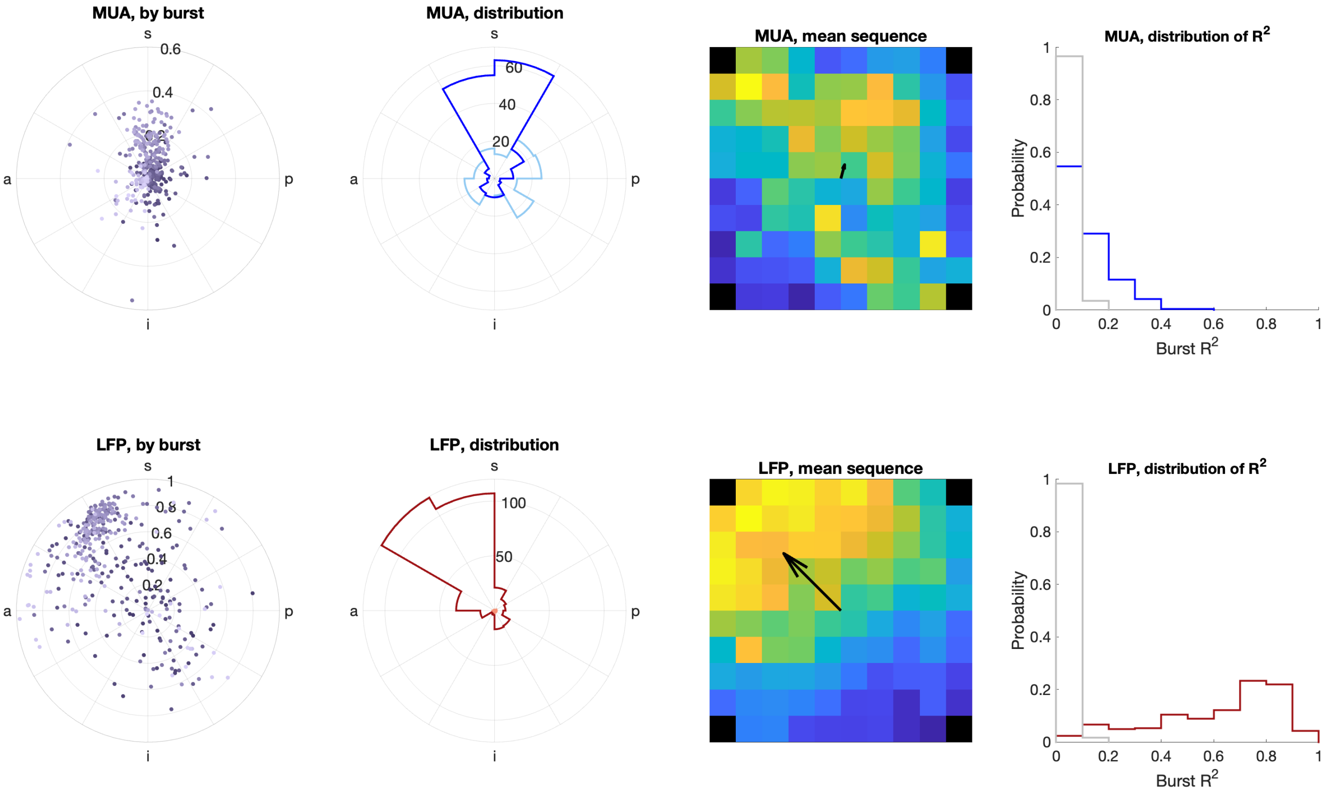

Supplementary Figure 121 Patient 4, array 1, seizure 2, seizure directionality. Top row: LFP. Bottom row: MUA. *Blue* histograms are used to designated MUA, while *maroon* histograms are used to designate LFP. First column from left: each dot represents a burst. θ reflects direction, and ρ is R^2, a measure of goodness of fit of the data to a plane. In other words, it is a measure of directionality of the burst. Second column from left: distribution direction of all recorded bursts. *Dark colors* indicate bursts with spatial linear regression giving $p \leq 0.05$, while *light* colors indicate bursts with $p > 0.05$. Third column from left: the average (*backbone*) sequence is shown over all bursts. This is obtained by taking the mean normalized rank of all sequences. The length of the *black arrow* indicates the mean R^2 over all bursts of this type, while the angle of the arrow indicates the mode of the histogram in the *second from left* column, for bursts with $p \leq 0.05$. Fourth column from left: R^2 for all bursts is shown. *Colored* histogram indicates the true sequences. *Gray* histogram, on the other hand, indicates R^2 for a set of null sequences, created by shuffling the ranks of the true sequences.

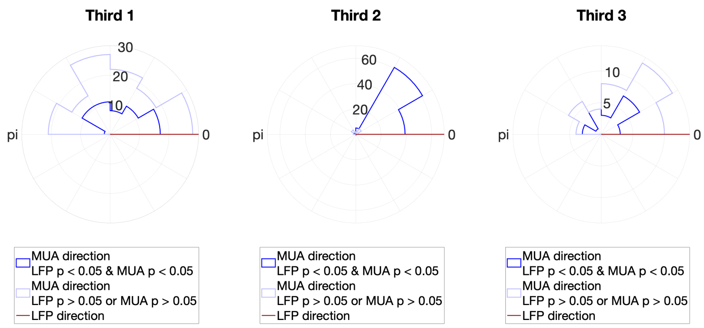

Supplementary Figure 122 Patient 4, array 1, seizure 2, relationship between MUA and LFP direction. Seizure bursts were divided into thirds (*left*: first third; *middle*: middle third, *right*: last third). For each third, we provided the distribution of burst-wise LFP-to-MUA directions. For each burst, LFP and MUA direction are compared, and the absolute value of the angular difference is put into the histogram. Bursts are divided into those with LFP and MUA p-value both less than 0.05 (*dark blue* histogram), and those with either MUA or LFP with p-value greater than 0.05. (*light blue* histogram).

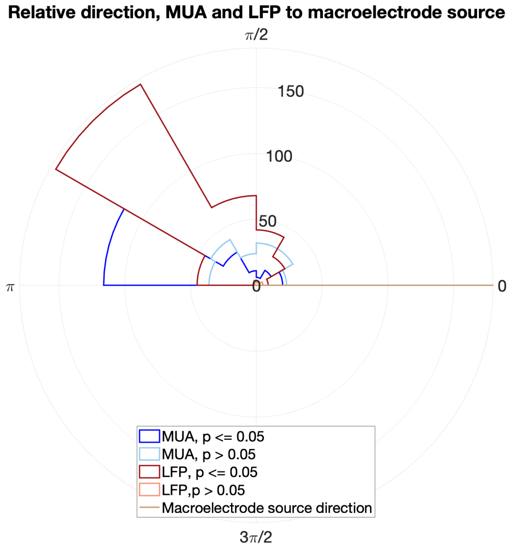

Supplementary Figure 123 Patient 4, array 1, seizure 2, relationship between discharge source, as determined by macroelectrode recordings, and direction of burst MUA and LFP. We used the time differences of the signal recorded in macroelectrodes to determine the location of the discharge source (Diamond, et al 2021, Diamond, et al 2023, see Supplementary Figure 12). The shortest path from the source to the array was then obtained, and we measured the angle of approach of the shortest path to the array (*tan* line). We then determined the relative direction of MUA and LFP signal, for each burst, to the discharge source as measured by macroelectrodes. Histograms are then provided, for the absolute value of the relative direction between source and MUA sequences (*blue*) and source and LFP sequences (*red*), for significant sequences (*dark* colors) and non-significant sequences (*light* colors).

Patient 5, array 1

Baseline activity

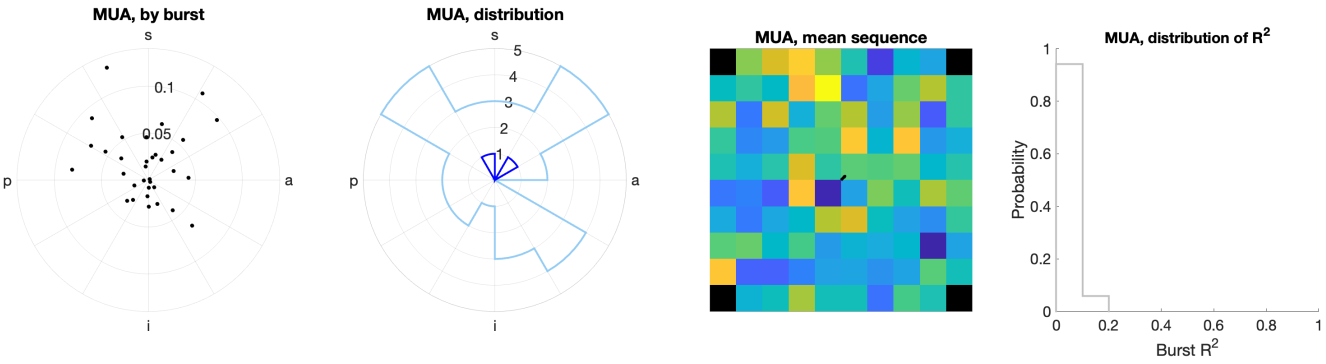

Supplementary Figure 124 Patient 5, array 1, baseline wavelike properties. First panel from left: each dot represents a burst. θ reflects direction, and ρ is R^2, a measure of goodness of fit of the data to a plane. In other words, it is a measure of the 'wavelike-ness' of the burst. Second panel from left: distribution direction of all recorded bursts. *Dark* colors indicate bursts with spatial linear regression giving $p \leq 0.05$, while *light* colors indicate bursts with $p > 0.05$. Third panel from left: the average (*backbone*) sequence is shown over all bursts. This is obtained by taking the mean normalized rank of all sequences. The length of the *black arrow* indicates the mean R^2 over all bursts of this type, while the angle of the arrow indicates the mode of the histogram in the *second from left* panel, for bursts with $p \leq 0.05$. Fourth panel from left: R^2 for all bursts is shown. *Blue* histogram indicates the true sequences. *Gray* histogram, on the other hand, indicates R^2 for a set of null sequences, created by shuffling the ranks of the true sequences.

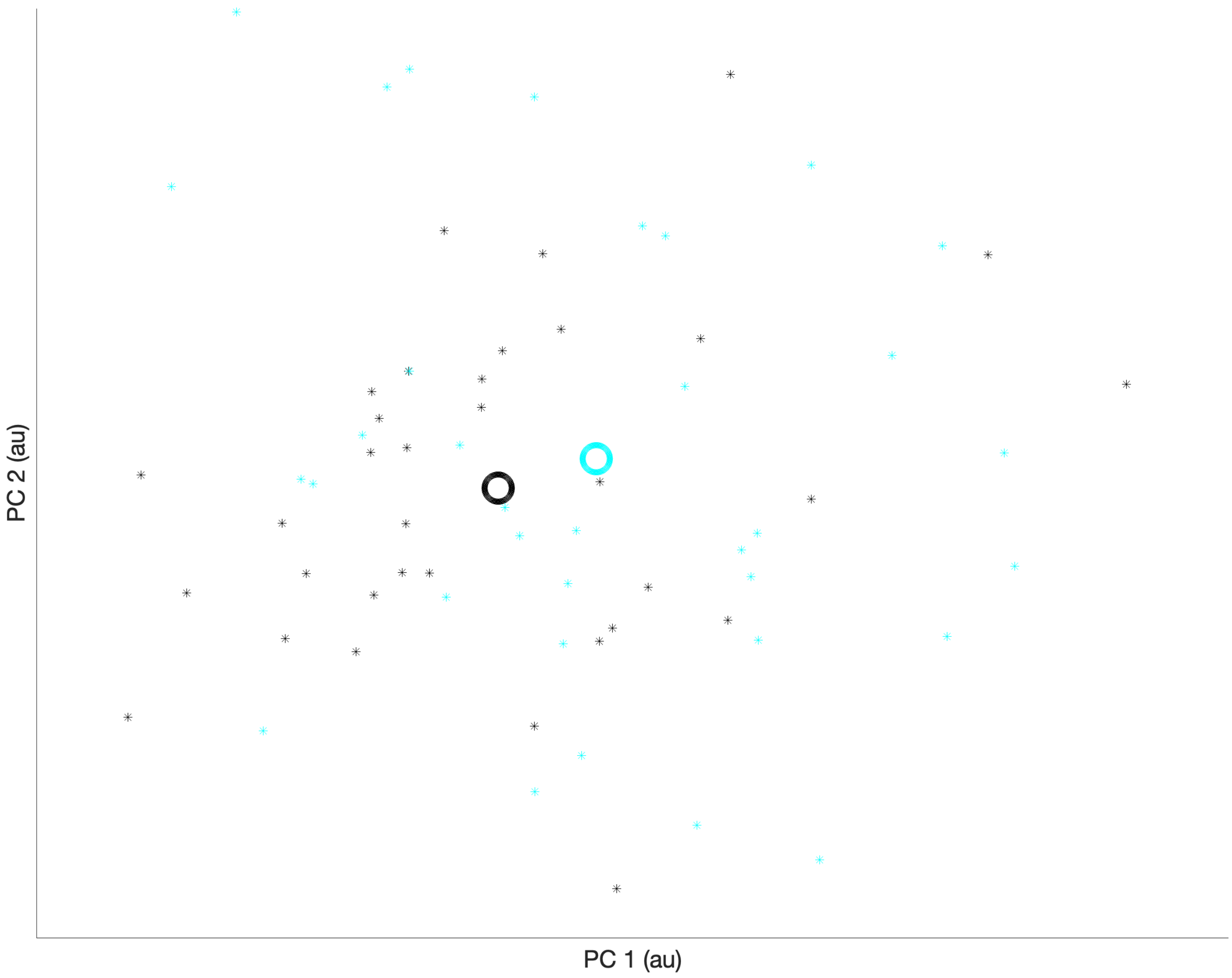

Supplementary Figure 125 Patient 5, array 1, baseline bursts versus random bursts. We were interested in determining that our baseline bursts were non-random. Therefore, for each baseline burst, we created a null counterpart, in which the MUA timings were shuffled only among spiking electrodes. We then applied both the true baselines and the random counterparts to the same dimensionality reduction procedure. The baseline centroid is indicated by the *blue circle*. The random centroid indicated by the *black* circle. For most patients and arrays, baseline bursts are displaced from the random bursts. Distance from baseline bursts to the random centroid tends to be greater than distance from random bursts to the random centroid (Supplementary Figure 4).

IED activity

Supplementary Figure 126 Patient 5, array 1, IED directionality. No results are available for this analysis. No IEDs were recorded on the MEA.

Supplementary Figure 127 Patient 5, array 1, IED relationship between MUA and LFP direction. No results are available for this analysis. No IEDs were recorded on the MEA.

Seizure activity, seizure 5

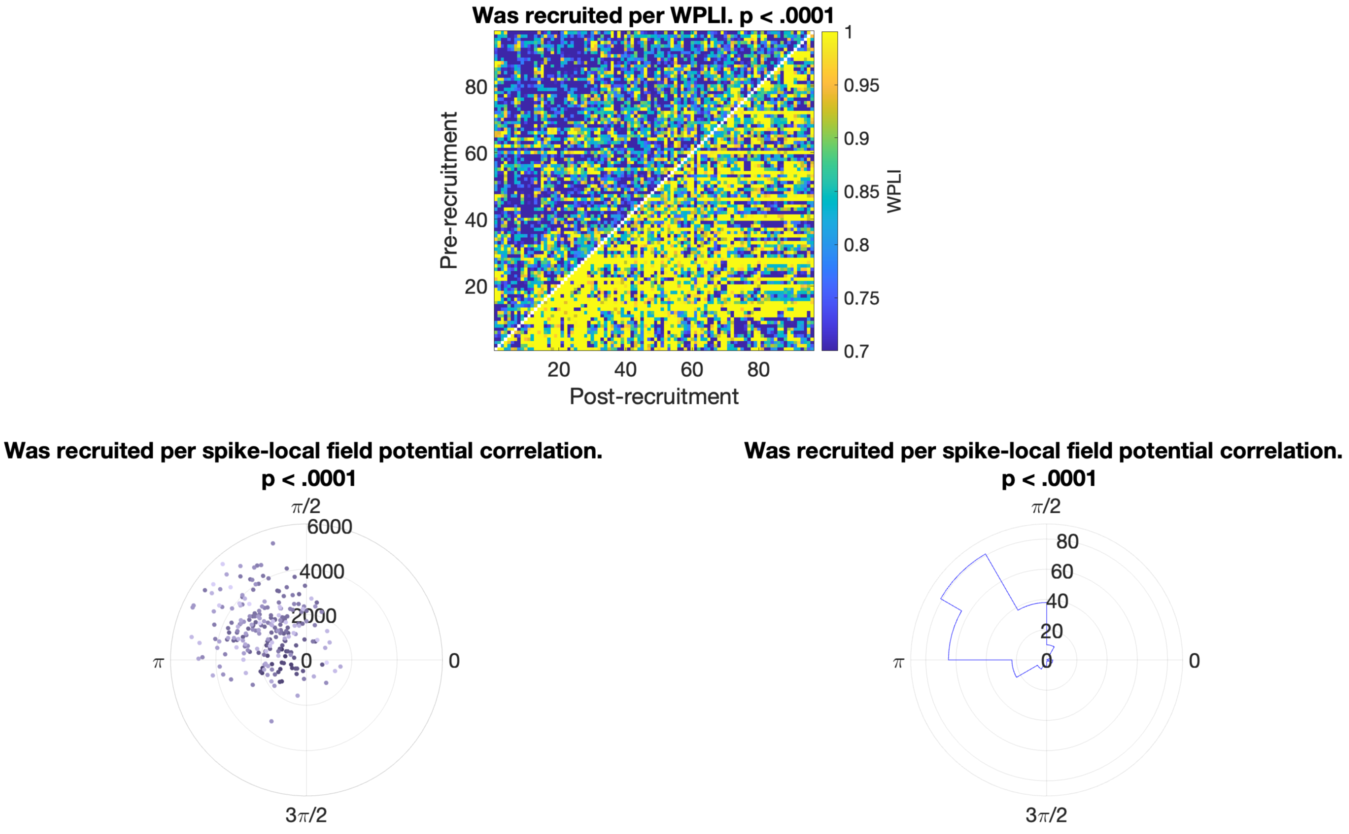

Supplementary Figure 128 Measures of recruitment for patient 5, array 1, seizure 5. Top: weighted phase lag index (WPLI), 10 seconds prior to seizure onset (top left), and 10 seconds after recruitment (bottom right, see *Methods*). Bottom left: relationship between LFP phase (θ) and LFP phase power (ρ), over the course of the seizure. *Dark purple* dots: early seizure; *light purple* dots: late seizure. Bottom right: distribution of spike-local field correlation values.

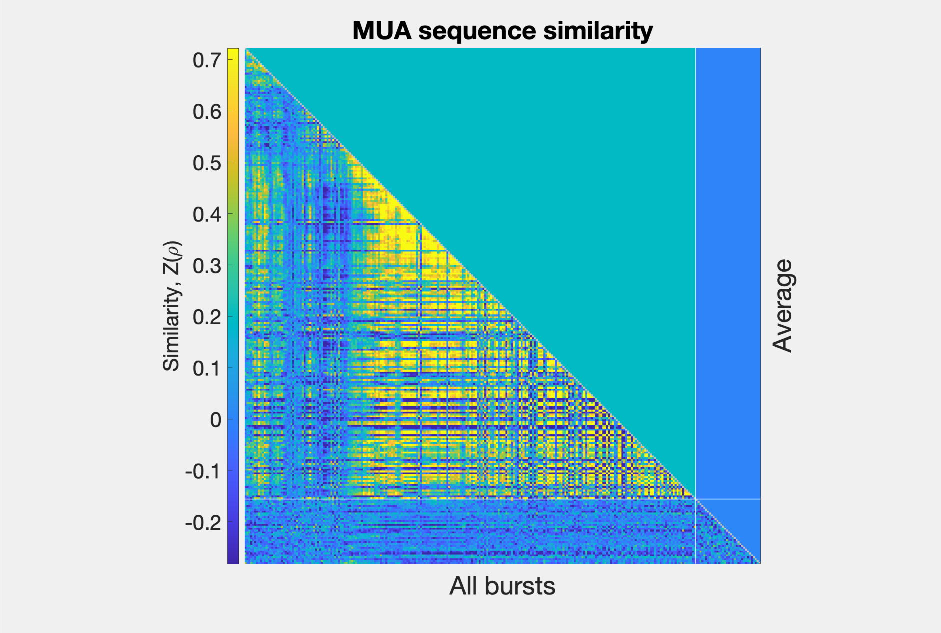

Supplementary Figure 129 Patient 5, array 1, seizure 5, burst similarity. For this patient, array, and seizure, every sequence was compared to every other sequence. Each cell indicates the similarity of the row sequence to the column sequence (Spearman's ρ). Sequences are divided by *white* lines into seizure sequences (*left, top*), IED sequences (*middle*), and baseline sequences (*right, bottom*). Actual values are provided in the lower triangle. In the upper triangle, average values are provided for each group, with values represented by the same color axis.

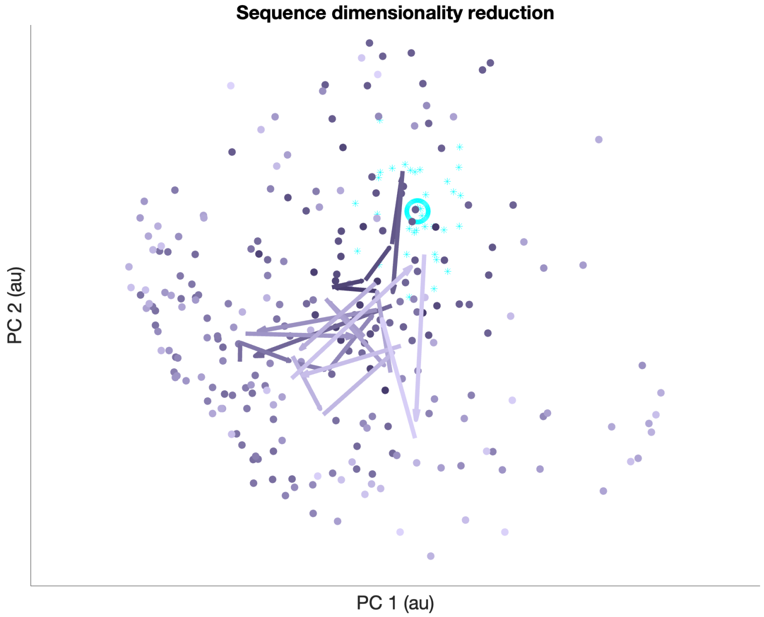

Supplementary Figure 130 Patient 5, array 1, seizure 5, dimensionality reduction using principal component analysis (PCA) was performed. We used PCA to decompose high-dimensional data into two dimensions in an unsupervised fashion (see *Methods*). Data provided include baseline bursts (*cyan stars*), IED bursts (*red dots*), and seizure bursts (*colored dots*; *dark purple*, early seizure, *light purple*, late seizure). The baseline centroid is indicated by the *cyan circle*.

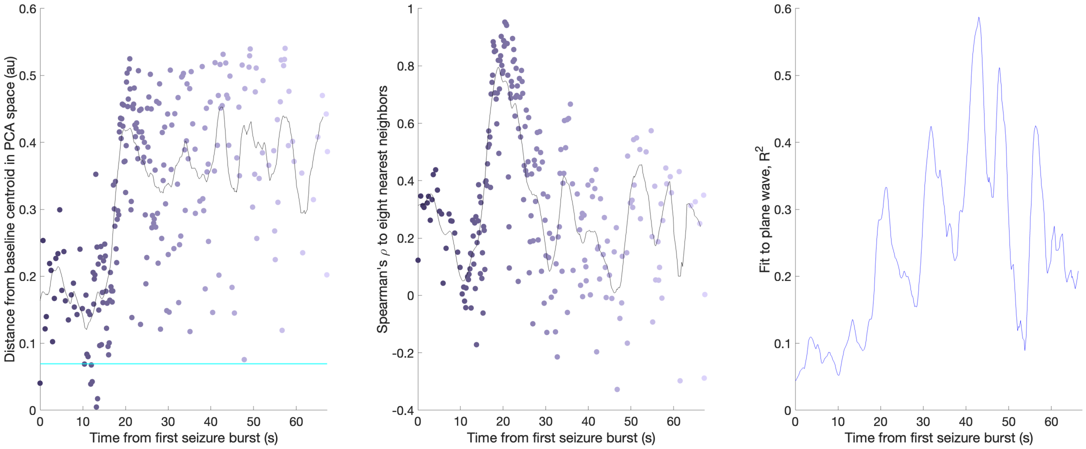

Supplementary Figure 131 Patient 5, array 1, seizure 5, measures of wave entrainment over time. *Left*: We used dimensionality reduction to collapse high-dimensional sequence information into two dimensions (see Figure 2a). We then took the location of the baseline centroid, and captured the distance, over time, between seizure bursts and the baseline centroid, in the low-dimensional manifold (see Figure 2c). *Dark purple* dots: early seizure; *light purple* dots: late seizure. A three-second moving average is superimposed (*black line*). Mean distance of baseline bursts to the baseline centroid is indicated by the *cyan line*. *Middle*: we captured Spearman's ρ between each seizure burst and its eight nearest temporal neighbors (see Figure 3a, b). This can be thought of as a measure of consistency of seizure bursts to each other in time. A three-second moving average of the ρ values is shown (*black line*). *Right*: for each burst, the R^2 value was determined from spatial linear regression (see *Methods*, section **Directionality of spike bursts and LFP discharges**). R^2 is a measure of the extent to which a burst is directional. A three-second moving average of the R^2 values is shown (*blue line*).

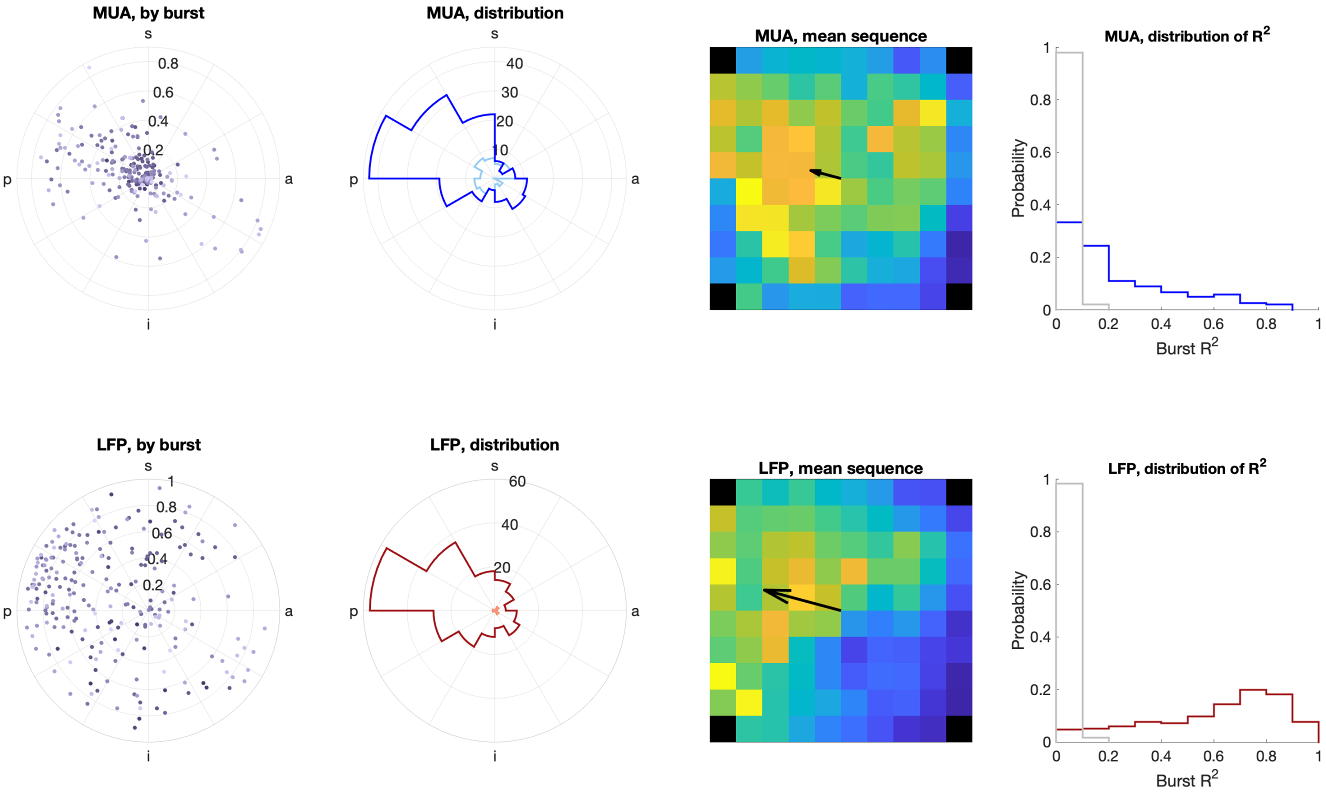

Supplementary Figure 132 Patient 5, array 1, seizure 5, seizure directionality. Top row: LFP. Bottom row: MUA. *Blue* histograms are used to designated MUA, while *maroon* histograms are used to designate LFP. First column from left: each dot represents a burst. θ reflects direction, and ρ is R^2, a measure of goodness of fit of the data to a plane. In other words, it is a measure of directionality of the burst. Second column from left: distribution direction of all recorded bursts. *Dark colors* indicate bursts with spatial linear regression giving $p \leq 0.05$, while *light* colors indicate bursts with $p > 0.05$. Third column from left: the average (*backbone*) sequence is shown over all bursts. This is obtained by taking the mean normalized rank of all sequences. The length of the *black arrow* indicates the mean R^2 over all bursts of this type, while the angle of the arrow indicates the mode of the histogram in the *second from left* column, for bursts with $p \leq 0.05$. Fourth column from left: R^2 for all bursts is shown. *Colored* histogram indicates the true sequences. *Gray* histogram, on the other hand, indicates R^2 for a set of null sequences, created by shuffling the ranks of the true sequences.

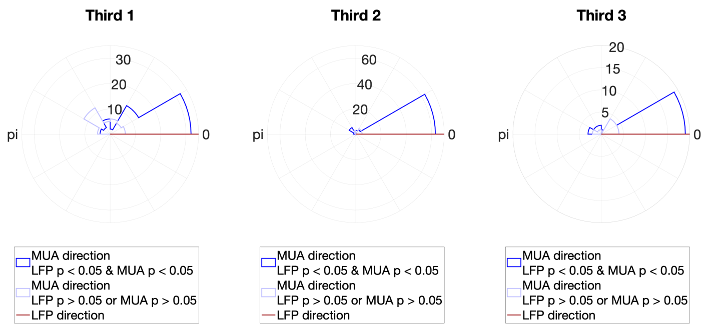

Supplementary Figure 133 Patient 5, array 1, seizure 5, relationship between MUA and LFP direction. Seizure bursts were divided into thirds (*left*: first third; *middle*: middle third, *right*: last third). For each third, we provided the distribution of burst-wise LFP-to-MUA directions. For each burst, LFP and MUA direction are compared, and the absolute value of the angular difference is put into the histogram. Bursts are divided into those with LFP and MUA p-value both less than 0.05 (*dark blue* histogram), and those with either MUA or LFP with p-value greater than 0.05. (*light blue* histogram).

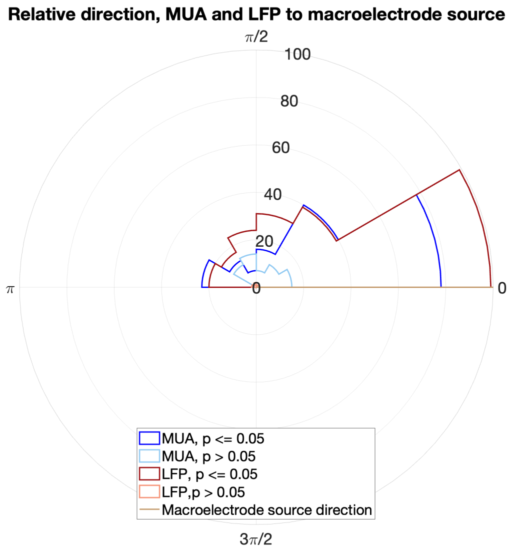

Supplementary Figure 134 Patient 5, array 1, seizure 5, relationship between discharge source, as determined by macroelectrode recordings, and direction of burst MUA and LFP. We used the time differences of the signal recorded in macroelectrodes to determine the location of the discharge source (Diamond, et al 2021, Diamond, et al 2023, see Supplementary Figure 12). The shortest path from the source to the array was then obtained, and we measured the angle of approach of the shortest path to the array (*tan* line). We then determined the relative direction of MUA and LFP signal, for each burst, to the discharge source as measured by macroelectrodes. Histograms are then provided, for the absolute value of the relative direction between source and MUA sequences (*blue*) and source and LFP sequences (*red*), for significant sequences (*dark* colors) and non-significant sequences (*light* colors).

Seizure activity, seizure 6

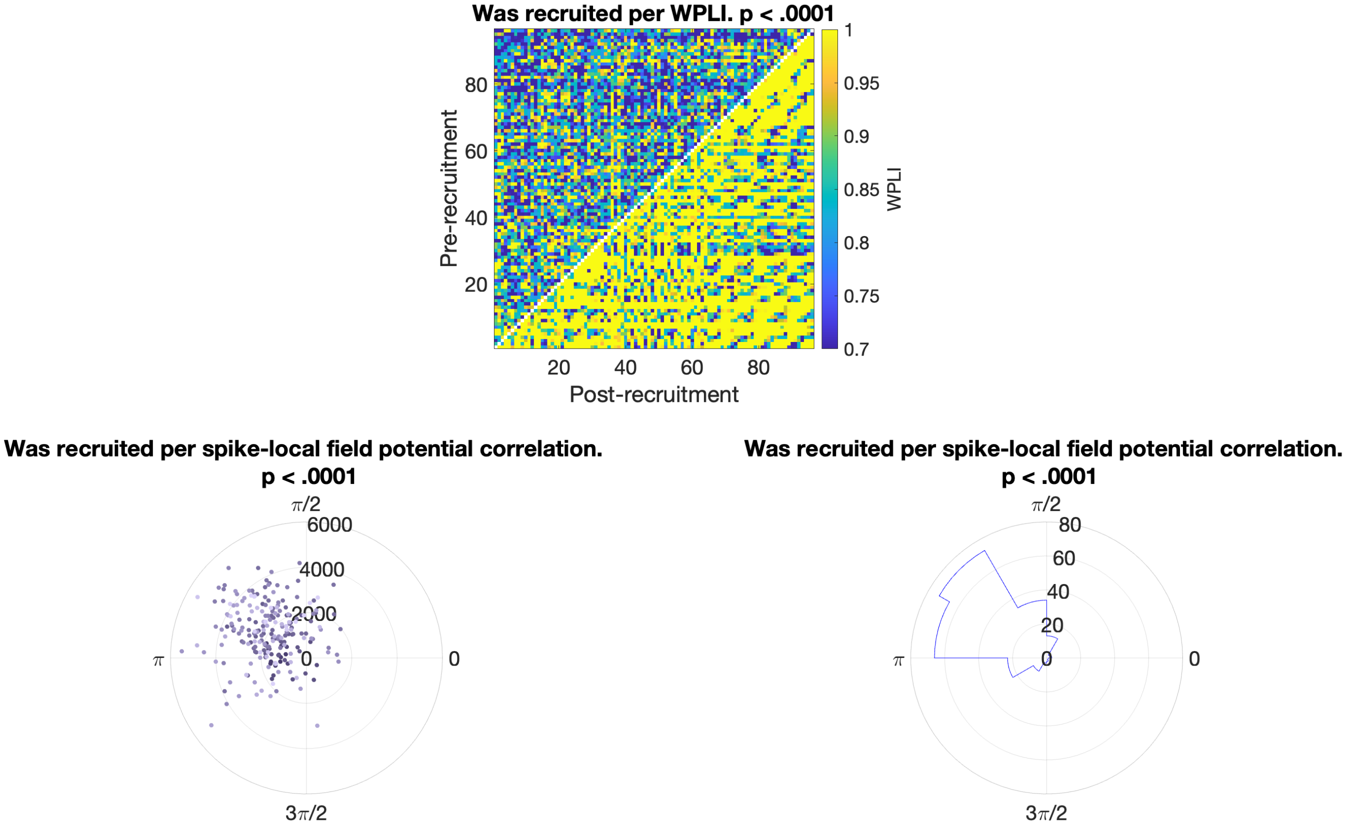

Supplementary Figure 135 Measures of recruitment for patient 5, array 1, seizure 6. Top: weighted phase lag index (WPLI), 10 seconds prior to seizure onset (top left), and 10 seconds after recruitment (bottom right, see *Methods*). Bottom left: relationship between LFP phase (θ) and LFP phase power (ρ), over the course of the seizure. *Dark purple* dots: early seizure; *light purple* dots: late seizure. Bottom right: distribution of spike-local field correlation values.

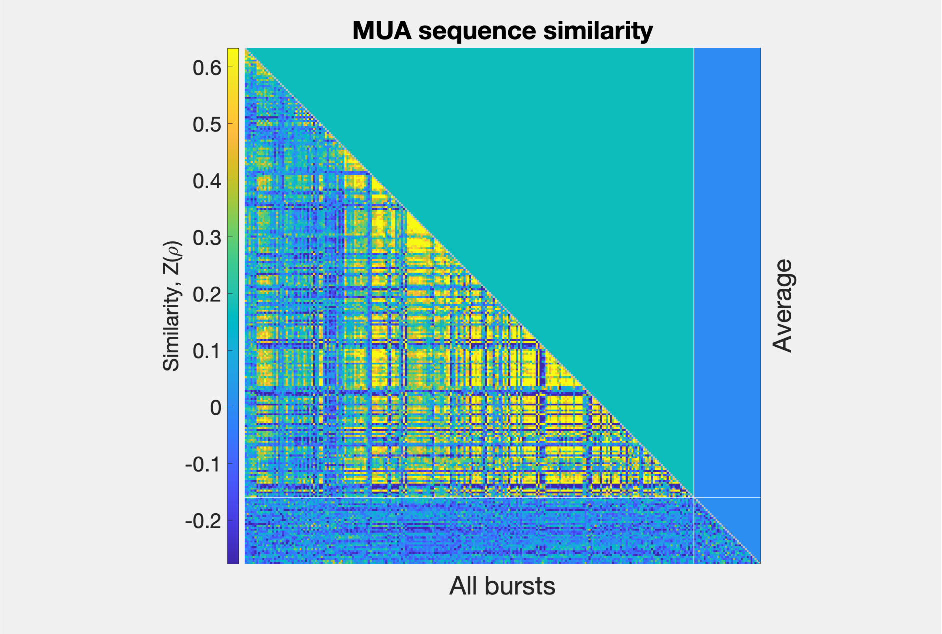

Supplementary Figure 136 Patient 5, array 1, seizure 6, burst similarity. For this patient, array, and seizure, every sequence was compared to every other sequence. Each cell indicates the similarity of the row sequence to the column sequence (Spearman's ρ). Sequences are divided by *white* lines into seizure sequences (*left, top*), IED sequences (*middle*), and baseline sequences (*right, bottom*). Actual values are provided in the lower triangle. In the upper triangle, average values are provided for each group, with values represented by the same color axis.

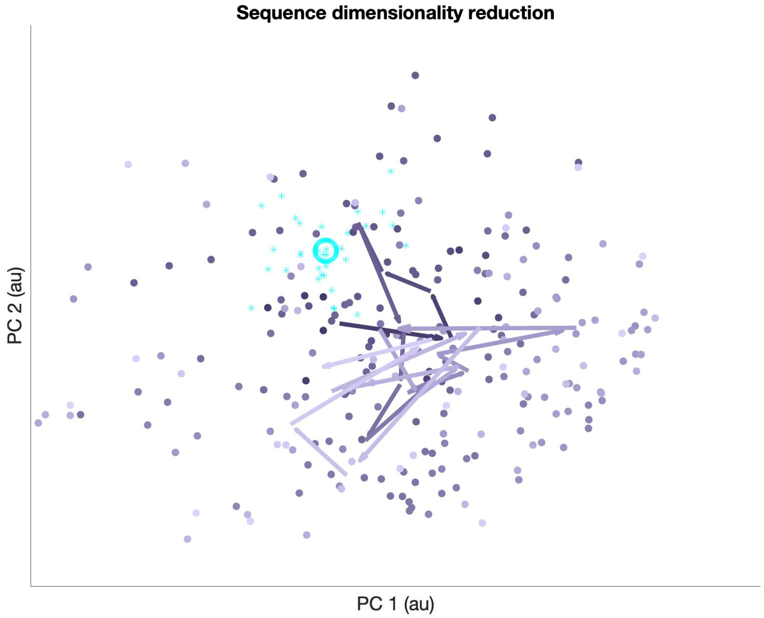

Supplementary Figure 137 Patient 5, array 1, seizure 6, dimensionality reduction using principal component analysis (PCA) was performed. We used PCA to decompose high-dimensional data into two dimensions in an unsupervised fashion (see *Methods*). Data provided include baseline bursts (*cyan stars*), IED bursts (*red dots*), and seizure bursts (*colored dots*; *dark purple*, early seizure, *light purple*, late seizure). The baseline centroid is indicated by the *cyan circle*.

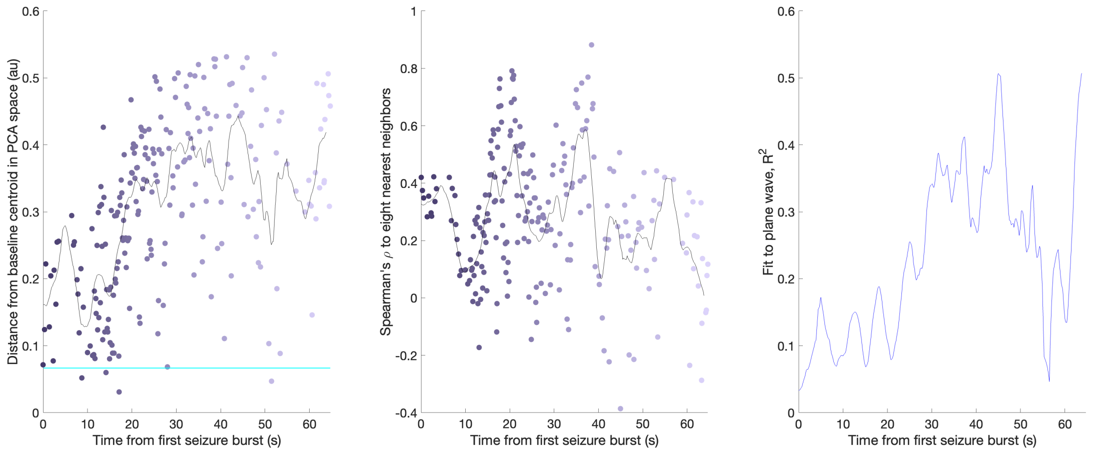

Supplementary Figure 138 Patient 5, array 1, seizure 6, measures of wave entrainment over time. *Left*: We used dimensionality reduction to collapse high-dimensional sequence information into two dimensions (see Figure 2a). We then took the location of the baseline centroid, and captured the distance, over time, between seizure bursts and the baseline centroid, in the low-dimensional manifold (see Figure 2c). *Dark purple* dots: early seizure; *light purple* dots: late seizure. A three-second moving average is superimposed (*black line*). Mean distance of baseline bursts to the baseline centroid is indicated by the *cyan line*. *Middle*: we captured Spearman's ρ between each seizure burst and its eight nearest temporal neighbors (see Figure 3a, b). This can be thought of as a measure of consistency of seizure bursts to each other in time. A three-second moving average of the ρ values is shown (*black line*). *Right*: for each burst, the R^2 value was determined from spatial linear regression (see *Methods*, section **Directionality of spike bursts and LFP discharges**). R^2 is a measure of the extent to which a burst is directional. A three-second moving average of the R^2 values is shown (*blue line*).

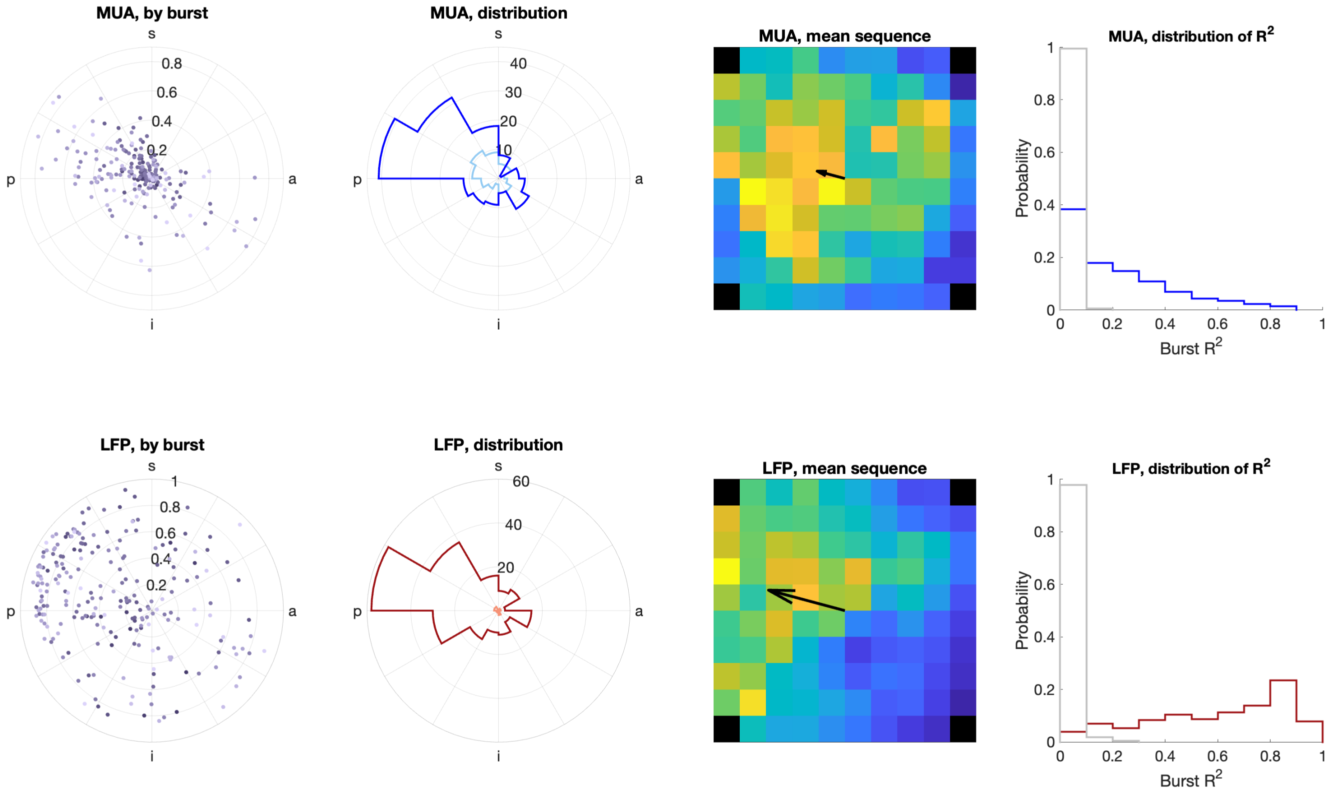

Supplementary Figure 139 Patient 5, array 1, seizure 6, seizure directionality. Top row: LFP. Bottom row: MUA. *Blue* histograms are used to designated MUA, while *maroon* histograms are used to designate LFP. First column from left: each dot represents a burst. θ reflects direction, and ρ is R^2, a measure of goodness of fit of the data to a plane. In other words, it is a measure of directionality of the burst. Second column from left: distribution direction of all recorded bursts. *Dark colors* indicate bursts with spatial linear regression giving $p \leq 0.05$, while *light* colors indicate bursts with $p > 0.05$. Third column from left: the average (*backbone*) sequence is shown over all bursts. This is obtained by taking the mean normalized rank of all sequences. The length of the *black arrow* indicates the mean R^2 over all bursts of this type, while the angle of the arrow indicates the mode of the histogram in the *second from left* column, for bursts with $p \leq 0.05$. Fourth column from left: R^2 for all bursts is shown. *Colored* histogram indicates the true sequences. *Gray* histogram, on the other hand, indicates R^2 for a set of null sequences, created by shuffling the ranks of the true sequences.

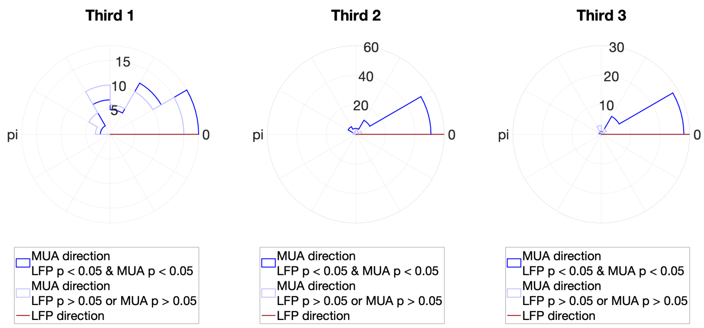

Supplementary Figure 140 Patient 5, array 1, seizure 6, relationship between MUA and LFP direction. Seizure bursts were divided into thirds (*left*: first third; *middle*: middle third, *right*: last third). For each third, we provided the distribution of burst-wise LFP-to-MUA directions. For each burst, LFP and MUA direction are compared, and the absolute value of the angular difference is put into the histogram. Bursts are divided into those with LFP and MUA p-value both less than 0.05 (*dark blue* histogram), and those with either MUA or LFP with p-value greater than 0.05. (*light blue* histogram).

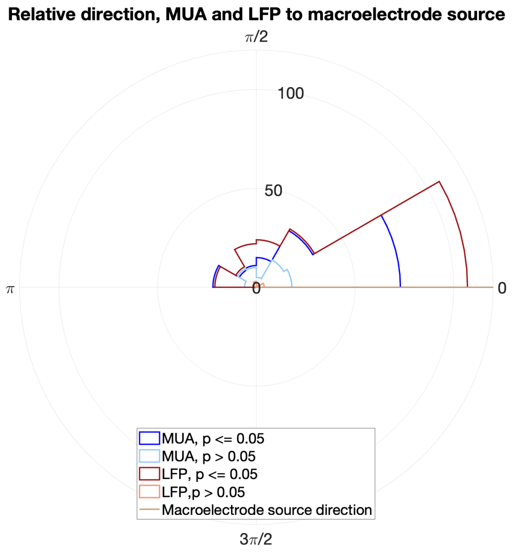

Supplementary Figure 141 Patient 5, array 1, seizure 6, relationship between discharge source, as determined by macroelectrode recordings, and direction of burst MUA and LFP. We used the time differences of the signal recorded in macroelectrodes to determine the location of the discharge source (Diamond, et al 2021, Diamond, et al 2023, see Supplementary Figure 12). The shortest path from the source to the array was then obtained, and we measured the angle of approach of the shortest path to the array (*tan* line). We then determined the relative direction of MUA and LFP signal, for each burst, to the discharge source as measured by macroelectrodes. Histograms are then provided, for the absolute value of the relative direction between source and MUA sequences (*blue*) and source and LFP sequences (*red*), for significant sequences (*dark* colors) and non-significant sequences (*light* colors).

References

[1] Berg, A. T. *et al.* Revised terminology and concepts for organization of seizures and epilepsies: report of the ilae commission on classification and terminology, 2005–2009 (2010).

[2] Fisher, R. S. *et al.* Epileptic seizures and epilepsy: definitions proposed by the international league against epilepsy (ilae) and the international bureau for epilepsy (ibe). *Epilepsia* **46**, 470–472 (2005).

[3] Schevon, C. A. *et al.* Evidence of an inhibitory restraint of seizure activity in humans. *Nature Communications* **3**, 1060 (2012).

[4] Rossi, L. F., Wykes, R. C., Kullmann, D. M. & Carandini, M. Focal cortical seizures start as standing waves and propagate respecting homotopic connectivity. *Nature communications* **8**, 1–11 (2017).

[5] Diamond, J. M. *et al.* Travelling waves reveal a dynamic seizure source in human focal epilepsy. *Brain* **144**, 1751–1763 (2021).

[6] Schlafly, E. D. *et al.* Multiple sources of fast traveling waves during human seizures: resolving a controversy. *Journal of Neuroscience* **42**, 6966–6982 (2022).

[7] Diamond, J. M. *et al.* Interictal discharges in the human brain are travelling waves arising from an epileptogenic source. *Brain* **146**, 1903–1915 (2023).

[8] Withers, C. P. *et al.* Identifying sources of human interictal discharges with travelling wave and white matter propagation. *Brain* awad259 (2023).

[9] Chowdhury, F. A., Silva, R., Whatley, B. & Walker, M. C. Localisation in focal epilepsy: a practical guide. *Practical Neurology* **21**, 481–491 (2021).

[10] Tufenkjian, K. & Lüders, H. O. Seizure semiology: its value and limitations in localizing the epileptogenic zone. *Journal of clinical neurology* **8**, 243–250 (2012).

[11] Jang, A. I., Wittig, J. H., Inati, S. K. & Zaghloul, K. A. Human Cortical Neurons in the Anterior Temporal Lobe Reinstate Spiking Activity during Verbal Memory Retrieval. *Current Biology* **27**, 1700–1705.e5 (2017). URL `http://www.cell.com/current-biology/abstract/S0960-9822(17)30550-X`.

[12] Pouget, A., Dayan, P. & Zemel, R. Information processing with population codes. *Nature Reviews Neuroscience* **1**, 125–132 (2000).

[13] Averbeck, B. B., Latham, P. E. & Pouget, A. Neural correlations, population coding and computation. *Nature Reviews. Neuroscience* **7**, 358–366 (2006).

[14] Truccolo, W. *et al.* Single-neuron dynamics in human focal epilepsy. *Nature Neuroscience* **14**, 635–641 (2011).

[15] Thorpe, S., Delorme, A. & Van Rullen, R. Spike-based strategies for rapid processing. *Neural networks* **14**, 715–725 (2001).

[16] Panzeri, S., Petersen, R. S., Schultz, S. R., Lebedev, M. & Diamond, M. E. The role of spike timing in the coding of stimulus location in rat somatosensory cortex. *Neuron* **29**, 769–777 (2001).

[17] Vaz, A. P., Wittig Jr, J. H., Inati, S. K. & Zaghloul, K. A. Replay of cortical spiking sequences during human memory retrieval. *Science* **367**, 1131–1134 (2020).

[18] Zuo, Y. *et al.* Complementary contributions of spike timing and spike rate to perceptual decisions in rat s1 and s2 cortex. *Current Biology* **25**, 357–363 (2015).

[19] Boahen, K. Dendrocentric learning for synthetic intelligence. *Nature* **612**, 43–50 (2022).

[20] Smith, E. H. *et al.* The ictal wavefront is the spatiotemporal source of discharges during spontaneous human seizures. *Nature Communications* **7**, 11098 (2016).

[21] Tong, A. P. S., Vaz, A. P., Wittig, J. H., Inati, S. K. & Zaghloul, K. A. Ripples reflect a spectrum of synchronous spiking activity in human anterior temporal lobe. *Elife* **10**, e68401 (2021).

[22] Langdon, C., Genkin, M. & Engel, T. A. A unifying perspective on neural manifolds and circuits for cognition. *Nature Reviews Neuroscience* 1–15 (2023).

[23] Cunningham, J. P. & Yu, B. M. Dimensionality reduction for large-scale neural recordings. *Nature neuroscience* **17**, 1500–1509 (2014).

[24] Martinet, L.-E. *et al.* Human seizures couple across spatial scales through travelling wave dynamics. *Nature Communications* **8**, 1–13 (2017).

[25] Smith, E. H. *et al.* Human interictal epileptiform discharges are bidirectional traveling waves echoing ictal discharges. *Elife* **11**, e73541 (2022).

[26] Liou, J.-y. *et al.* A model for focal seizure onset, propagation, evolution, and progression. *Elife* **9**, e50927 (2020).

[27] Tobochnik, S., Salami, P., Cash, S. S. & Schevon, C. A. Mechanisms of focal seizure initiation and propagation. *Neurobiology of the Epilepsies: From Epilepsy: A Comprehensive Textbook* (2022).

[28] Gelinas, J. N., Khodagholy, D., Thesen, T., Devinsky, O. & Buzsáki, G. Interictal epileptiform discharges induce hippocampal–cortical coupling in temporal lobe epilepsy. *Nature medicine* **22**, 641–648 (2016).

[29] Merricks, E. M. *et al.* Single unit action potentials in humans and the effect of seizure activity. *Brain* **138**, 2891–2906 (2015).

[30] Englot, D. J. *et al.* Impaired consciousness in temporal lobe seizures: role of cortical slow activity. *Brain* **133**, 3764–3777 (2010).

[31] Blumenfeld, H. Impaired consciousness in epilepsy. *The Lancet Neurology* **11**, 814–826 (2012).

[32] Wu, J.-Y., Huang, X. & Zhang, C. Propagating waves of activity in the neocortex: what they are, what they do. *The Neuroscientist* **14**, 487–502 (2008).

[33] Buzsaki, G., Anastassiou, C. & Koch, C. The origin of extracellular fields and currents - eeg, ecog, lfp and spikes. *Nature Reviews Neuroscience* **13**, 407–419 (2012).

[34] Muller, L., Chavane, F., Reynolds, J. & Sejnowski, T. J. Cortical travelling waves: mechanisms and computational principles. *Nature Reviews Neuroscience* **19**, 255–268 (2018).

[35] Mohan, U. R., Zhang, H., Ermentrout, B. & Jacobs, J. The direction of theta and alpha travelling waves modulates human memory processing. *Nature Human Behaviour* 1–12 (2024).

[36] Bragina, A., Csicsvari, J., Penttonen, M. & Buzsáki, G. Epileptic afterdischarge in the hippocampal–entorhinal system: current source density and unit studies. *Neuroscience* **76**, 1187–1203 (1997).

[37] Ponce-Alvarez, A., Jouary, A., Privat, M., Deco, G. & Sumbre, G. Whole-brain neuronal activity displays crackling noise dynamics. *Neuron* **100**, 1446–1459 (2018).

[38] Burrows, D. R. *et al.* Microscale neuronal activity collectively drives chaotic and inflexible dynamics at the macroscale in seizures. *Journal of Neuroscience* **43**, 3259–3283 (2023).

[39] Jefferys, J. G. R. Nonsynaptic modulation of neuronal activity in the brain: Electric currents and extracellular ions. *Physiological Reviews* **75**, 689–723 (1995).

[40] McCormick, D. A. & Contreras, D. On the cellular and network bases of epileptic seizures. *Annual review of physiology* **63**, 815–846 (2001).

[41] Codadu, N. K., Parrish, R. R. & Trevelyan, A. J. Region-specific differences and areal interactions underlying transitions in epileptiform activity. *The Journal of Physiology* **597**, 2079–2096 (2019).

[42] Braitenberg, V. & Schüz, A. *Cortex: statistics and geometry of neuronal connectivity* (Springer Science & Business Media, 2013).

[43] Engel Jr, J. *Seizures and epilepsy*, vol. 83 (Oxford University Press, 2013).

[44] Bragin, A., Penttonen, M. & Buzsáki, G. Termination of epileptic afterdischarge in the hippocampus. *Journal of Neuroscience* **17**, 2567–2579 (1997).

[45] Lian, J., Bikson, M., Shuai, J. & Durand, D. M. Propagation of non-synaptic epileptiform activity across a lesion in rat hippocampal slices. *The Journal of physiology* **537**, 191 (2001).

[46] Zhang, M. *et al.* Propagation of epileptiform activity can be independent of synaptic transmission, gap junctions, or diffusion and is consistent with electrical field transmission. *Journal of Neuroscience* **34**, 1409–1419 (2014).

[47] Bringuier, V., Chavane, F., Glaeser, L. & Frégnac, Y. Horizontal propagation of visual activity in the synaptic integration field of area 17 neurons. *Science* **283**, 695–699 (1999).

[48] Schwartz, T. H. & Bonhoeffer, T. In vivo optical mapping of epileptic foci and surround inhibition in ferret cerebral cortex (2001).

[49] Weiss, S. A. *et al.* Fast ripples reflect increased excitability that primes epileptiform spikes. *Brain Communications* (2023).

[50] Davey, J. *et al.* Exploring the role of the posterior middle temporal gyrus in semantic cognition: Integration of anterior temporal lobe with executive processes. *Neuroimage* **137**, 165–177 (2016).

[51] Chao, L., Haxby, J. & Martin, A. Attribute-based neural substrates in temporal cortex for perceiving and knowing about objects. *Nature Neuroscience* **2**, 913–919 (1999).

[52] Ralph, M. A. L., Jefferies, E., Patterson, K. & Rogers, T. T. The neural and computational bases of semantic cognition. *Nature Reviews Neuroscience* **18**, 42–55 (2017).

[53] Mitra, P. *Observed Brain Dynamics* (Oxford University Press, 2007).

[54] Berens, P. Circstat: a matlab toolbox for circular statistics. *Journal of statistical software* **31**, 1–21 (2009).

[55] Diamond, J. M., Chapeton, J. I., Theodore, W. H., Inati, S. K. & Zaghloul, K. A. The seizure onset zone drives state-dependent epileptiform activity in susceptible brain regions. *Clinical Neurophysiology* **130**, 1628–1641 (2019).

[56] Proix, T., Jirsa, V. K., Bartolomei, F., Guye, M. & Truccolo, W. Predicting the spatiotemporal diversity of seizure propagation and termination in human focal epilepsy. *Nature Communications* **9**, 1–15 (2018).

[57] Perucca, P., Dubeau, F. & Gotman, J. Intracranial electroencephalographic seizure-onset patterns: effect of underlying pathology. *Brain* **137**, 183–196 (2014).

[58] Martinet, L.-E., Ahmed, O. J., Lepage, K. Q., Cash, S. S. & Kramer, M. A. Slow spatial recruitment of neocortex during secondarily generalized seizures and its relation to surgical outcome. *Journal of Neuroscience* **35**, 9477–9490 (2015).

[59] Kramer, M. A. & Cash, S. S. Epilepsy as a disorder of cortical network organization. *The Neuroscientist* **18**, 360–372 (2012).

[60] Malinowska, U. *et al.* Interictal networks in magnetoencephalography. *Human brain mapping* **35**, 2789–2805 (2014).

[61] Liou, J.-y. *et al.* Multivariate regression methods for estimating velocity of ictal discharges from human microelectrode recordings. *Journal of neural engineering* **14**, 044001 (2017).

[62] JASP Team. JASP (Version 0.18.3)[Computer software] (2024). URL `https://jasp-stats.org/`.

[63] Rosenthal, R. & DiMatteo, M. R. Meta-analysis: Recent developments in quantitative methods for literature reviews. *Annual review of psychology* **52**, 59–82 (2001).
