# Supplementary material for: Focal seizures induce spatiotemporally organized spiking activity in the human cortex
Source: Nat Commun. 2024 Aug 16;15:7075. doi: 10.1038/s41467-024-51338-1 (PMC11329741; doi:10.1038/s41467-024-51338-1)
Supplement: Supplementary file 3 — Reporting Summary [file 41467_2024_51338_MOESM3_ESM.pdf]

nature portfolio

Corresponding author(s): Kareem Zaghloul

Last updated by author(s): Apr 8, 2024

Reporting Summary

Nature Portfolio wishes to improve the reproducibility of the work that we publish. This form provides structure for consistency and transparency in reporting. For further information on Nature Portfolio policies, see our Editorial Policies and the Editorial Policy Checklist.

Statistics

For all statistical analyses, confirm that the following items are present in the figure legend, table legend, main text, or Methods section.

n/a	Confirmed	
☐	☒	The exact sample size (*n*) for each experimental group/condition, given as a discrete number and unit of measurement
☐	☒	A statement on whether measurements were taken from distinct samples or whether the same sample was measured repeatedly
☐	☒	The statistical test(s) used AND whether they are one- or two-sided<br>*Only common tests should be described solely by name; describe more complex techniques in the Methods section.*
☐	☒	A description of all covariates tested
☐	☒	A description of any assumptions or corrections, such as tests of normality and adjustment for multiple comparisons
☐	☒	A full description of the statistical parameters including central tendency (e.g. means) or other basic estimates (e.g. regression coefficient) AND variation (e.g. standard deviation) or associated estimates of uncertainty (e.g. confidence intervals)
☐	☒	For null hypothesis testing, the test statistic (e.g. *F*, *t*, *r*) with confidence intervals, effect sizes, degrees of freedom and *P* value noted<br>*Give P values as exact values whenever suitable.*
☒	☐	For Bayesian analysis, information on the choice of priors and Markov chain Monte Carlo settings
☐	☒	For hierarchical and complex designs, identification of the appropriate level for tests and full reporting of outcomes
☐	☒	Estimates of effect sizes (e.g. Cohen's *d*, Pearson's *r*), indicating how they were calculated

Our web collection on statistics for biologists contains articles on many of the points above.

Software and code

Policy information about availability of computer code

Data collection	We recorded micro-electrode signals at 30 kHz using a Cerebus acquisition system (Blackrock Microsystems), with 16-bit precision and a range of +/- 8 mV.<br><br>Data collection was also carried out using custom scripts built in MATLAB (Mathworks, Natick, MA), version R2023b.
Data analysis	We analyzed data using custom MATLAB scripts (Mathworks, Natick, MA), version R2023b.

For manuscripts utilizing custom algorithms or software that are central to the research but not yet described in published literature, software must be made available to editors and reviewers. We strongly encourage code deposition in a community repository (e.g. GitHub). See the Nature Portfolio guidelines for submitting code & software for further information.

Data

Policy information about availability of data

All manuscripts must include a data availability statement. This statement should provide the following information, where applicable:

- Accession codes, unique identifiers, or web links for publicly available datasets
- A description of any restrictions on data availability
- For clinical datasets or third party data, please ensure that the statement adheres to our policy

Processed data and code used for analysis are publicly available for download at https://research.ninds.nih.gov/zaghloul-lab/downloads and with the manuscript. Because of size constraints, raw data are not provided but are available upon request.

Research involving human participants, their data, or biological material

Policy information about studies with human participants or human data. See also policy information about sex, gender (identity/presentation), and sexual orientation and race, ethnicity and racism.

Reporting on sex and gender	Our study considers five males. Sex was determined based on self-reporting; gender was not considered in this study. The inclusion of only males was incidental, and arose from the fact that only a subset (consisting of five males) of our patients had usable ictal microelectrode data, even though both males and females are treated in our epilepsy protocols.<br><br>Demographic information for patients is provided in Table 1 of the manuscript.<br><br>While we would not expect our findings to be influenced by gender in any meaningful way, we cannot make this claim with certainty as our study only includes male patients.
Reporting on race, ethnicity, or other socially relevant groupings	Socially-constructed variables were not considered in this analysis. This information was not requested from our patients, and was not reported in our analyses.
Population characteristics	See above.
Recruitment	Patients are recruited to the NIH based on candidacy for our protocol (must have drug-resistant epilepsy). Participants were recruited by self-referral, and by referral from healthcare providers in the community and nationwide. Our center provides free treatment irrespective of insurance status, and so we recruit patients across the spectrum of socio-economic status. So, we do not expect substantial bias stemming from referral patterns or self-selection.
Ethics oversight	The study was approved and supervised by the IRB at the Clinical Center at the National Institutes of Health (protocol reference number 11-N-0051).

Note that full information on the approval of the study protocol must also be provided in the manuscript.

Field-specific reporting

Please select the one below that is the best fit for your research. If you are not sure, read the appropriate sections before making your selection.

☒ Life sciences ☐ Behavioural & social sciences ☐ Ecological, evolutionary & environmental sciences

For a reference copy of the document with all sections, see nature.com/documents/nr-reporting-summary-flat.pdf

Life sciences study design

All studies must disclose on these points even when the disclosure is negative.

Sample size	No upfront sample size calculation was performed. The sample size was chosen simply by considering the patients who have underwent invasive monitoring with video-iEEG at our institution, and selecting those who had usable ictal microelectrode array (MEA) recordings.<br><br>We considered each MEA as an independent sample for our analyses since they recorded spiking activity from different populations of neurons.<br><br>We had five patients with usable MEA recordings, comprising six MEAs. Therefore, the sample size in our study was six.
Data exclusions	Patients were excluded if recordings were unusable (excess artifact, insufficient signal). One patient was excluded from this study on the basis that no seizures 'recruited' the microelectrode array. This exclusion is in line with exclusions carried out in similar work in the past (Smith, et al ELife 2022). Our analyses are impossible without microelectrode array recruitment, and so this exclusion was mandatory.
Replication	Our study is retrospective, so prospective replication of the results is not feasible. However, we do use statistical tests to ensure that trends across patients observed in our data are robust. These are described in the "Statistical Analyses" subsection of our Methods section.

Randomization	Our study is retrospective, and so there was no randomization or allocation of subjects into groups.
Blinding	There was no allocation of subjects into groups in this study.

Reporting for specific materials, systems and methods

We require information from authors about some types of materials, experimental systems and methods used in many studies. Here, indicate whether each material, system or method listed is relevant to your study. If you are not sure if a list item applies to your research, read the appropriate section before selecting a response.

Materials & experimental systems

n/a	Involved in the study
☒	☐ Antibodies
☒	☐ Eukaryotic cell lines
☒	☐ Palaeontology and archaeology
☒	☐ Animals and other organisms
☒	☐ Clinical data
☒	☐ Dual use research of concern
☒	☐ Plants

Methods

n/a	Involved in the study
☒	☐ ChIP-seq
☒	☐ Flow cytometry
☒	☐ MRI-based neuroimaging

Plants

Seed stocks	*Report on the source of all seed stocks or other plant material used. If applicable, state the seed stock centre and catalogue number. If plant specimens were collected from the field, describe the collection location, date and sampling procedures.*
Novel plant genotypes	*Describe the methods by which all novel plant genotypes were produced. This includes those generated by transgenic approaches, gene editing, chemical/radiation-based mutagenesis and hybridization. For transgenic lines, describe the transformation method, the number of independent lines analyzed and the generation upon which experiments were performed. For gene-edited lines, describe the editor used, the endogenous sequence targeted for editing, the targeting guide RNA sequence (if applicable) and how the editor was applied.*
Authentication	*Describe any authentication procedures for each seed stock used or novel genotype generated. Describe any experiments used to assess the effect of a mutation and, where applicable, how potential secondary effects (e.g. second site T-DNA insertions, mosiacism, off-target gene editing) were examined.*
