# Supplementary material for: Focal seizures induce spatiotemporally organized spiking activity in the human cortex
Source: Nat Commun. 2024 Aug 16;15:7075. doi: 10.1038/s41467-024-51338-1 (PMC11329741; doi:10.1038/s41467-024-51338-1)
Supplement: Supplementary file 4 — Source Data [file 41467_2024_51338_MOESM4_ESM.zip › Readme.rtf]

April 7, 2024Written by: Joshua Diamond (diamondjm@nih.gov)Senior author: Kareem Zaghloul (kareem.zaghloul@nih.gov)Provided in this repository are the files and code for replicating the analysis in Diamond, et al. Focal seizures induce spatiotemporally organized spiking activity in the human cortex. I would expect no dependencies and I would expect this to work on Mac or Windows. It has been tested on MATLAB R2023b, on macOS Sonoma 14.4. By running main.m, all of the figures in the main article, supplementary figures, and supplementary appendix will be generated. No install is required. Run-time should be no more than 5-10 minutes. These figures are produced from the file allResults.mat, which itself contains all the processed data which is used in figure production. Raw data, on the other hand, is not included because of size constraints but is available upon request. Most of the code in the present project is included in a class called microOutputResults. The principle method of the class, moreover, is called collectAndProcess. It is collectAndProcess which initially analyzes and saves data. This function also creates figures. For initial data analysis or re-analysis, collectAndProcess is run with the ‘forceNew’ flag on. In the present repository, this flag cannot be turned on, because doing so requires access to the raw data which is not included in the repository. Please contact the authors for access to the raw data. 
